# Bacteria in the oral cavity of individuals consuming intoxicating substances

**DOI:** 10.1371/journal.pone.0285753

**Published:** 2023-05-26

**Authors:** Reyaz Ahmad Khan, Kangjam Rekha Devi, Manash Pratim Barman, Madhusmita Bhagawati, Rajeev Sarmah

**Affiliations:** 1 Department of Microbiology, Faculty of Science, Assam Down Town University, Guwahati, Assam, India; 2 Regional Medical Research Centre-Indian Council of Medical Research, Dibrugarh, Assam, India; 3 Department of Statistics, Dibrugarh University, Dibrugarh, Assam, India; 4 Department of Microbiology, Shrimanta Shankardeva University of Health Science, Guwahati, Assam, India; 5 Department Biotechnology, Faculty of Science, Assam Down Town University, Guwahati, Assam, India; University of Jeddah, SAUDI ARABIA

## Abstract

Food habits and oral hygiene are critical attributes for physiochemical environment of the oral cavity. Consumption of intoxicating substances such as betel nut (’Tamul’), alcohol, smoking and chewing tobacco may strongly influence the oral ecosystem including commensal microbes. Therefore, a comparative assessment of microbes in the oral cavity between individuals consuming intoxicating substances and non-consumers may indicate the influence of these substances. Oral swabs were collected from consumers of intoxicating substances and non- consumers of Assam, India, microbes were isolated by culturing on Nutrient agar and identified by phylogenetic analysis of their 16S rRNA gene sequences. The risks of consumption of intoxicating substance on occurrence of microbes and health conditions were estimated using binary logistic regression. Mostly pathogens and opportunistic pathogens were found in the oral cavity of consumers and oral cancer patients which included *Pseudomonas aeruginosa*, *Serratia marcescens*, *Rhodococcus antrifimi*, *Paenibacillus dendritiformis*, *Bacillus cereus*, *Staphylococcus carnosus*, *Klebsiella michiganensis* and *Pseudomonas cedrina*. *Enterobacter hormaechei* was found in the oral cavity of cancer patients but not in other cases. *Pseudomonas sp*. were found to be widely distributed. The risk of occurrence of these organisms were found in between 0.01 and 2.963 odds and health conditions between 0.088 and 10.148 odds on exposure to different intoxicating substances. When exposed to microbes, the risk of varying health conditions ranged between 0.108 and 2.306 odds. Chewing tobacco showed a higher risk for oral cancer (10.148 odds). Prolonged exposure to intoxicating substances conduce a favorable environment for the pathogens and opportunistic pathogens to colonize in the oral cavity of individuals consuming intoxicating substances.

## Introduction

Millions of people across the globe are consuming intoxicating substances such as smoking tobacco and alcohol. A significant section is indigenous to India [1] mainly the North East (NE) states, including Assam. The commonly consumed intoxicating substances include raw betel quid (’Tamul pan’) [2], gutkha (processed betel nut), alcohol and tobacco (chewing and smoking). These substances contain bio-molecules such as benzenoids, arecoline, safrole, terpenes, acids, aldehydes, alcohols, esters, and alkaloids [3]. Some are genotoxic or irritant and likely change the physiochemical characteristics of the oral cavity thereby intriguing situations for diseases, attracting non-oral microbes thus determining the microbial communities [4]. It is well known that smoking and alcohol related carcinogens are activated by oral microbes leading to oral and certain gastrointestinal cancers [5, 6] by converting ethanol to acetaldehyde, a genotoxin [7] or activating tobacco-specific nitrosamines [8]. Smoking harms oral health by affecting response to treatments [9], causing frequent nausea [10], facilitating adhesion of *C*. *albicans* leading to oral thrush [11, 12], inducing lesion [13, 14], mouth ulcers [15] and causing cellulitis [16]. Alcohol consumption is associated with osteomyelitis [17], impaired vision especially color vision [18, 19], induces drowsiness [20], weakness, myalgia and anorexia [21, 22]. Betel nut consumption is linked to insomnia, nausea [2, 23, 24], periodontitis [25–28], regulating appetite [29]. Chewing gutkha (processed betel nut) affects the oral mucosal lining and soft and hard tissues [15], causing tooth decay and gingival recession by loosing periodontal connective tissue fibers [30]. Long term consumption of intoxicating substances probably shifts the transitory pathogens to colonize, decreasing the number of commensal organisms [31–33] or triggers the opportunistic pathogens to infect the host eventually leading to various health problems including diseases such as chronic periodontitis and oral cancer [34]. The colonization of transitory and opportunistic pathogens may also be supported by the compromised immune system of an individual. On the other hand, the commensal microbes help the host by regulating homeostasis, enhancing the immune system and defending from pathogens [35].

Molecular approaches used for identification and characterization of bacterial species have demonstrated that bacterial profiles in the smokers is diverse and different from non smokers [36–38] which may also vary because of geography, population, social status etc. The colonizers and pioneer microbes when flourish their metabolic activities influence the physicochemical conditions such as redox potential, pH, nutrient availability and coaggregation and enable fastidious organisms to colonize after them [4]. Over the period of time, generally the other microbial communities take over including *Fusobacterium nucleatum*, *Veillonella*, *Prevotella melaninogenica* and *Neisseria* [39]. As the oral cavity of humans is exposed to food, air and water, it encounters a wide range of microbes those may colonize the surface of the tongue, teeth, gingiva, cheeks, gums, lips and soft and hard palate [40]. However, colonization will depend on the amicable or antagonistic physiochemical conditions and is also affected by an individual’s dietary habits and oral hygiene [41]. Based on above it can be assumed that the microbes in the oral cavity of regular, frequent and prolonged consumers of intoxicating substances differs from non-consumers and the poor oral hygiene attracts or increases the incidence of pathogenic organisms which contributes to diseases or health conditions. In connection to this, swab samples were collected from the oral cavity of the consumers of intoxicating substances and non-consumers of Assam. An analysis was conducted to evaluate the risk of consumption on the health problems and occurrence of microbes.

## Materials and methods

### Study design

A cross sectional study was conducted among the consumers of intoxicating substances and non consumers of Assam, India. The microbes in the oral cavity of both the groups were isolated cultured and identified. Using the data on consumption, microbes identified and health conditions, a risk relationship between intoxicating substances, oral microbes and health conditions was determined. The health parameters (acute cellulites, anorexia, appetite condition, arthralgia, drowsiness, gingivitis, granuloma, headache, insomnia, mouth ulceration, myalgia, nausea, oral cancer, oral thrush, osteomyelitis, periodontitis and vision) considered in this study were the common health problems, oral health and health issues reported to be linked with consumption of intoxicating substances.

### Study population and sample size

Based on the population size record of Assam, the sample size was determined using Raosoft which is 271 and 385 at 90% and 95% confidence respectively. Individuals below 18 and above 60 years, pregnant women and individuals undergoing antibiotic therapy were excluded from the study. Appropriate ethical guidelines were followed while sampling. Swab samples were collected from 211 individuals consuming intoxicating substances and 89 non consumers aged 18 to 60 years from rural and urban places of Assam. Among the consumers, 15 were diagnosed with oral cancer undergoing treatment in the Northeast Cancer Hospital and Research Institute (NECHRI), Guwahati. The participants were interviewed and information on life-style, consumption of intoxicating substances like betel nut, gutkha, tobacco etc. frequency of consumption and health issues were documented. Participants were informed about the study and received written consent for the purpose of sampling.

### Sample collection

Study participants rinsed their mouth with sterile water for 20 sec and samples were taken in swab collection tubes (PW1280) by scrubbing the either side of cheeks, gums, and tongue with a sterile cotton swab. Samples were transported to microbiological laboratory in a Thioglycollate broth.

#### Microbiological examination

Samples were inoculated on Nutrient agar and incubated for 24–48 hours at 37°C. The bacterial flora was tentatively identified by colony morphology of bacteria, growth on culture media and ‘Gram’ staining.

### DNA extraction

DNA extraction with HipurA bacterial genomic DNA purification kit was performed according to the manufacturer’s instructions (Himedia). The pellet was suspended in lysozyme solution incubated at 37ºC for 45 min. To this suspension, 25 μl of proteinase K solution (20 mg/ml), 25 μl RNase solution and 200 μl lysis solution were added and then incubated at 55ºC for 10 min. 200 μl of ethanol was added followed by procedures prescribed by the manufacturer. The extracted DNA was stored at -20ºC.

#### 16S rRNA gene amplification

The 16S rRNA gene was amplified using universal primer set 27F (5'-AGAGTTTGATCCTGGCTCAG-3') and 1492R (5'-GGTTACCTTGTTACGACTT-3') [42] supplied by Sigma Aldrich chemical Pvt. Ltd. Bangalore. PCR was performed in PCR tubes with a GeneAmp PCR system 9700 (ABI Foster city, US). 5 μl of Template DNA was added to a reaction mixture (final volume, 50 μl) containing 25 μl of GoTaq Hot Start Colorless Master Mix (Promega), 1 μl of each primer (10 pmol) and 18μl of nuclease free water. Thermal cycling consists of initial denaturation at 95ºC for 5 min, followed by 35 cycles of denaturation at 95ºC for 1 min, annealing at 55ºC for 45 s and elongation at 72ºC for 1 min, with final elongation at 72ºC for 10 min. Quality check (QC) of amplified products was done by electrophoresis (2% agarose gel) run at 60 volts for 1 hour and the expected band size was 1500bp. QC passed amplified products were purified using QIAquick PCR Purification Kit (QIAGEN).

#### 16S rRNA gene sequencing

Purified PCR products were sequenced in Applied Biosystems^™^ MiniAmp^™^ Plus Thermal cycler using Big Dye^™^ Terminator V3.1 kit. The same primers were used for sequencing. Quarter dye chemistry was used with 1 μl (~2.5 pmol) primer, 2μl (~50ng DNA) and 7μl master mix in a final volume of 10 μl. Cycle sequencing was performed with Applied Biosystems^™^ MiniAmp^™^ Plus Thermal cycler with initial denaturation at 95ºC for 3 min followed by 35 cycles of denaturation at 95ºC for 30 s, annealing at 55ºC for 30 s and extension at 72ºC for 45 s with final extension at 72ºC for 3 min. The primer extended products were purified and the sequencing reactions were run on an Applied Biosystems 3730xl (96- Capillary Array DNA Sequencer).

### Data analysis

The threshold of average quality value (QV) is an established metric for determining quality sequencing data. QV>20 means the probability that the base was miscalled is not greater than 1%, is the acceptable standard for a good sequence reaction. The raw sequencing data was visualized using Chromas V2.6.6 and low-quality peaks were trimmed from 5’ and 3’ ends [43]. The resultant peaks were then converted into fasta format files and sample wise forward and reverse sequences were assembled into contigs using CAP3 contig assembly program [44]. To classify the resultant contigs based on the sequence similarity, blast analysis has been performed [45].

#### Phylogenetic analysis

The 16S rRNA gene sequences were subjected to a BLASTn search using the default parameters and highly similar/identical nucleotide sequences were considered for naming (generic epithet) of the microorganisms. Evolutionary trees were developed to identify them upto species level. The nucleotide sequences were deposited in GenBank. The changes in microbial communities from non-consumer to consumer of intoxicating substances were then observed by phylogenetic analysis. Phylogenetic trees of related sequences were generated using the MEGA X. Maximum Likelihood approach and the Tamura Nei model to infer the evolutionary history. The initial trees for the heuristic search were generated automatically using the Neighbor Join and BioNJ algorithms on a matrix of pairwise distances using the Tamura Nei model.

### Statistical analysis

The data was processed and analyzed using IBM SPSS 21 software. To determine the relationship between a dependent and independent variables, Odds Ratio was computed by Binary Logistic Regression. The odds reflect the relationship between exposure and outcome or the risk of exposure. The backward Wald method was used to remove the independent variables that did not significantly contribute to the regression.

### Ethical clearance

This manuscript is an outcome of the study entitled "Flora in the oral cavity of pan and non chewers using conventional and molecular methods" approved by Assam down town University ethics committee. Later, the title was reframed as "Assessment of bacterial flora in the oral cavity of ’pan ’chewers and non chewers using conventional and molecular method" and duly registered on 27/07/2021. Swab sample collection from cancer patients was approved by the North East Cancer Hospital and Research Institute (NECHRI) Guwahati, Assam vide letter no: IEC/2018/06/NP/11 dated 27/08/2018. Prior to sample collection all participants duly filled consent forms in English or Assamese.

## Results

### Status of consumption of intoxicating substances

**Fig 1:**
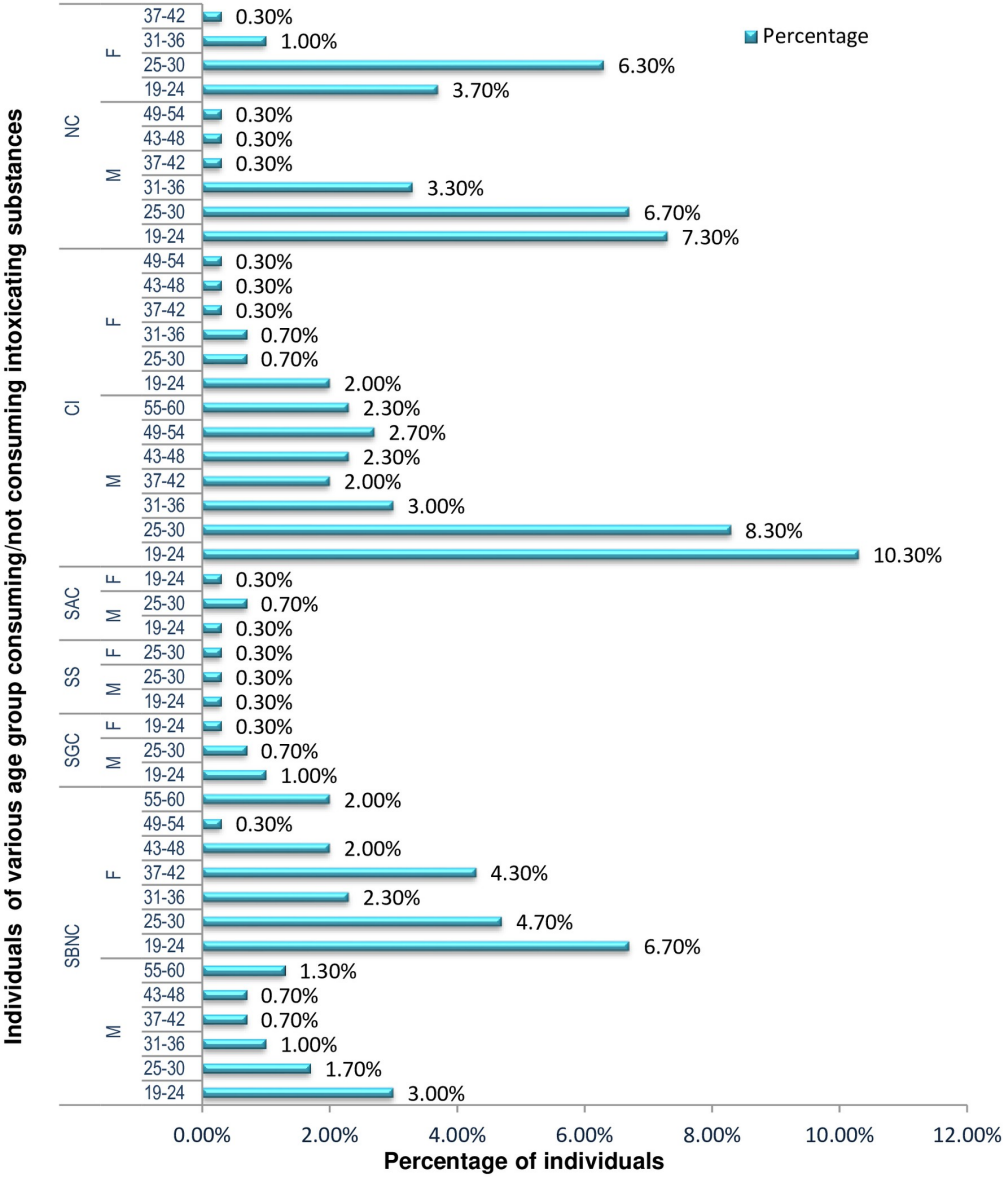
Descriptive statistics of the samples/participants of different age groups, consumers and non-consumers of intoxication substances^1^ (excluding the class of age in which sampling could not be done because of unavailability of participants). ^1^SNBC-Sole Betel Nut consumers, SGC-Sole Gutkha consumers, SS-Sole Smokers, SAC-Sole alcohol consumers, CI-Combined intoxication, NC-Non consumers; M-Male, F- Female.

Among 300 individuals, 70.3% were under the influence of one or more intoxicating substances, information regarding consumption of various intoxicating substance are provided in Fig 1, S2 and S6 Tables About 30.7% of the individuals were found to chew betel nut and leaves (’Tamul pan’), and the habit is widespread regardless of economic status. In contrast, consuming processed betel nut (’gutkha’) was found to be 2% primarily observed among the lower income groups. Individuals between 19 to 24 years are the major consumers of intoxicating substances; when the person grows older; their desire for intoxicating substances reduces. Information on oral cavity cleanliness, consumption per day/week/month, health conditions and diseases are provided in S3, S4 and S7 Tables respectively. It has been observed that health problems are associated with poor oral hygiene and aging. The oral cancer patients (5%) had consumed one or more intoxicating substances before diagnosis. Among the participants 29.7% were non consumers.

### Identification of the microbes

The isolated organisms were tentatively identified as *Staphylococcus*, *Bacillus*, *Klebsiella*, *Serratia*, *Enterobacter*, *Acinetobacter*, *Pseudomonas*, *Rhodococcus* and *Candida*. Information regarding the isolated organisms is provided in S1 Fig and S5 Table. Fifteen (15) of the 34 isolates were identified upto species level; information is provided in S2 Fig (i-xxxiv) and listed below:

**Table:** 

Sample No	GenBank AC	Name of the organism	Seq. read length	Seq.QV
141C	OL321134	*Pseudomonas aeruginosa*	763	48
143A	OL347867	*Bacillus cereus*	752	49
150B	OL347894	*Paenibacillus dendritiformis*	810	47
150C	OL347932	*Staphylococcus carnosus*	785	44
157A	OL348271	*Rhodococcus antrifimi*	781	45
162A	OL348325	*Klebsiella michiganensis*	742	48
168B	OL348482	*Serratia marcescens*	822	48
186B	OL351261	*Acinetobacter junii*	777	46
205A	OL355135	*Enterobacter asburiae*	568	37
282D	OL355153	*Serratia marcescens*	768	48
287E	OL374166	*pseudomonas cedrina*	584	39
294B	OL374127	*Enterobacteriaceae bacterium*	830	50
361A	OL375166	*Staphylococcus epidermidis*	701	41
383A	OL375171	*Serratia nematodiphila*	772	46
397B	OL375218	*Enterobacter hormaechei*	763	48

**Fig 2:**
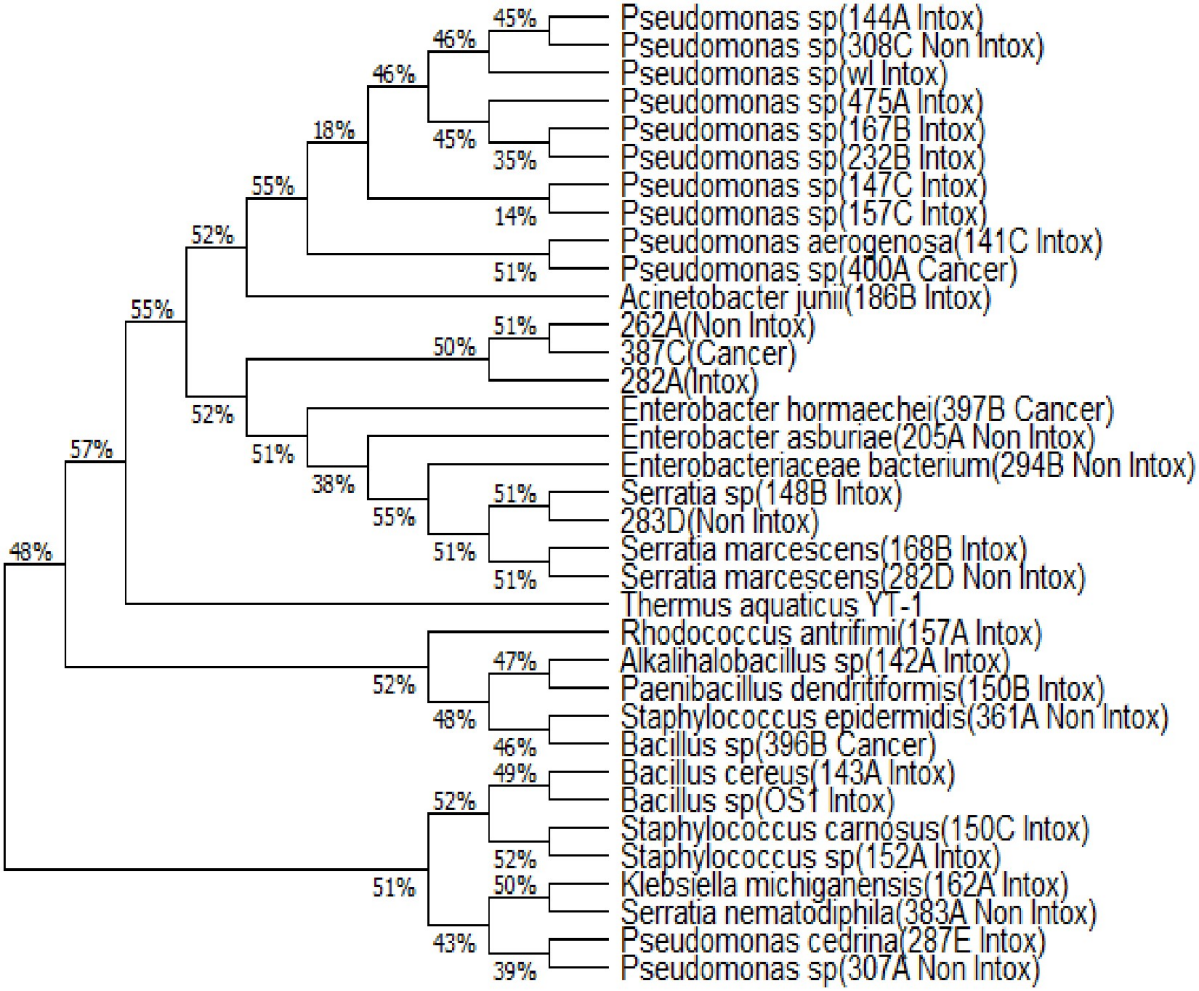
Phylogenetic tree depicting relationship among the microorganisms of the oral cavities of individuals exposed to various intoxication substances (consumers/Intox), non-consumers (Non Intox) and cancer patients.

Fifteen (15) of them were identified upto genus level which includes *Pseudomonas*, *Bacillus*, *Alkalihalobacillus*, *Serratia*, *Staphylococcus* and 4 of them (262A|OL355150, 282A|OL355152, 283D|OL374164, 387C|OL375175) could not be identified (S2 Fig (i-xxxiv)). Based on the Phylogenetic tree, it can be inferred that the taxa [262A|OL355150] and [282A|OL355152] are closely related and ancestral to *Kosakonia sp* and *Shigella sp*; taxa 282A|OL355152 is ancestral to a larger group consisting *Kosakonia sp*, *Shigella sp*, *Phytobacter sp*, *Metakosakonia sp*, *Atlantibacter sp*, *Escherichia sp*, *Pantoea sp*, *Enterobacter sp*, *Salmonella sp*. The taxon 283D|OL374164 is ancestral to *Serratia sp*, *Enterobacter sp* and 387C|OL375175 is ancestral to *Phytobacter sp*, *Metakosakonia sp*, *Atlantibacter sp*, *Escherichia sp*, *Enterobacter sp*, *Salmonella sp*. Although the taxa 262A|OL355150, 282A|OL355152, and 387C|OL375175 formed a single clade, BLASTn revealed that they are similar to *Enterobacter sp*. and *Atlantibacter sp*. Based on the Phylogenetic analysis shown in Fig 2 and S2 Fig (xxx) it can be stated that *Enterobacter hormaechei* might have some relation with oral cancer.

### Occurrence and distribution of microorganisms

*Staphylococcus* spp. were the most commonly occurring bacteria distributed among 60% of cancer patients, 61.7% of individuals who consumed intoxicating substances (consumers) and 74.1% non-consumers. *Bacillus* sp. were isolated from 53.33%, 59.70%, and 68.50% of people with oral cancer, consumers and non consumers respectively (Table 1). *Pseudomonas* species were mostly isolated from oral cancer patients and consumers. *Enterobacter* and *Candida* species were detected in the swab samples of oral cancer patients along others except *Rhodococcus*, *Acinetobacter* and *Klebsiella*. *Serratia* species occurred in 6.7%, 7.1%, and 7.9% of oral cancer patients, consumers and non consumers respectively (Table 1). The genus overlap between the three groups (oral cancer patients, consumers and non consumers) was 0.8–0.875 and the diversity index ranged between 0.95–1.00. *Pseudomonas* species were found widely distributed. *P*. *aeruginosa*, *S*. *marcescens*, *R*. *antrifimi*, *P*. *dendritiformis*, *B*. *cereus*, *S*. *carnosus*, *K*. *michiganensis*, *P*. *cedrina* were found to be widely associated with the oral cavity of individuals having a habit of consuming intoxicating substances. *E*. *hormaechei* was found to be associated with the oral cavity of oral cancer patients. *E*. *bacterium*, *S*. *nematodiphila*, *S*. *epidermidis* and other bacteria were found in the oral cavity of individuals who did not consume intoxicating substances. A phylogenetic tree was drawn using the sequences of isolated organisms from the three groups: non consumer (Non Intox), consumer (Intox) and oral cancer patients having history of consumption of intoxicating substances (Cancer). It is interesting to see how *P*. *aeruginosa* and *Pseudomonas sp*. (400A); *S*. *marcescens* and 283D Non Intox; *K*. *michiganensis* and *S*. *nematodiphila*; *P*. *cedrina* and *Pseudomonas* sp. (307A Non Intox) formed clade showing a close relationship (Fig 2). These relationships indicate the colonization of transitory pathogens or non-oral organisms overpowering the commensal groups.

**Table 1:** Distribution of microorganisms isolated from the oral cavity of consumer and non-consumers of intoxicating substances and cancer patients.

Microorganisms	Non-Consumer (Non Intox)	Consumer (Intox)	Cancer
*Staphylococcus sp*	66 (74.1%)	121 (61.7%)	9 (60%)
*Bacillus sp*	61(68.5%)	117 (59.7%)	8 (53.3%)
*Serratia sp*	7 (7.9%)	14 (7.1%)	1 (6.7%)
*Rhodococcus sp*	-	1 (.51%)	-
*Pseudomonas sp*	13 (14.6%)	39 (19.9%)	3 (20%)
*Acinetobacter sp*	1 (1.1%)	3 (1.5%)	-
*Enterobacter sp*	5 (5.6%)	8 (4.0%)	2 (13.3%)
*Klebsiella sp*	-	3 (1.5%)	-
*Candida sp*	3 (3.3%)	3 (1.5%)	2 (13.3%)

### Effect of intoxicating substances

**Fig 3:**
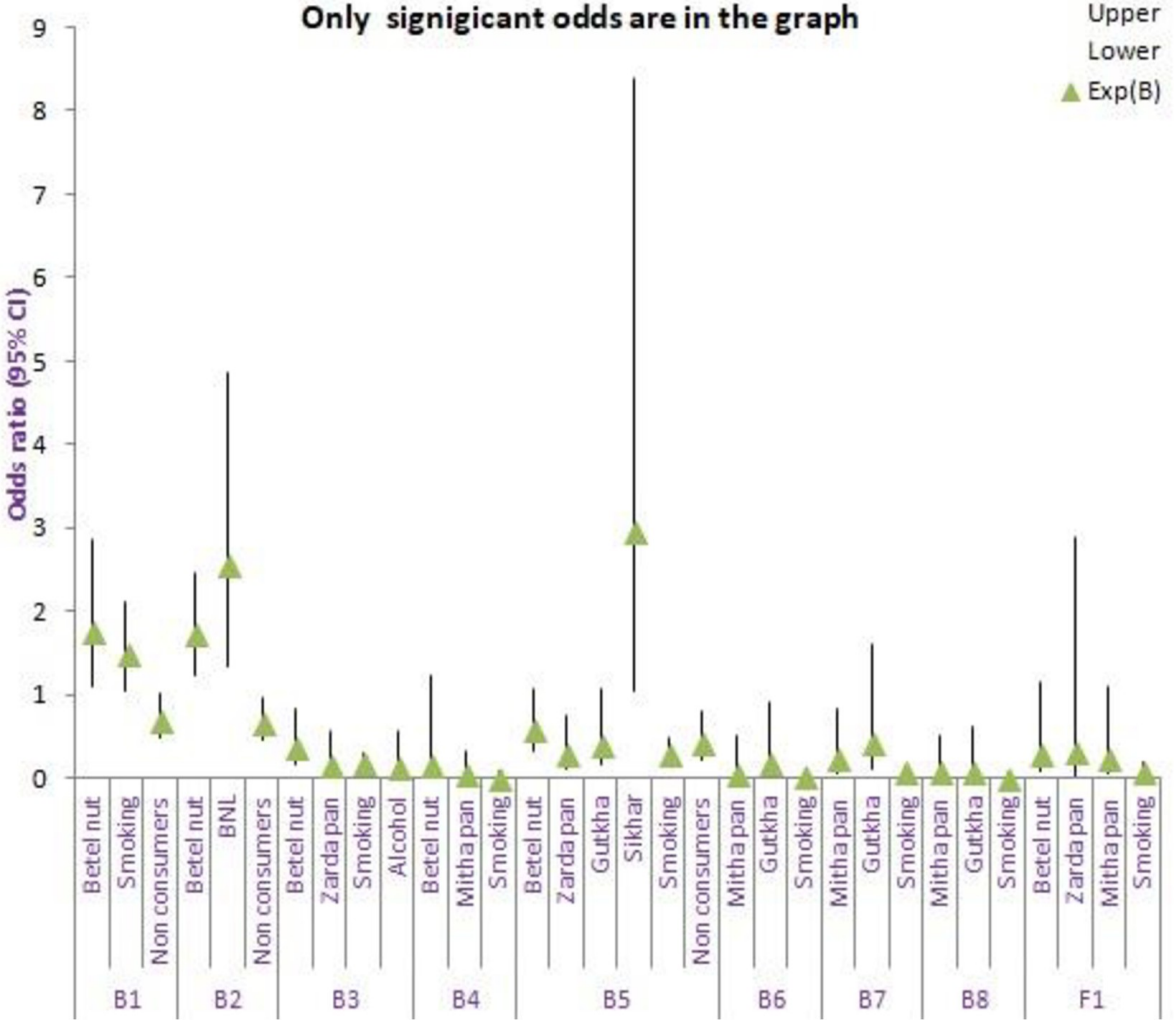
Risk (odds ratio) of incidence of microbes when exposed to intoxicating substances^1^. The x-axis represents occurrence of microbes on exposure (consumers) or non-exposure (non-consumers) to intoxicating substances, while the y-axis shows the odds ratio. The triangle symbol represents odds ration/Exp (B). The plot include odd ratio which significantly contributes to the regression. ^1^ B1 (*Staphylococcus*), B2(*Bacillus*), B3(*Serratia*), B4(*Rhodococcus*), B5(*Pseudomonas*), B6(*Acinetobacter*), B7(*Enterobacter*), B8(*Klebsiella*), Fl(*Candida*) CI-Confidence interval BNL-Betel nut with lime.

Exposure to various intoxicating substances affects the occurrence of microorganisms; the type of intoxicating substances determines the incidence of a specific organism. The risk of incidence of *Staphylococcus sp*. (B1) and *Bacillus sp*. (B2) was found to be 1.759 and 1.745 odds [EXP (B)] on exposure of oral cavity to betel nut. However, exposure to betel nut with lime increased the occurrence of *Bacillus sp*. (B2) (2.562). Exposure to ’sikhar’ (processed intoxicating substance) seemed to be inviting *Pseudomonas sp*. (B5) (2.963 odds). Exposure to tobacco smoking was also responsible for the incidence of *Staphylococcus sp*. The information on the risk of incidence of various organisms on exposure to different intoxicating substances is shown in Fig 3, S8 Table.

**Fig 4:**
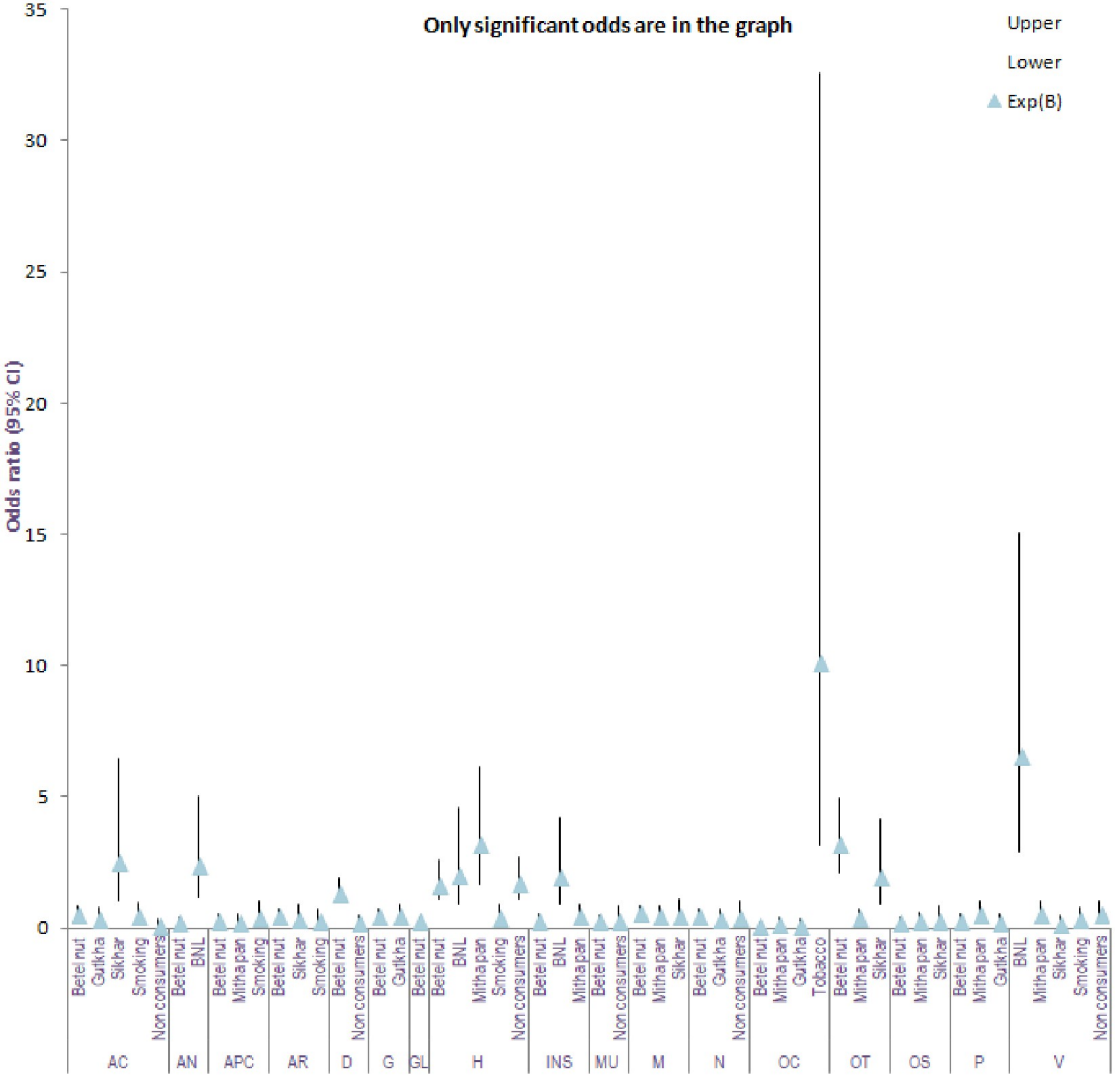
Risk (odds ratio) of health problems^1^ because of exposure (consumer), non-exposure (non-consumer) to intoxicating substances. The x-axis represents symptoms/diseases with exposure (consumer), non-exposure (non-consumer), to intoxicating substances, while the y-axis show the odds ratio. The triangle symbol represents odds ratio/Exp (B). The plot include odd ratio which significantly contributes to the regression. ^1^ Acute cellulites (AC), Anorexia (AN), Appetite condition (APC), Arthralgia (AR), Drowsiness (D), Gingivitis (G), Granuloma (GL), Headache (H), Insomnia (INS), Mouth ulceration (MU), Myalgia (M), Nausea (N), Oral cancer (OC), Oral thrush (OT), Osteomyelitis (OS), Periodontitis (P), Vision (V) Cl-Confidence interval BNL- Betel nut with lime.

All intoxicating substances posses certain risk for various health problems and diseases. The analysis indicated that exposure to chewing tobacco seemed to pose a higher risk for oral cancer (10.148 odds). Consumption of betel nut with lime showed responsibility for anorexia (2.387 odds), headache (2.025 odds), insomnia (1.982 odds) and also affects vision (6.608 odds). Exposure to sikhar was responsible for cellulitis (2.566 odds) and oral thrush (1.966 odds). Exposure to betel nut also increased oral thrush (3.226 odds). The information on the exposure to various intoxicating substances and corresponding health problem is shown in Fig 4, S9 Table.

**Fig 5:**
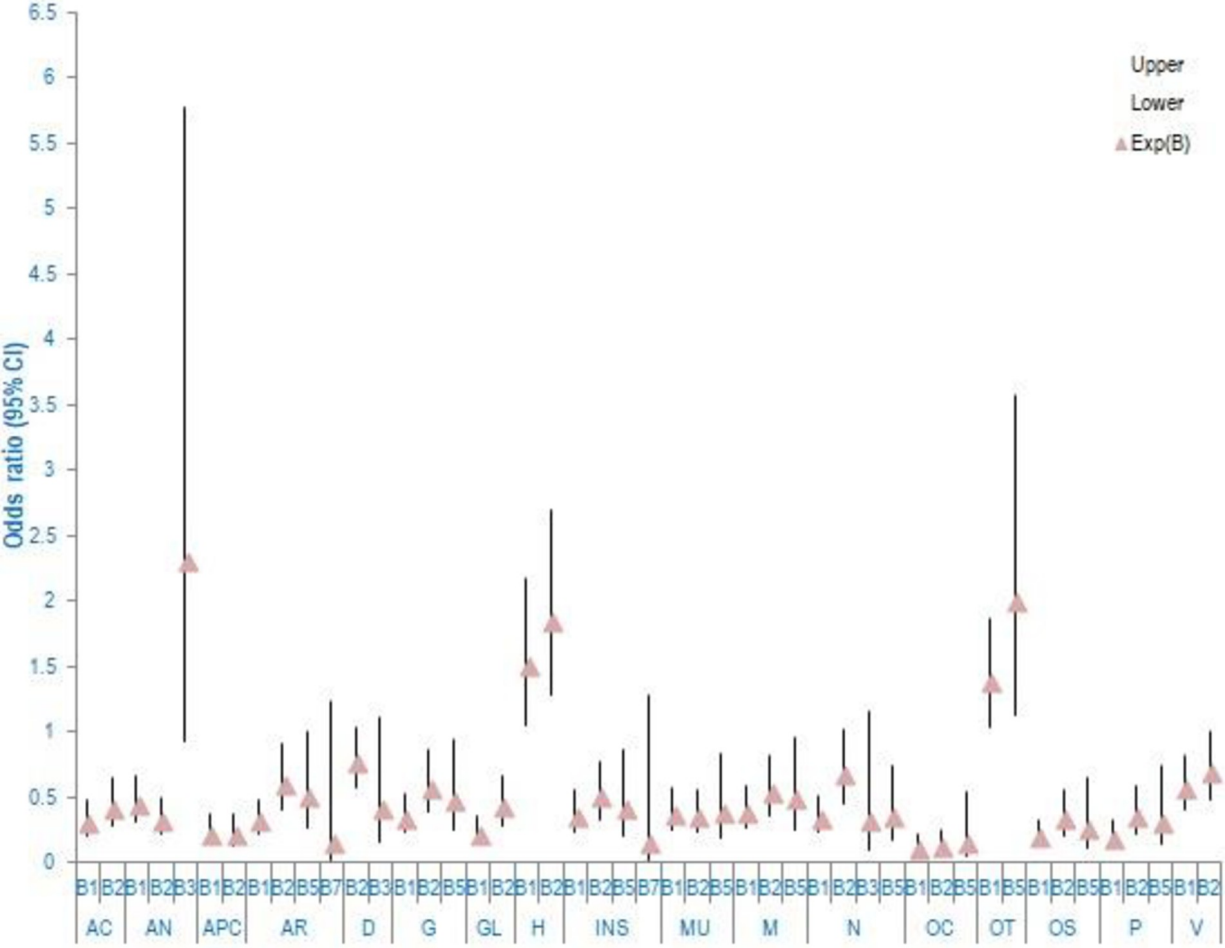
Risk of occurrence of health problems^1^ because of exposure to various microorganism^2^ irrespective of consumption or non-consumptions of intoxicating substances. The x-axis represents odds ratio/Exp(B). The plot include odd ratio which significantly contributes to the regression. ^1^Acute cellulites(AC), Anorexia (AN), Appetite condition(APC), Arthralgia (AR), Drowsiness (D), Gingivitis (G), Granuloma (GL), Headache (H), Insomnia (INS), Mouth ulceration(MU), Myalgia (M), Nausea (N), Oral cancer (OC), Oral thrush (OT), Osteomyelitis (OS), Periodontitis (P), Vision (V) ^2^B1 (*Staphylococcus*), B2(*Bacillus*), B3(*Serratia*), B4(*Rhodococcus*), B5(*Pseudomonas*), B6(*Acinetobacter*), B7(*Enterobacter*), B8(*Klebsiella*), FI (*Candida*) CI-Confidence interval.

The association of microorganisms with various health conditions showed that species of *Staphylococcus* sp (B1) were primarily associated with headache (1.513 odds), oral thrush (1.392 odds); *Bacillus* sp (B2) with headache (1.854 odds); *Serratia* sp (B3) with anorexia (2.306 odds) and *Pseudomonas* sp (B5) with oral thrush (2.003 odds). The information on the exposure to various microbes and corresponding health problems is shown in Fig 5, S10 Table.

## Discussion

Physiochemical changes in the oral cavity leads to changes in its microbiota [46]. Factors such as diet, oral hygiene, pH and immunity influence the cavity’s microbes [47, 48]. This study added intoxicating substances as other factors directly or indirectly affecting microbes of the cavity. The study reports *Pseudomonas sp*., *E*. *asburiae*, *S*. *nematodiphila*, and *S*. *epidermidis* in the oral cavity of non consumers, in contrast to the description of Jorn and colleagues, 2005 [32] stated that *Gemella*, *Granulicatella*, *Streptococcus*, and *Veillonella* species are normal bacterial flora of the oral cavity. This difference in findings may be attributed to the population groups, geographical locations and food habits. The transitory or colonial behavior of the microbes reported by us cannot be ruled out based on the statement “non-oral bacteria might colonize the oral ecosystem” [49–53]. Their ability for adaption may be attributed to their genetic makeup. Vidana et al. 2011 [53] showed *enterococci* of the oral cavity genetically differs from isolates of other human body locations.

The possible source of *Enterobacter asburiae* (the causative organism of rice bacterial blight in China [54], associated with cotton fever [55] may be rice as people of Assam also consume uncooked rice called ’pithaguri’ meaning rice flour. *S*. *epidermidis* is stated to be evolved not to cause disease but to maintain a benign relationship with its host [56].

Consumption of intoxicating substances creates conducive environment which attracts pathogens such as *P*. *aeruginosa*, *P*. *Cedrina*, *A*. *junii*, *S*. *marcescens*, *R*. *antrifimi*, *B*. *cereus*, *S*. *carnosus*, *and K*. *michiganensis*. The environmental changes may be attributed to arecoline which increases saliva secretion, stimulating sympathetic nerve and choline M receptor [57], nicotine, carbon monoxide, hydrogen cyanide, benzene, formaldehyde, phenol, polycyclic aromatic hydrocarbons and tobacco-specific nitrosamines [58] modulating pH. Reports point out that saccharolytic metabolism produces acids lowering the pH and allowing transitory acid tolerant and acidophiles including cariogenic bacteria to grow leading to dysbiosis [59]. Based on the tree clade, it can be stated that *P*. *aeruginosa* (141C Intox), *P*. *cedrina*, (387C)–unidentified organism, *Serratia marcescens* (168B Intox), *Bacillus* sp (OS1 Intox), *Klebsiella michiganensis* (162A Intox) are possible colonizers replacing the commensal microbes. Studies suggest that disturbances of "equilibrium" (due to medical treatments, biological changes and inadequate hygiene) between commensal bacteria and the host immune system could be the reason for transitory non-oral bacteria to colonize [60–62].

A direct relationship between microbes and health issues could not be drawn by the study however a risk of certain symptoms or diseases were evaluated. It shows that *Staphylococcus* sp (B1), *Bacillus* sp (B2), *Serratia* sp (B3), and *Pseudomonas* sp (B5) may be responsible for oral thrush, anorexia, and headache [63–67]. The presence of *E*. *hormaechei* in the cavity of oral cancer patients is a matter of concern since there were reports of outbreaks of *Enterobacter cloacae* at the cancer center in Tokyo, Japan [68]. *P*. *aeruginosa*, *Acinetobacter sp*., *S*. *marcescens*, *Rhodococcus sp*., *B*. *cereus*, and *K*. *michiganensis* are known pathogens, where nosocomial infections are caused by *P*. *aeruginosa* [69, 70], pneumonia by *Acinetobacter* [71] and *Rhodococcus* [72*]*, urinary tract infection by *S*. *marcescens* [73, 74], food poisoning, localized wound and eye infections by *B*. *cereus* [75]. *K*. *michiganensis* is an emerging multidrug-resistant human pathogen [76]. However, *P*. *cedrina*, a bio-pesticide against *Plutella xylostella* [77] and reported as antiproliferative against human cervical carcinoma cell lines Hela Lung A-549 (HBL-100) [78]. The present findings corroborate the bacterial characterization in betel quid chewer and non-chewer by Deepak et al. [34], poor oral hygiene and chronic periodontitis in betel nut chewers [79], microbial association with dysbiosis and increased risk of oral cancer [35].

The current study focused only on aerobes. Widening the scope may provide more information. Further analysis of the bacterial metabolites may reflect their associations with various health conditions, including anorexia, periodontitis, osteomyelitis and headache. A comparative molecular assessment between consumers and non-consumers may lead to understanding the favoritism regarding the colonization of pathogens.

## Conclusion

As per the study, intoxication significantly affects the normal microflora in the human oral cavity. The work has provided insight into how pathogenic or opportunistic pathogens can flourish by creating an environment that is conducive to their proliferation. Pathogens are probably restricting the growth of the normal flora. This study explains why healthy oral flora can be found in people who do not use intoxicants while pathogenic bacteria can be found in abundance in those who do use intoxicants. However the questions of how and why need more research because they are concerned with the conditions necessary for each microorganism to develop as well as the competitive dynamics between them. The presence *of E*. *hormaechei* in the cavity of oral cancer patients needs further investigation. Studies on microbiomes, molecular and biochemical changes may help to further explain the nature of illnesses brought on by pathogens or toxins opening the way to efficient diagnostic and treatment approaches that will eventually aid in the development of personalized medicine.

## Supporting information

S1 Table List of the participants and their gender. (PDF)

S2 Table Record of the consumption of different intoxicating substances, based on participant’s statements. (PDF)

S3 Table Record on the pattern of consumption (i.e., number of time consumption, daily/weekly/ monthly) and the total duration of consumption of the intoxicating substances along with their oral hygiene. (PDF)

S4 Table Record of health issues reported by the participants. (PDF)

S5 Table Record on microbes isolated from swab. (PDF)

S6 Table Descriptive statistics of the samples/participants of different age groups, consumers and non-consumers of intoxicating substances. (PDF)

S7 Table Oral cavity cleanliness record of the participants (male and female) grouped by age. (PDF)

S8 Table Risk of incidence of microbes when exposed to intoxicating substances. (PDF)

S9 Table Risk of health issues when exposed to intoxicating substances. (PDF)

S10 Table Risk of health issues when exposed to microorganisms. (PDF)

S1 Fig Images of microorganisms under microscope. (TIFF)

S2 Fig Phylogenetic trees developed for identification of the isolated microbes. (TIFF)

## References

[1] Gupta PC, Warnakulasuriya S. Global epidemiology of areca nut usage. Addict Biol. 2002;7(1):77–83. doi: 10.1080/13556210020091437 11900626

[2] Nelson BS, Heischober B. Betel nut: A common drug used by naturalized citizens from India, Far East Asia, and the South Pacific Islands. Ann Emerg Med. 1999;34(2):238–43. doi: 10.1016/s0196-0644(99)70239-8 10424931

[3] Zhang P, Sari EF, McCullough MJ, Cirillo N. Metabolomic Profile of Indonesian Betel Quids. Biomolecules. 2022 Oct 1;12(10). doi: 10.3390/biom12101469 36291678 PMC9599835

[4] Marsh PD. Role of the oral microflora in health. Microb Ecol Health Dis. 2000;12(3):130–7. https://www.tandfonline.com

[5] Thun MJ, Jane Henley S. Tobacco. Cancer Epidemiol Prev. 2006 Oct 12; https://academic.oup.com/book/40097/chapter/341247984

[6] Marshall JR, Freudenheim J. Alcohol. Cancer Epidemiol Prev. 2006 Oct 12; https://academic.oup.com/book/40097/chapter/341248207

[7] Homann N, Jousimies-Somer H, Jokelainen K, Heine R, Salaspuro M. High acetaldehyde levels in saliva after ethanol consumption: Methodological aspects and pathogenetic implications intestine, kidney and bone marrow (15–17) acetaldehyde can be formed from ethanol via microbial ADH. Carcinogenesis. 1997;18(9):1739–43. 9328169 10.1093/carcin/18.9.1739

[8] Verna L, Whysner J, Williams GM. N-Nitrosodiethylamine mechanistic data and risk assessment: Bioactivation, DNA-adduct formation, mutagenicity, and tumor initiation. Pharmacol Ther. 1996;71(1–2):57–81. doi: 10.1016/0163-7258(96)00062-9 8910949

[9] Ramôa CP, Eissenberg T, Sahingur SE. Increasing popularity of waterpipe tobacco smoking and electronic cigarette use: Implications for oral healthcare. J Periodontal Res. 2017 Oct 1;52(5):813–23. https://pubmed.ncbi.nlm.nih.gov/28393367/ doi: 10.1111/jre.12458 28393367 PMC5585021

[10] Vaiyapuri S, Kaiser W, Bicknell A, Gibbins J, Lowry P. Smoke-induced nausea; mediated by the release of the lung tachykinin, endokinin, into the circulation? Endocr Abstr. 2017 Oct 20;50.https://www.endocrine-abstracts.org/ea/0050/ea0050p270

[11] Baboni FB, Barp D, de Azevedo Izidoro ACS, Samaranayake LP, Rosa EAR. Enhancement of Candida albicans virulence after exposition to cigarette mainstream smoke. Mycopathologia. 2009 Oct;168(5):227–35. https://pubmed.ncbi.nlm.nih.gov/19544010/ doi: 10.1007/s11046-009-9217-5 19544010

[12] Semlali A, Killer K, Alanazi H, Chmielewski W, Rouabhia M. Cigarette smoke condensate increases C. albicans adhesion, growth, biofilm formation, and EAP1, HWP1 and SAP2 gene expression. BMC Microbiol. 2014 Mar 12;14(1):61. doi: 10.1186/1471-2180-14-61 24618025 PMC3995653

[13] ArulJothi K N, Irusappan S, Amarnath G, Chandrasekaran S, K SAB, Harishankar M, et al. Pyogenic granuloma of buccal mucosa: An original article. IJSR—Int J Sci Res. 2016 Oct 1;Volume 5 Issue 9(2):63–5. https://www.worldwidejournals.com/international-journal-of-scientific-research-(IJSR)

[14] McKinney R, Olmo H. Pathologic Manifestations Of Smokeless Tobacco. StatPearls. 2022 Nov 29; https://www.ncbi.nlm.nih.gov/books/NBK573058/34424631

[15] Chaudhuri S, Dey S, Bajpai RC. Prevalence of oral ulcers and its association with addictions in rural population of western Uttar Pradesh and eastern Rajasthan. J oral Biol craniofacial Res. 2016 Sep 1;6(3):179–86. https://pubmed.ncbi.nlm.nih.gov/27761381/ doi: 10.1016/j.jobcr.2016.04.003 PMC5064978 27761381

[16] Lewis SD, Peter GS, Gómez-Marín O, Bisno AL. Risk factors for recurrentlower extremity cellulitis in a U.S. Veterans Medical Center population. Am J Med Sci. 2006 Dec;332(6):304–7. https://pubmed.ncbi.nlm.nih.gov/17170620/ doi: 10.1097/00000441-200612000-00002 17170620

[17] Kale M, Garde J, Deshmukh V, Kulkarni A. Osteomyelitis of Mandible in Chronic Alcoholic—A Post Extraction Complication. J Dent Allied Sci. 2012;1(1):32. https://www.researchgate.net/publication/290463266

[18] Han SY, Chang Y, Kim Y, Choi CY, Ryu S. A Dose-Response Relationship of Alcohol Consumption with Risk of Visual Impairment in Korean Adults: The Kangbuk Samsung Health Study. Nutrients. 2022 Feb 1;14(4). https://pubmed.ncbi.nlm.nih.gov/35215441/ doi: 10.3390/nu14040791 PMC8875794 35215441

[19] Brasil A, Castro AJO, Martins ICVS, Lacerda EMCB, Souza GS, Herculano AM, et al. Colour Vision Impairment in Young Alcohol Consumers. PLoS One. 2015 Oct 14;10(10):e0140169. https://journals.plos.org doi: 10.1371/journal.pone.0140169 26465148 PMC4605530

[20] Erwin CW, Wiener EL, Linnoila MI, Truscott TR. Alcohol-induced drowsiness and vigilance performance. J Stud Alcohol. 1978;39(3):505–16. https://pubmed.ncbi.nlm.nih.gov/651360/ doi: 10.15288/jsa.1978.39.505 651360

[21] Urbano-Márquez A, Fernández-Solà J. Effects of alcohol on skeletal and cardiac muscle. Muscle and Nerve. 2004 Dec;30(6):689–707. doi: 10.1002/mus.20168 15490485

[22] Lilenfeld LR, Kaye WH. The Link Between Alcoholism and Eating Disorders. Alcohol Health Res World.1996;20(2):94. 31798142 PMC6876496

[23] Giri S, Idle JR, Chen C, Zabriskie TM, Krausz KW, Gonzalez FJ. A Metabolomic Approach to the Metabolism of the Areca Nut Alkaloids Arecoline and Aracaidine in the Mouse. Chem Res Toxicol. 2006 Jun;19(6):818. 16780361 10.1021/tx0600402 PMC1482804

[24] Chu N-S. Effects of betel chewing on the central and autonomic nervous systems. J Biomed Sci. 2001 May;8(3):229–36. doi: 10.1007/BF02256596 11385294

[25] Giri DK, Kundapur P, Bhat KM, Maharjan IK. Betel Nut Chewing Associated With Severe Periodontitis. Heal Renaiss. 2015 Jan 28;12(1):57–60. https://www.researchgate.net/publication/276264145

[26] Stayner L, Steenland K, Dosemeci M, Hertz-Picciotto I. Attenuation of exposure-response curves in occupational cohort studies at high exposure levels. Scand J Work Environ Heal. 2003;29(4):317–24. doi: 10.5271/sjweh.737 12934726

[27] Akhter R, Hassan NMM, Aida J, Takinami S, Morita M. Relationship between betel quid additives and established periodontitis among Bangladeshi subjects. J Clin Periodontol. 2008 Jan;35(1):9–15. doi: 10.1111/j.1600-051X.2007.01164.x 18021263

[28] Amarasena N, Ekanayaka ANI, Herath L, Miyazaki H. Association between smoking, betel chewing and gingival bleeding in rural Sri Lanka. J Clin Periodontol. 2003 May;30(5):403–8. doi: 10.1034/j.1600-051x.2003.20010.x 12716331

[29] Strickland SS, Veena G V., Houghton PJ, Stanford SC, Kurpad A V. Areca nut, energy metabolism and hunger in Asian men. Ann Hum Biol. 2003 Jan;30(1):26–52. https://pubmed.ncbi.nlm.nih.gov/12519653/ doi: 10.1080/03014460210157448 12519653

[30] S. M. K A, M S., Hegde S, R K.S. Effect of chewing gutkha on oral hygiene, gingival and periodontal status. J Oral Heal Res. 2012 Jul 1;3(3):26–32. https://go.gale.com

[31] Bik EM, Long CD, Armitage GC, Loomer P, Emerson J, Mongodin EF, et al. Bacterial diversity in the oral cavity of ten healthy individuals. ISME J. 2010 Aug;4(8):962. 20336157 10.1038/ismej.2010.30 PMC2941673

[32] Aas JA, Paster BJ, Stokes LN, Olsen I, Dewhirst FE. Defining the normal bacterial flora of the oral cavity. J Clin Microbiol. 2005 Nov;43(11):5721–32. doi: 10.1128/JCM.43.11.5721-5732.2005 16272510 PMC1287824

[33] Vyas D, Galra B, Dagli R, Gupta PP, Vyas A, Parekh D. Characterization of bacteria in betel quiz chewers and non -chewers and their associated oral health status. J Res Dent. 2017 May 4;4(4):128.

[34] Parmar G, Sangwan P, Vashi P, Kulkarni P, Kumar S. Effect of chewing a mixture of areca nut and tobacco on periodontal tissues and oral hygiene status. J Oral Sci. 2008 Mar;50(1):57–62. https://pubmed.ncbi.nlm.nih.gov/18403885/ doi: 10.2334/josnusd.50.57 18403885

[35] Ivanov II, Littman DR. Modulation of immune homeostasis by commensal bacteria. Curr Opin Microbiol. 2011 Feb;14(1):106–14. https://pubmed.ncbi.nlm.nih.gov/21215684/ doi: 10.1016/j.mib.2010.12.003 21215684 PMC3123735

[36] Jiang Y, Zhou X, Cheng L, Li M. The Impact of Smoking on Subgingival Microflora: From Periodontal Health to Disease. Front Microbiol. 2020 Jan 29;11. https://pubmed.ncbi.nlm.nih.gov/32063898/ doi: 10.3389/fmicb.2020.00066 PMC7000377 32063898

[37] van Winkelhoff AJ, Bosch-Tijhof CJ, Winkel EG, van der Reijden WA. Smoking affects the subgingival microflora in periodontitis. J Periodontol. 2001 May;72(5):666–71. https://pubmed.ncbi.nlm.nih.gov/11394403/ doi: 10.1902/jop.2001.72.5.666 11394403

[38] Kubota M, Tanno-Nakanishi M, Yamada S, Okuda K, Ishihara K. Effect of smoking on subgingival microflora of patients with periodontitis in Japan. BMC Oral Health. 2011 Jan 5;11(1). https://pubmed.ncbi.nlm.nih.gov/21208407/ doi: 10.1186/1472-6831-11-1 PMC3020163 21208407

[39] Könönen E, Asikainen S, Jousimies-Somer H. The early colonization of gram-negative anaerobic bacteria in edentulous infants. Oral Microbiol Immunol. 1992; 7(1):28–31. https://pubmed.ncbi.nlm.nih.gov/1528621/ doi: 10.1111/j.1399-302x.1992.tb00016.x 1528621

[40] Zhao H, Chu M, Huang Z, Yang X, Ran S, Hu B, et al. Variations in oral microbiota associated with oral cancer. Sci Rep. 2017 Dec 1;7(1). https://pubmed.ncbi.nlm.nih.gov/28924229/ doi: 10.1038/s41598-017-11779-9 PMC5603520 28924229

[41] Berger G, Bitterman R, Azzam ZS. The Human Microbiota: The Rise of an “Empire”. Rambam Maimonides Med J. 2015 Apr 29;6(2):e0018. 25973270 10.5041/RMMJ.10202 PMC4422457

[42] Lane D.J. (1991) 16S/23S rRNA Sequencing. In Stackebrandt E. and Goodfellow M., Eds., Nucleic Acid Techniques in Bacterial Systematic, John Wiley and Sons, New York, 115–175. https://www.scirp.org/1870033

[43] http://technelysium.com.au/wp/chromas/.

[44] Huang X, Madan A. CAP3: A DNA sequence assembly program. Genome Res. 1999 Sep;9(9):868–77. https://pubmed.ncbi.nlm.nih.gov/10508846/ doi: 10.1101/gr.9.9.868 10508846 PMC310812

[45] BLAST: Basic Local Alignment Search Tool. https://blast.ncbi.nlm.nih.gov/Blast.cgi

[46] Zaatout N. Presence of non-oral bacteria in the oral cavity. Arch Microbiol. 2021 Aug 1;203(6):2747–60. https://pubmed.ncbi.nlm.nih.gov/33791834/ doi: 10.1007/s00203-021-02300-y 33791834 PMC8012020

[47] Atlas RM, Bartha R. Microbial ecology: fundamentals and applications. 1998;694.

[48] Sarmah R, Khan RA, Devi KR. Microbes in human oral cavity: a review. Rev Med Microbiol. 2021 Apr 7;32(2):75–82. https://www.researcher-app.com/paper/7358569

[49] Souto R, Colombo APV. Prevalence of Enterococcus faecalis in subgingival biofilm and saliva of subjects with chronic periodontal infection. Arch Oral Biol. 2008 Feb;53(2):155–60. https://pubmed.ncbi.nlm.nih.gov/17897617/ doi: 10.1016/j.archoralbio.2007.08.004 17897617

[50] Gonçalves L de S, Soares Ferreira SM, Souza CO, Souto R, Colombo AP. Clinical and microbiological profiles of human immunodeficiency virus (HIV)-seropositive Brazilians undergoing highly active antiretroviral therapy and HIV-seronegative Brazilians with chronic periodontitis. J Periodontol. 2007 Jan;78(1):87–96. https://pubmed.ncbi.nlm.nih.gov/17199544/ doi: 10.1902/jop.2007.060040 17199544

[51] Da Silva-Boghossian CMI, Do Souto RM, Luiz RR, Colombo APV. Association of red complex, A. actinomycetemcomitans and non-oral bacteria with periodontal diseases. Arch Oral Biol. 2011 Sep;56(9):899–906. https://pubmed.ncbi.nlm.nih.gov/21397893/ doi: 10.1016/j.archoralbio.2011.02.009 21397893

[52] Silva-Boghossian CM, Neves AB, Resende FAR, Colombo APV. Suppuration-associated bacteria in patients with chronic and aggressive periodontitis. J Periodontol. 2013 Sep;84(9):e9–16. https://pubmed.ncbi.nlm.nih.gov/23327648/ doi: 10.1902/jop.2013.120639 23327648

[53] Vidana R, Sullivan A, Billström H, Ahlquist M, Lund B. Enterococcus faecalis infection in root canals—host-derived or exogenous source? Lett Appl Microbiol. 2011 Feb;52(2):109–15. https://pubmed.ncbi.nlm.nih.gov/21155997/ doi: 10.1111/j.1472-765X.2010.02972.x 21155997

[54] Xue Y, Hu M, Chen S, Hu A, Li S, Han H, et al. Enterobacter asburiae and Pantoea ananatis Causing Rice Bacterial Blight in China. Plant Dis. 2021 Aug 1;105(8). https://pubmed.ncbi.nlm.nih.gov/33342235/ doi: 10.1094/PDIS-10-20-2292-RE 33342235

[55] Francis MJ, Chin J, Lomiguen CM, Glaser A. Cotton fever resulting in Enterobacter asburiae endocarditis. IDCases. 2020 Jan 1;19. /pmc/articles/PMC6938846/10.1016/j.idcr.2019.e00688 PMC6938846 31908949

[56] Otto M. Staphylococcus epidermidis–the “accidental” pathogen. Nat Rev Microbiol. 2009;7(8):555. doi: 10.1038/nrmicro2182 19609257 PMC2807625

[57] Chen X, He Y, Deng Y. Chemical Composition, Pharmacological, and Toxicological Effects of Betel Nut. Evid Based Complement Alternat Med. 2021;2021. doi: 10.1155/2021/1808081 34457017 PMC8387188

[58] Engstrom PF, Clapper ML, Schnoll RA. Physiochemical Composition of Tobacco Smoke. 2003; https://www.ncbi.nlm.nih.gov/books/NBK13173/

[59] Bradshaw DJ, Marsh PD. Analysis of pH-driven disruption of oral microbial communities in vitro. Caries Res. 1998;32(6):456–62. https://pubmed.ncbi.nlm.nih.gov/9745120/ doi: 10.1159/000016487 9745120

[60] Handal T, Caugant DA, Olsen I. Antibiotic Resistance in Bacteria Isolated from Subgingival Plaque in a Norwegian Population with Refractory Marginal Periodontitis. Antimicrob Agents Chemother. 2003 Apr 1;47(4):1443. doi: 10.1128/AAC.47.4.1443-1446.2003 12654689 PMC152519

[61] Dahlén G. Bacterial infections of the oral mucosa. Periodontol 2000. 2009 Feb;49(1):13–38. https://pubmed.ncbi.nlm.nih.gov/19152524/ doi: 10.1111/j.1600-0757.2008.00295.x 19152524

[62] Tada A, Hanada N. Opportunistic respiratory pathogens in the oral cavity of the elderly. FEMS Immunol Med Microbiol. 2010 Oct;60(1):1–17. https://pubmed.ncbi.nlm.nih.gov/20579096/ doi: 10.1111/j.1574-695X.2010.00709.x 20579096

[63] Gallaher C, Norman J, Singh A, Sanderson F. Community-acquired Pseudomonas aeruginosa meningitis. BMJ Case Rep. 2017;2017. https://pubmed.ncbi.nlm.nih.gov/29054951/ doi: 10.1136/bcr-2017-221839 PMC5665197 29054951

[64] Kavanagh N, Ryan EJ, Widaa A, Sexton G, Fennell J, O’Rourke S, et al. Staphylococcal Osteomyelitis: Disease Progression, Treatment Challenges, and Future Directions. Clin Microbiol Rev. 2018 Apr 1;31(2). doi: 10.1128/CMR.00084-17 29444953 PMC5967688

[65] Langhans W. Anorexia of infection: current prospects. Nutrition. 2000;16(10):996–1005. https://pubmed.ncbi.nlm.nih.gov/11054606/ doi: 10.1016/s0899-9007(00)00421-4 11054606

[66] Nagarjunakonda S, Amalakanti S, Siddabathuni A, Pantagada N. Bacterial flora of the migraine nose: Pilot case–control study of nasal bacteria in migraine headache. J Med Sci. 2017 Sep 1;37(5):186. https://www.jmedscindmc.com

[67] Bascones-Martínez A, Figuero-Ruiz E. Periodontal diseases as bacterial infection. Med Oral Patol Oral Cir Bucal. 2004;9 Suppl. https://pubmed.ncbi.nlm.nih.gov/15580140/ doi: 10.4321/s1699-65852005000300002 15580140

[68] Harada S, Aoki K, Ohkushi D, Okamoto K, Takehana K, Akatsuchi T, et al. Institutional outbreak involving multiple clades of IMP-producing Enterobacter cloacae complex sequence type 78 at a cancer center in Tokyo, Japan. BMC Infect Dis. 2021 Dec 1;21(1). https://pubmed.ncbi.nlm.nih.gov/33752612/10.1186/s12879-021-05952-9 PMC7983292 33752612

[69] Kollef MH, Torres A, Shorr AF, Martin-Loeches I, Micek ST. Nosocomial Infection. Crit Care Med. 2021 Feb 1;49(2):169–87. https://pubmed.ncbi.nlm.nih.gov/33438970/ doi: 10.1097/CCM.0000000000004783 33438970

[70] Dasgupta S, Das S, Chawan NS, Hazra A. Nosocomial infections in the intensive care unit: Incidence, risk factors, outcome and associated pathogens in a public tertiary teaching hospital of Eastern India. Indian J Crit Care Med. 2015 Jan 1;19(1):14–20. https://pubmed.ncbi.nlm.nih.gov/25624645/ doi: 10.4103/0972-5229.148633 25624645 PMC4296405

[71] Hartzell JD, Kim AS, Kortepeter MG, Moran KA. Acinetobacter Pneumonia: A Review. Medscape Gen Med. 2007;9(3):4. 18092011 PMC2100077

[72] Majidzadeh M, Fatahi-Bafghi M. Current taxonomy of Rhodococcus species and their role in infections. Eur J Clin Microbiol Infect Dis. 2018 Nov 1;37(11):2045–62. https://pubmed.ncbi.nlm.nih.gov/30159693/ doi: 10.1007/s10096-018-3364-x 30159693

[73] Hejazi A, Falkiner FR. Serratia marcescens. J Med Microbiol. 1997;46(11):903–12. https://pubmed.ncbi.nlm.nih.gov/9368530/ doi: 10.1099/00222615-46-11-903 9368530

[74] Khanna A, Khanna M, Aggarwal A. Serratia Marcescens- A Rare Opportunistic Nosocomial Pathogen and Measures to Limit its Spread in Hospitalized Patients. J Clin Diagn Res. 2013 Feb 1 7(2):243. doi: 10.7860/JCDR/2013/5010.2737 23543704 PMC3592283

[75] Ehling-Schulz M, Lereclus D, Koehler TM. The Bacillus cereus Group: Bacillus Species with Pathogenic Potential. Microbiol Spectr. 2019 May 31;7(3). https://pubmed.ncbi.nlm.nih.gov/31111815/ doi: 10.1128/microbiolspec.GPP3-0032-2018 PMC6530592 31111815

[76] Seiffert SN, Wüthrich D, Gerth Y, Egli A, Kohler P, Nolte O. First clinical case of KPC-3-producing Klebsiella michiganensis in Europe. New microbes new Infect. 2019 May 1;29. https://pubmed.ncbi.nlm.nih.gov/30949345/ doi: 10.1016/j.nmni.2019.100516 PMC6428935 30949345

[77] Liu FH, Lin XL, Kang ZW, Tian HG, Liu TX. Isolation and characterization of Pseudomonas cedrina infecting Plutella xylostella (Lepidoptera: Plutellidae). Arch Insect Biochem Physiol. 2019 Nov 1;102(3). https://pubmed.ncbi.nlm.nih.gov/31612553/ doi: 10.1002/arch.21593 31612553

[78] Sánchez-Tafolla L, Padrón JM, Mendoza G, Luna-Rodríguez M, Fernández JJ, Norte M, et al. Antiproliferative activity of biomass extract from Pseudomonas cedrina. Electron J Biotechnol. 2019 Jul 1;40:40–4.

[79] Chatrchaiwiwatana S. Dental Caries and Periodontitis Associated with Betel Quid Chewing: Analysis of Two Data Sets. J Med Assoc Thai. 2006;89(7):1004–15. 16881434

