# Supplementary material for: Bacteria in the oral cavity of individuals consuming intoxicating substances
Source: PLoS One. 2023 May 26;18(5):e0285753. doi: 10.1371/journal.pone.0285753 (PMC10218728; doi:10.1371/journal.pone.0285753)
Supplement: S1 Table — (PDF) [file pone.0285753.s001.pdf]

S1-Table: List of the participants and their gender.											
Participant ID	Male (1) /Female (2)	Participant ID	Male (1) /Female (2)	Participant ID	Male (1) /Female (2)	Participant ID	Male (1) /Female (2)	Participant ID	Male (1) /Female (2)	Participant ID	Male (1) /Female (2)
101	1	128	2	155	1	182	1	209	2	236	1
102	1	129	1	156	1	183	2	210	2	237	1
103	2	130	2	157	2	184	2	211	2	238	1
104	1	131	1	158	2	185	2	212	1	239	1
105	2	132	1	159	2	186	1	213	2	240	2
106	2	133	1	160	1	187	1	214	1	241	1
107	1	134	1	161	1	188	1	215	2	242	1
108	2	135	1	162	1	189	1	216	2	243	1
109	1	136	1	163	2	190	1	217	2	244	1
110	2	137	1	164	2	191	1	218	2	245	1
111	2	138	1	165	2	192	1	219	2	246	1
112	1	139	1	166	2	193	2	220	2	247	1
113	2	140	2	167	2	194	1	221	1	248	1
114	1	141	1	168	2	195	1	222	2	249	1
115	1	142	1	169	2	196	1	223	2	250	1
116	1	143	2	170	2	197	2	224	2	251	1
117	1	144	2	171	2	198	1	225	2	252	2
118	1	145	1	172	2	199	1	226	2	253	2
119	1	146	2	173	2	200	1	227	2	254	2
120	2	147	2	174	2	201	2	228	1	255	1
121	1	148	1	175	2	202	1	229	2	256	1
122	1	149	2	176	2	203	2	230	1	257	2
123	1	150	1	177	2	204	1	231	1	258	1
124	2	151	1	178	2	205	2	232	1	259	2
125	1	152	1	179	1	206	1	233	1	260	1
126	2	153	1	180	1	207	2	234	1	261	2
127	2	154	1	181	1	208	1	235	1	262	2

Participant ID	Male (1) /Female (2) Participant ID		Male (1) /Female (2)	Participant ID	Male (1) /Female (2)	Participant ID	Male (1) /Female (2)	Participant ID	Male (1) /Female (2)
263	1	291	1	319	2	347	2	375	2
264	1	292	1	320	1	348	2	376	1
265	1	293	1	321	1	349	2	377	1
266	1	294	1	322	2	350	2	378	1
267	1	295	1	323	2	351	2	379	2
268	1	296	2	324	2	352	2	380	1
269	1	297	1	325	2	353	2	381	2
270	1	298	1	326	1	354	2	382	1
271	1	299	1	327	2	355	2	383	2
272	1	300	1	328	2	356	2	384	2
273	1	301	1	329	1	357	1	385	2
274	1	302	1	330	1	358	2	386	1
275	1	303	1	331	2	359	1	387	1
276	1	304	1	332	2	360	1	388	1
277	1	305	1	333	2	361	1	389	1
278	1	306	1	334	2	362	1	390	1
279	1	307	1	335	2	363	1	391	1
280	1	308	2	336	2	364	1	392	1
281	1	309	2	337	1	365	2	393	1
282	1	310	1	338	1	366	2	394	1
283	1	311	1	339	1	367	1	395	1
284	1	312	1	340	1	368	2	396	1
285	1	313	1	341	2	369	1	397	1
286	1	314	1	342	2	370	2	398	1
287	1	315	1	343	2	371	1	399	1
288	1	316	1	344	1	372	2	400	1
289	1	317	1	345	1	373	2		
290	2	318	2	346	1	374	2		

	No. of Participants
Male:	183
Female:	117
**TOTAL**	**300**

The personal information of the participants such as location, age, name, address is not shown in the table. Three hundred individuals containing 183 male and 117 females were interviewed and oral swab were collected.
