# Supplementary material for: Bacteria in the oral cavity of individuals consuming intoxicating substances
Source: PLoS One. 2023 May 26;18(5):e0285753. doi: 10.1371/journal.pone.0285753 (PMC10218728; doi:10.1371/journal.pone.0285753)
Supplement: S2 Table — (PDF) [file pone.0285753.s002.pdf]

S2-Table: Record of the consumption of different intoxicating substances, based on participant's statements.

PARTICIPANT ID	(1 = YES, 0 = NO)								
	BETEL NUT	BETEL NUT WITH LIME (Process include extra use of lime)	ZARDA PAN (Dried Betel nut)	MITHA PAN (Dried Betel nut)	GUTKHA (Processed Betel nut)	SIKHAR (Processed Betel nut)	SMOKING TOBACCO	ALCOHOL	CHEWING TOBACCO
101	1	1	1	0	0	0	1	0	0
102	1	1	0	1	0	0	0	0	0
103	0	0	0	0	0	0	0	0	1
104	1	0	0	1	0	0	0	0	0
105	1	0	0	0	0	0	0	0	0
106	0	0	0	0	0	0	0	0	0
107	0	0	0	0	0	0	0	0	0
108	0	0	0	0	0	0	0	0	0
109	0	0	1	1	0	0	1	0	0
110	1	0	0	1	1	1	1	1	0
111	1	0	0	1	0	0	0	0	0
112	1	0	1	0	0	0	1	0	0
113	1	0	0	1	0	0	0	0	0
114	1	1	1	0	1	1	0	1	0
115	1	0	0	1	0	0	1	0	0
116	1	1	0	0	0	0	1	0	0
117	0	0	0	1	0	0	1	0	0
118	1	1	1	1	1	1	1	1	0
119	1	0	1	1	0	0	0	0	0
120	1	0	0	1	0	0	0	0	0
121	0	0	0	1	0	1	1	1	0
122	0	0	0	0	0	0	0	0	0
123	1	1	1	1	1	1	1	1	0
124	0	0	0	0	0	0	0	1	0
125	0	0	0	0	0	0	0	0	0
126	1	0	0	0	0	0	0	0	0
127	1	0	0	1	0	0	0	0	0

128	0	0	0	1	0	0	0	0	0
129	0	0	1	1	0	0	1	0	0
130	0	0	0	0	0	0	0	0	0
131	0	0	0	1	0	0	0	0	0
132	1	1	1	1	1	1	1	1	1
133	1	0	0	1	1	1	1	1	0
134	0	0	0	0	0	0	0	0	0
135	1	1	1	0	1	1	0	0	0
136	0	0	0	0	0	0	0	1	0
137	0	0	0	0	0	0	0	0	0
138	1	0	0	1	0	0	0	1	0
139	1	0	1	1	0	1	1	1	0
140	0	0	0	0	0	0	0	0	0
141	0	0	0	0	0	0	1	0	0
142	1	0	1	1	1	0	1	0	0
143	1	1	0	1	1	0	0	0	0
144	1	1	0	1	0	0	0	0	0
145	0	0	0	0	0	0	0	0	0
146	1	1	1	1	0	1	0	1	0
147	1	1	0	0	0	1	0	0	1
148	1	1	0	1	1	0	0	0	0
149	0	0	0	0	0	0	0	0	0
150	1	0	1	0	1	0	1	0	0
151	1	0	0	1	1	0	1	1	0
152	0	0	0	1	0	0	1	1	0
153	0	0	0	1	0	0	0	0	0
154	1	1	0	1	0	0	0	0	0
155	1	1	1	0	1	0	0	0	0
156	0	0	1	0	1	1	0	0	0
157	0	0	0	1	0	0	0	0	0
158	0	0	0	1	0	0	0	0	0
159	0	0	0	0	1	0	0	0	0

160	0	0	0	0	1	0	0	0	0
161	1	1	1	1	0	0	0	1	0
162	0	0	0	0	1	0	0	1	0
163	1	1	1	0	0	0	0	1	0
164	1	1	0	0	0	0	0	0	0
165	1	1	0	0	0	0	0	0	0
166	1	0	0	0	0	0	0	0	0
167	1	1	0	0	0	0	0	1	0
168	1	1	0	0	0	0	0	0	0
169	1	1	0	0	0	0	0	0	0
170	1	1	1	0	0	0	0	0	0
171	1	1	0	0	0	0	0	0	0
172	1	1	0	0	0	0	0	0	0
173	1	1	0	0	0	0	0	0	0
174	1	1	0	0	0	0	0	0	0
175	1	1	1	0	0	0	0	0	0
176	1	1	0	1	0	0	0	0	0
177	1	1	0	0	0	0	0	0	0
178	1	1	1	0	0	0	0	0	0
179	1	1	1	0	0	0	0	0	0
180	1	1	0	1	0	0	0	1	0
181	0	0	0	0	1	1	0	0	0
182	0	0	0	0	1	1	0	0	0
183	1	0	0	0	0	0	0	0	0
184	0	0	0	0	0	0	0	0	0
185	1	0	0	0	1	0	0	0	0
186	1	0	1	0	1	1	0	0	0
187	0	0	0	1	0	0	0	0	0
188	1	0	1	0	1	1	0	0	1
189	1	0	0	1	0	0	0	1	0
190	0	0	0	0	1	1	0	0	1
191	1	0	1	0	1	0	0	0	1

192	0	0	1	0	1	0	0	1	1
193	0	0	1	0	0	0	0	0	0
194	0	0	1	0	1	1	0	1	0
195	0	0	0	0	0	0	0	0	0
196	0	0	0	1	1	0	0	1	0
197	1	0	0	1	0	0	0	0	0
198	0	0	0	1	0	0	0	0	0
199	0	0	0	1	0	0	0	0	0
200	0	0	0	0	1	0	1	1	0
201	1	0	0	0	0	0	0	0	0
202	1	0	0	1	1	1	0	1	0
203	1	0	1	0	0	0	0	0	0
204	0	0	0	0	0	0	1	0	0
205	0	0	0	0	0	0	0	0	0
206	1	0	0	0	1	0	0	1	1
207	1	0	0	0	0	0	0	1	1
208	1	0	0	0	0	0	0	0	0
209	0	0	0	0	0	0	0	0	0
210	0	0	0	0	0	0	0	0	0
211	1	0	0	0	0	0	0	0	0
212	0	0	0	0	0	0	0	0	0
213	0	0	0	0	0	0	0	0	0
214	1	0	1	0	1	1	0	0	0
215	1	0	0	0	0	0	0	0	0
216	1	0	1	0	0	0	0	0	0
217	1	0	0	0	0	0	0	0	0
218	0	0	0	0	0	0	0	0	0
219	1	0	0	0	0	0	0	0	0
220	1	0	0	0	0	0	0	0	0
221	1	0	0	0	0	0	0	0	0
222	1	0	1	0	0	0	0	0	0
223	1	0	0	0	0	0	0	0	0

224	0	0	0	0	0	0	0	0	0
225	1	0	0	0	0	0	0	0	0
226	1	0	0	0	0	0	0	0	0
227	1	0	0	0	0	0	0	0	0
228	0	0	0	0	0	0	0	0	0
229	0	0	0	0	0	0	0	0	0
230	1	0	0	0	0	0	0	0	0
231	0	0	0	0	1	0	0	0	0
232	1	0	0	1	0	1	1	0	0
233	1	1	0	0	0	0	1	0	0
234	1	1	0	0	0	0	1	0	0
235	1	1	0	0	0	0	1	1	0
236	1	1	1	1	0	0	0	1	0
237	1	0	0	1	0	0	1	1	0
238	0	0	0	1	0	0	1	1	0
239	0	0	0	0	0	0	0	0	0
240	1	1	0	1	0	0	0	1	0
241	1	0	1	0	1	0	1	0	0
242	1	0	0	0	1	1	1	1	0
243	1	0	1	1	1	0	0	0	0
244	1	0	0	1	1	1	0	0	0
245	1	0	1	1	1	0	1	0	0
246	1	0	1	0	1	0	1	0	0
247	1	0	1	1	1	1	1	0	0
248	0	0	1	1	0	0	0	0	0
249	1	0	0	0	1	1	0	0	0
250	1	0	1	1	1	0	1	0	0
251	0	0	1	0	1	0	0	0	0
252	1	0	0	0	0	0	0	0	0
253	1	0	0	1	1	0	0	0	0
254	1	0	0	1	0	0	0	0	0
255	1	0	0	0	0	0	1	0	0

256	0	0	0	0	1	1	0	0	0
257	1	0	0	0	0	0	0	0	0
258	1	0	1	0	1	0	0	0	0
259	1	0	0	1	0	0	0	0	0
260	0	0	0	0	0	0	0	0	0
261	1	0	0	0	0	0	0	0	0
262	1	0	0	0	0	0	0	0	0
263	1	0	1	0	0	0	0	0	0
264	0	0	1	0	1	1	0	0	0
265	1	0	0	0	0	1	0	0	0
266	0	0	1	0	1	1	0	0	0
267	0	1	0	0	0	1	0	0	0
268	0	0	0	0	0	0	0	0	0
269	1	0	0	0	1	0	0	0	0
270	1	0	1	0	1	0	1	0	1
271	0	0	0	1	1	0	0	0	0
272	0	0	0	0	1	1	1	0	0
273	0	0	0	0	0	0	0	0	0
274	0	0	0	0	0	0	0	0	0
275	1	1	0	0	0	0	0	0	0
276	1	0	1	0	1	1	1	1	1
277	1	0	0	0	0	0	0	0	0
278	1	0	0	0	1	0	1	1	0
279	1	0	0	0	1	0	0	0	0
280	0	0	0	0	1	0	1	0	0
281	0	0	0	0	0	0	0	0	0
282	1	0	1	1	1	1	0	0	0
283	0	0	0	0	0	0	0	0	0
284	1	1	1	1	1	1	1	0	0
285	1	1	1	1	1	1	1	0	0
286	0	1	0	0	1	1	1	0	0
287	1	1	1	1	1	1	1	0	0

288	1	0	0	0	0	0	0	0	0
289	0	0	0	0	0	0	0	0	0
290	0	0	0	0	0	0	0	0	0
291	0	0	0	1	0	0	0	0	1
292	0	0	0	0	0	0	0	0	0
293	0	0	0	0	0	0	0	0	0
294	0	0	0	0	0	0	0	0	0
295	0	0	0	0	0	0	0	0	0
296	0	0	0	0	0	0	0	0	0
297	0	0	0	0	0	0	0	0	0
298	0	0	0	0	0	0	0	0	0
299	0	0	0	0	0	0	0	0	0
300	0	0	0	0	0	0	0	0	0
301	0	0	0	0	0	0	0	0	0
302	0	0	0	0	0	0	0	0	0
303	0	0	0	0	0	0	0	0	0
304	0	0	0	0	0	0	0	0	0
305	0	0	0	0	0	0	0	0	0
306	0	0	0	0	0	0	0	0	0
307	0	0	0	0	0	0	0	0	0
308	1	0	0	0	0	0	0	0	0
309	1	0	0	0	0	0	0	0	0
310	0	0	0	0	0	0	0	0	0
311	0	0	0	0	0	0	0	0	0
312	0	0	0	0	0	0	0	0	0
313	0	0	0	0	0	0	0	0	0
314	0	0	0	0	0	0	0	0	0
315	0	0	0	0	0	0	0	0	0
316	0	0	0	0	0	0	0	0	0
317	0	0	0	0	0	0	0	0	0
318	0	0	0	0	0	0	0	0	0
319	1	0	0	0	0	0	0	0	0

320	0	0	0	0	0	0	0	0	0
321	0	0	0	0	0	0	0	0	0
322	0	0	0	0	0	0	0	0	0
323	1	0	0	1	0	0	0	0	0
324	1	0	0	1	0	0	0	0	0
325	1	0	0	1	0	0	0	0	0
326	1	0	0	0	1	0	1	1	0
327	0	0	0	1	0	0	0	0	0
328	0	0	0	0	0	0	0	0	0
329	0	0	0	1	1	0	0	0	0
330	1	0	0	1	1	0	1	1	0
331	1	0	0	1	0	0	0	1	0
332	0	0	0	1	0	0	1	1	0
333	1	0	0	1	0	0	0	0	0
334	0	0	0	0	0	0	0	0	0
335	0	0	0	1	0	0	0	0	0
336	1	0	0	1	0	0	0	0	0
337	0	0	0	0	0	0	0	0	0
338	0	0	0	0	0	0	0	0	0
339	1	0	0	0	0	0	0	0	0
340	1	0	0	0	0	0	0	0	0
341	0	0	0	0	0	0	0	0	0
342	0	0	0	0	0	0	0	0	0
343	0	0	0	1	0	0	0	0	0
344	0	0	0	0	0	0	0	1	0
345	0	0	0	0	0	0	0	1	0
346	1	0	0	1	0	0	1	1	0
347	0	0	0	0	0	0	0	0	0
348	1	0	0	1	0	0	0	0	0
349	0	0	0	0	0	0	0	0	0
350	0	0	0	0	0	0	0	0	0
351	0	0	0	0	0	0	0	0	0

352	1	0	0	0	0	0	0	0	0
353	1	0	0	0	0	0	0	0	0
354	1	0	0	0	0	0	0	0	0
355	1	0	0	0	0	0	0	0	0
356	1	0	0	0	0	0	0	0	0
357	1	0	0	0	0	0	0	0	0
358	1	0	0	0	0	0	0	0	0
359	0	0	0	0	0	0	0	0	0
360	0	0	0	0	0	0	0	0	0
361	0	0	0	0	0	0	0	0	0
362	0	0	0	0	0	0	0	0	0
363	1	0	0	0	0	0	0	0	0
364	0	0	0	0	0	0	0	0	0
365	0	0	0	0	0	0	0	0	0
366	0	0	0	0	0	0	0	0	0
367	0	0	0	0	0	0	0	0	0
368	1	0	1	0	0	0	0	0	1
369	0	0	0	0	0	0	0	0	0
370	0	0	0	0	0	0	0	0	0
371	0	0	0	0	0	0	0	0	0
372	0	0	0	0	0	0	0	0	0
373	1	0	0	0	0	0	0	0	0
374	0	0	0	0	0	0	0	0	0
375	0	0	0	0	0	0	0	0	0
376	0	0	0	0	0	0	0	0	0
377	0	0	0	0	0	0	0	0	0
378	0	0	0	0	0	0	0	0	0
379	0	0	0	0	0	0	0	0	0
380	1	1	0	0	0	0	0	0	0
381	0	0	0	0	0	0	0	0	0
382	0	0	1	0	0	1	0	0	1
383	1	0	0	0	0	0	0	0	0

384	0	0	0	0	0	0	0	0	0
385	1	0	0	0	0	0	0	0	0
386	1	0	0	0	0	0	1	1	1
387	1	0	0	0	1	0	0	0	0
388	1	0	0	0	0	0	1	0	0
389	1	0	1	1	1	1	0	0	0
390	1	0	0	0	0	0	0	0	0
391	1	0	0	1	0	0	1	0	1
392	1	0	1	1	0	0	1	0	1
393	1	0	1	0	0	0	0	1	1
394	1	0	0	0	0	0	0	1	1
395	1	0	0	0	0	0	0	0	0
396	1	1	1	1	1	1	1	1	1
397	1	1	0	0	0	0	0	1	1
398	1	0	0	0	0	0	0	1	1
399	0	0	0	0	0	1	0	0	1
400	1	0	0	0	0	0	1	0	1

Intoxicating Substances	Number of consumers
Betel Nut	159
Betel Nut with lime (Process include extra use of lime)	47
Zarda Pan (Dried Betel nut)	57
Mitha Pan (Dried Betel nut)	80
Guthka (Processed Betel nut)	66
Sikhar (Processed Betel nut)	41
Smoking Tonacco	53
Alcohol	48
Chewing Tobacco	24

There are many individual consuming more than one intoxicating substances.
