# Supplementary material for: Bacteria in the oral cavity of individuals consuming intoxicating substances
Source: PLoS One. 2023 May 26;18(5):e0285753. doi: 10.1371/journal.pone.0285753 (PMC10218728; doi:10.1371/journal.pone.0285753)
Supplement: S3 Table — (PDF) [file pone.0285753.s003.pdf]

S3-Table: Record on the pattern of consumption (i.e., number of time consumption, daily/weekly/ monthly) and the total duration of consumption of the intoxicating substances along with their oral hygiene.

PARTICIPANT ID	DAILY (D) /WEEKLY (W)/ MONTHLY (M)) CONSUMPSION																											CONSUMING SINCE (YEARS)	LEAVING CONSUMPTION		BRUSH (NO/DAY)
	BETEL NUT			BETEL NUT LIME (PROCESS INCLUDE EXTRA USE OF LIME)			ZARDAPAN (DRIED BETEL NUT)			MITHAPAN (DRIED BETEL NUT)			GUTHKA (PROCESSED BETEL NUT)			SIKHAR (PROCESSED BETEL NUT)			SMOKING TOBACCO			ALCOHOL			CHEWING TABACCO						
	**D**	**W**	**M**	**D**	**W**	**M**	**D**	**W**	**M**	**D**	**W**	**M**	**D**	**W**	**M**	**D**	**W**	**M**	**D**	**W**	**M**	**D**	**W**	**M**	**D**	**W**	**M**		YEARS	MONTHS	
101	1	0	0	1	0	0	0	1	0	0	0	0	0	0	0	0	0	0	1	0	0	0	0	0	0	0	0	20	0	0	1
102	0	0	1	0	3	0	0	0	0	0	0	1	0	0	0	0	0	0	0	0	0	0	0	0	0	0	0	10	0	0	1
103	0	0	0	0	0	0	0	0	0	0	0	0	0	0	0	0	0	0	0	0	0	0	0	1	0	0	0	10	0	0	1
104	2	0	0	0	0	0	0	0	0	1	0	0	0	0	0	0	0	0	0	0	0	0	0	0	0	0	0	6	0	0	2
105	0	1	0	0	0	0	0	0	0	0	0	0	0	0	0	0	0	0	0	0	0	0	0	0	0	0	0	1	0	0	2
106	0	0	0	0	0	0	0	0	0	0	0	0	0	0	0	0	0	0	0	0	0	0	0	0	0	0	0	0	0	0	1
107	0	0	0	0	0	0	0	0	0	0	0	0	0	0	0	0	0	0	0	0	0	0	0	0	0	0	0	0	0	0	1
108	0	0	0	0	0	0	0	0	0	0	0	0	0	0	0	0	0	0	0	0	0	0	0	0	0	0	0	0	0	0	2
109	0	0	0	0	0	0	0	3	0	0	3	0	0	0	0	0	0	0	1	0	0	0	0	0	0	0	0	15	0	0	2
110	0	0	2	0	0	0	0	0	0	0	0	2	0	0	3	0	0	1	0	1	0	0	0	2	0	0	0	15	0	0	1
111	0	0	1	0	0	0	0	0	0	0	0	1	0	0	0	0	0	0	0	0	0	0	0	0	0	0	0	8	0	0	1
112	6	0	0	0	0	0	0	0	0	0	0	0	0	0	0	0	0	0	1	0	0	0	0	0	0	0	0	2	0	8	2
113	1	0	0	0	0	0	0	0	0	0	0	2	0	0	0	0	0	0	0	0	0	0	0	0	0	0	0	16	0	0	1
114	2	0	0	1	0	0	0	0	1	0	0	0	5	0	0	5	0	0	0	0	0	0	0	1	0	0	0	4	0	0	2
115	0	1	0	0	0	0	0	0	0	0	0	1	0	0	0	0	0	0	0	1	0	0	0	0	0	0	0	14	0	0	1
116	2	0	0	1	0	0	0	0	0	0	0	0	0	0	0	0	0	0	0	3	0	0	0	0	0	0	0	3	0	6	2
117	0	0	0	0	0	0	0	0	0	0	0	1	0	0	0	0	0	0	1	0	0	0	0	0	0	0	0	7	0	5	1
118	3	0	0	0	2	0	0	1	0	0	0	1	0	2	0	0	2	0	5	0	0	0	2	0	0	0	0	8	0	0	1
119	0	2	0	0	0	0	0	0	1	0	0	1	0	0	0	0	0	0	0	0	0	0	0	0	0	0	0	4	0	0	1
120	5	0	0	0	0	0	0	0	0	0	0	1	0	0	0	0	0	0	0	0	0	0	0	0	0	0	0	15	0	0	2
121	0	0	0	0	0	0	0	0	0	0	1	0	0	0	0	0	1	0	0	1	0	0	0	3	0	0	0	4	0	0	2
122	0	0	0	0	0	0	0	0	0	0	0	0	0	0	0	0	0	0	0	0	0	0	0	0	0	0	0	0	0	0	1
123	0	0	3	0	0	10	0	2	0	0	0	1	1	0	0	1	0	0	0	1	0	0	3	0	0	0	0	5	0	0	1
124	0	0	0	0	0	0	0	0	0	0	0	0	0	0	0	0	0	0	0	0	0	0	0	1	0	0	0	2	0	1	2

125	0	0	0	0	0	0	0	0	0	0	0	0	0	0	0	0	0	0	0	0	0	0	0	0	0	0	0	0	0	0	1
126	0	0	1	0	0	0	0	0	0	0	0	0	0	0	0	0	0	0	0	0	0	0	0	0	0	0	0	2	0	0	1
127	0	0	1	0	0	0	0	0	0	0	0	1	0	0	0	0	0	0	0	0	0	0	0	0	0	0	0	3	0	0	1
128	0	0	0	0	0	0	0	0	0	0	0	1	0	0	0	0	0	0	0	0	0	0	0	0	0	0	0	8	0	2	1
129	0	0	0	0	0	0	0	0	2	0	0	2	0	0	0	0	0	0	10	0	0	0	0	0	0	0	0	6	0	1	2
130	0	0	0	0	0	0	0	0	0	0	0	0	0	0	0	0	0	0	0	0	0	0	0	0	0	0	0	0	0	0	1
131	0	0	0	0	0	0	0	0	0	0	0	1	0	0	0	0	0	0	0	0	0	0	0	0	0	0	0	4	0	0	1
132	0	0	1	0	0	1	0	0	2	0	0	1	0	2	0	0	2	0	0	5	0	0	0	1	0	0	1	10	0	6	1
133	0	7	0	0	0	0	0	0	0	0	0	0	8	0	0	8	0	0	4	0	0	0	0	1	0	0	0	4	0	0	2
134	0	0	0	0	0	0	0	0	0	0	0	0	0	0	0	0	0	0	0	0	0	0	0	0	0	0	0	0	0	0	2
135	1	0	0	3	0	0	0	0	2	0	0	0	3	0	0	1	0	0	0	0	0	0	0	0	0	0	0	5	0	0	1
136	0	0	0	0	0	0	0	0	0	0	0	0	0	0	0	0	0	0	0	0	0	0	0	2	0	0	0	20	0	0	1
137	0	0	0	0	0	0	0	0	0	0	0	0	0	0	0	0	0	0	0	0	0	0	0	0	0	0	0	0	0	0	1
138	0	0	1	0	0	0	0	0	0	0	2	0	0	0	0	0	0	0	0	0	0	1	0	0	0	0	0	4	0	0	1
139	0	0	2	0	0	0	0	0	3	0	0	1	0	0	0	0	0	5	2	0	0	0	0	3	0	0	0	4	0	0	2
140	0	0	0	0	0	0	0	0	0	0	0	0	0	0	0	0	0	0	0	0	0	0	0	0	0	0	0	0	0	0	2
141	0	0	0	0	0	0	0	0	0	0	0	0	0	0	0	0	0	0	5	0	0	0	0	0	0	0	0	2	0	6	1
142	0	1	0	0	0	0	0	1	0	0	0	1	0	0	2	0	0	0	3	0	0	0	0	0	0	0	0	4	0	1	1
143	8	0	0	8	0	0	0	0	0	0	1	0	2	0	0	0	0	0	0	0	0	0	0	0	0	0	0	2	0	1	1
144	4	0	0	6	0	0	0	0	0	0	1	0	0	0	0	0	0	0	0	0	0	0	0	0	0	0	0	10	0	0	2
145	0	0	0	0	0	0	0	0	0	0	0	0	0	0	0	0	0	0	0	0	0	0	0	0	0	0	0	0	0	0	1
146	3	0	0	1	0	0	1	0	0	1	0	0	0	0	0	1	0	0	0	0	0	0	0	1	0	0	0	28	0	0	2
147	0	1	0	0	1	0	0	0	0	0	0	0	0	0	0	4	0	0	0	0	0	0	0	0	0	1	0	2	0	1	1
148	0	3	0	0	1	0	0	0	0	0	2	0	0	3	0	0	0	0	0	0	0	0	0	0	0	0	0	7	0	3	1
149	0	0	0	0	0	0	0	0	0	0	0	0	0	0	0	0	0	0	0	0	0	0	0	0	0	0	0	0	0	0	2
150	0	3	0	0	0	0	0	0	0	0	0	0	0	2	0	0	0	0	2	0	0	0	0	0	0	0	0	2	0	1	2
151	0	3	0	0	0	0	0	0	0	0	0	1	0	3	0	0	0	0	1	0	0	0	0	1	0	0	0	2	0	0	1
152	0	0	0	0	0	0	0	0	0	0	0	2	0	0	0	0	0	0	5	0	0	0	1	0	0	0	0	4	1	0	2
153	0	0	0	0	0	0	0	0	0	0	0	2	0	0	0	0	0	0	0	0	0	0	0	0	0	0	0	2	0	0	2
154	0	0	1	0	0	1	0	0	0	0	0	1	0	0	0	0	0	0	0	0	0	0	0	0	0	0	0	3	6	0	2
155	0	2	0	0	2	0	0	0	2	0	0	0	3	0	0	0	0	0	0	0	0	0	0	0	0	0	0	4	0	0	1
156	0	0	0	0	0	0	1	0	0	0	0	0	1	0	0	1	0	0	0	0	0	0	0	0	0	0	0	6	1	0	3

157	0	0	0	0	0	0	0	0	0	0	0	1	0	0	0	0	0	0	0	0	0	0	0	0	0	0	0	10	1	0	1
158	0	0	0	0	0	0	0	0	0	0	1	0	0	0	0	0	0	0	0	0	0	0	0	0	0	0	0	3	0	0	2
159	0	0	0	0	0	1	0	0	0	0	0	0	0	3	0	0	0	0	0	0	0	0	0	0	0	0	0	1	0	0	2
160	0	0	0	0	0	0	0	0	0	0	0	0	0	4	0	0	0	0	0	0	0	0	0	0	0	0	0	2	0	0	2
161	5	0	0	5	0	0	0	0	2	0	0	2	0	0	0	0	0	0	0	0	0	1	0	0	0	0	0	35	0	0	2
162	0	0	0	0	0	0	0	0	0	0	0	0	12	0	0	0	0	0	0	0	0	1	0	0	0	0	0	24	0	6	2
163	10	0	0	7	0	0	0	0	2	0	0	0	0	0	0	0	0	0	0	0	0	0	0	5	0	0	0	8	0	0	2
164	2	0	0	2	0	0	0	0	0	0	0	0	0	0	0	0	0	0	0	0	0	0	0	0	0	0	0	10	0	0	2
165	8	0	0	8	0	0	0	0	0	0	0	0	0	0	0	0	0	0	0	0	0	0	0	0	0	0	0	50	0	1	2
166	0	3	0	0	0	0	0	0	0	0	0	0	0	0	0	0	0	0	0	0	0	0	0	0	0	0	0	9	0	0	1
167	1	0	0	0	0	0	0	0	0	0	0	0	0	0	0	0	0	0	0	0	0	1	0	0	0	0	0	17	0	0	2
168	4	0	0	4	0	0	0	0	0	0	0	0	0	0	0	0	0	0	0	0	0	0	0	0	0	0	0	30	0	0	2
169	6	0	0	6	0	0	0	0	0	0	0	0	0	0	0	0	0	0	0	0	0	0	0	0	0	0	0	20	0	0	2
170	6	0	0	6	0	0	1	0	0	0	0	0	0	0	0	0	0	0	0	0	0	0	0	0	0	0	0	34	5	0	2
171	5	0	0	5	0	0	0	0	0	0	0	0	0	0	0	0	0	0	0	0	0	0	0	0	0	0	0	29	0	6	2
172	4	0	0	4	0	0	0	0	0	0	0	0	0	0	0	0	0	0	0	0	0	0	0	0	0	0	0	3	0	0	2
173	4	0	0	4	0	0	0	0	0	0	0	0	0	0	0	0	0	0	0	0	0	0	0	0	0	0	0	10	0	0	1
174	10	0	0	10	0	0	0	0	0	0	0	0	0	0	0	0	0	0	0	0	0	0	0	0	0	0	0	12	0	0	2
175	3	0	0	3	0	0	0	0	1	0	0	0	0	0	0	0	0	0	0	0	0	0	0	0	0	0	0	10	0	0	2
176	2	0	0	2	0	0	0	0	0	0	0	1	0	0	0	0	0	0	0	0	0	0	0	0	0	0	0	2	0	0	2
177	8	0	0	8	0	0	0	0	0	0	0	0	0	0	0	0	0	0	0	0	0	0	0	0	0	0	0	30	0	0	2
178	6	0	0	6	0	0	0	0	1	0	0	0	0	0	0	0	0	0	0	0	0	0	0	0	0	0	0	22	0	0	3
179	10	0	0	10	0	0	10	0	0	0	0	0	0	0	0	0	0	0	0	0	0	0	0	0	0	0	0	22	0	0	2
180	7	0	0	7	0	0	0	0	0	0	0	0	0	0	0	0	0	0	0	0	0	1	0	0	0	0	0	40	0	0	1
181	0	0	0	0	0	0	0	0	0	0	0	0	1	0	0	1	0	0	0	0	0	0	0	0	0	0	0	2	0	0	1
182	0	0	0	0	0	0	0	0	0	0	0	0	3	0	0	3	0	0	0	0	0	0	0	0	0	0	0	6	0	1	1
183	3	0	0	0	0	0	0	0	0	0	0	0	0	0	0	0	0	0	0	0	0	0	0	0	0	0	0	2	0	3	1
184	0	0	0	0	0	0	0	0	0	0	0	0	0	0	0	0	0	0	0	0	0	0	0	0	0	0	0	0	0	0	1
185	3	0	0	0	0	0	0	0	0	0	0	0	8	0	0	0	0	0	0	0	0	0	0	0	0	0	0	2	0	2	1
186	6	0	0	0	0	0	0	2	0	0	0	0	2	0	0	2	0	0	0	0	0	0	0	0	0	0	0	5	0	0	1

187	0	0	0	0	0	0	0	0	0	0	0	3	0	0	0	0	0	0	0	0	0	0	0	0	0	0	0	12	0	0	1
188	3	0	0	0	0	0	0	0	3	0	0	0	1	0	0	0	3	0	0	0	0	0	0	0	4	0	0	17	0	5	1
189	0	0	1	0	0	0	0	0	0	0	0	0	0	0	0	0	0	0	0	0	0	0	0	1	0	0	0	10	0	0	2
190	0	0	0	0	0	0	0	0	0	0	0	0	5	0	0	5	0	0	0	0	0	0	0	0	6	0	0	5	0	0	2
191	10	0	0	0	0	0	0	1	0	0	0	0	3	0	0	0	0	0	0	0	0	0	0	0	10	0	0	30	0	0	1
192	0	0	0	0	0	0	3	0	0	0	0	0	7	0	0	0	0	0	0	0	0	0	5	0	10	5	0	30	0	0	1
193	0	0	0	0	0	0	2	0	0	0	0	0	0	0	0	0	0	0	0	0	0	0	0	0	0	0	0	1	0	0	2
194	0	0	0	0	0	0	1	0	0	0	0	0	6	0	0	6	0	0	0	0	0	0	0	2	0	0	0	4	0	0	2
195	0	0	0	0	0	0	0	0	0	0	0	0	0	0	0	0	0	0	0	0	0	0	0	0	0	0	0	0	0	0	1
196	0	0	0	0	0	0	0	0	0	0	4	0	32	0	0	0	0	0	0	0	0	0	3	0	0	0	0	8	0	3	1
197	3	0	0	0	0	0	0	0	0	0	0	2	0	0	0	0	0	0	0	0	0	0	0	0	0	0	0	15	1	0	1
198	0	0	0	0	0	0	0	0	0	0	0	1	0	0	0	0	0	0	0	0	0	0	0	0	0	0	0	0	0	0	1
199	0	0	0	0	0	0	0	0	0	0	0	1	0	0	0	0	0	0	0	0	0	0	0	0	0	0	0	0	0	0	1
200	0	0	0	0	0	0	0	0	0	0	0	0	1	0	0	0	0	0	5	0	0	0	0	1	0	0	0	20	0	0	1
201	2	0	0	0	0	0	0	0	0	0	0	0	0	0	0	0	0	0	0	0	0	0	0	0	0	0	0	50	0	0	1
202	2	0	0	0	0	0	0	0	0	0	0	1	1	0	0	1	0	0	0	0	0	0	0	1	0	0	0	5	0	0	2
203	10	0	0	0	0	0	10	0	0	0	0	0	0	0	0	0	0	0	0	0	0	0	0	0	0	0	0	18	0	0	2
204	0	0	0	0	0	0	0	0	0	0	0	0	0	0	0	0	0	0	0	35	0	0	0	0	0	0	0	18	0	2	1
205	0	0	0	0	0	0	0	0	0	0	0	0	0	0	0	0	0	0	0	0	0	0	0	0	0	0	0	0	0	0	1
206	10	0	0	0	0	0	0	0	0	0	0	0	10	0	0	0	0	0	0	0	0	1	0	0	0	14	0	10	0	0	1
207	3	0	0	0	0	0	0	0	0	0	0	0	0	0	0	0	0	0	0	0	0	0	0	3	3	0	0	10	0	0	3
208	3	0	0	0	0	0	0	0	0	0	0	0	0	0	0	0	0	0	0	0	0	0	0	0	0	0	0	15	0	0	2
209	0	0	0	0	0	0	0	0	0	0	0	0	0	0	0	0	0	0	0	0	0	0	0	0	0	0	0	0	0	0	1
210	0	0	0	0	0	0	0	0	0	0	0	0	0	0	0	0	0	0	0	0	0	0	0	0	0	0	0	0	0	0	2
211	10	0	0	0	0	0	0	0	0	0	0	0	0	0	0	0	0	0	0	0	0	0	0	0	0	0	0	10	0	0	2
212	0	0	0	0	0	0	0	0	0	0	0	0	0	0	0	0	0	0	0	0	0	0	0	0	0	0	0	0	0	0	2
213	0	0	0	0	0	0	0	0	0	0	0	0	0	0	0	0	0	0	0	0	0	0	0	0	0	0	0	0	0	0	2
214	2	0	0	0	0	0	0	1	0	0	0	0	2	0	0	2	0	0	0	0	0	0	0	0	0	0	0	1	0	0	1

215	3	0	0	0	0	0	0	0	0	0	0	0	0	0	0	0	0	0	0	0	0	0	0	0	0	0	0	10	0	0	1
216	10	0	0	0	0	0	10	0	0	0	0	0	0	0	0	0	0	0	0	0	0	0	0	0	0	0	0	40	0	0	1
217	2	0	0	0	0	0	0	0	0	0	0	0	0	0	0	0	0	0	0	0	0	0	0	0	0	0	0	8	0	0	1
218	0	0	0	0	0	0	0	0	0	0	0	0	0	0	0	0	0	0	0	0	0	0	0	0	0	0	0	0	0	0	1
219	1	0	0	0	0	0	0	0	0	0	0	0	0	0	0	0	0	0	0	0	0	0	0	0	0	0	0	5	0	0	2
220	1	0	0	0	0	0	0	0	0	0	0	0	0	0	0	0	0	0	0	0	0	0	0	0	0	0	0	2	0	0	1
221	2	0	0	0	0	0	0	0	0	0	0	0	0	0	0	0	0	0	0	0	0	0	0	0	0	0	0	4	0	0	1
222	10	0	0	0	0	0	10	0	0	0	0	0	0	0	0	0	0	0	0	0	0	0	0	0	0	0	0	10	0	0	3
223	1	0	0	0	0	0	0	0	0	0	0	0	0	0	0	0	0	0	0	0	0	0	0	0	0	0	0	1	0	0	1
224	0	0	0	0	0	0	0	0	0	0	0	0	0	0	0	0	0	0	0	0	0	0	0	0	0	0	0	0	0	0	1
225	2	0	0	0	0	0	0	0	0	0	0	0	0	0	0	0	0	0	0	0	0	0	0	0	0	0	0	4	0	0	2
226	10	0	0	0	0	0	0	0	0	0	0	0	0	0	0	0	0	0	0	0	0	0	0	0	0	0	0	15	0	0	3
227	6	0	0	0	0	0	0	0	0	0	0	0	0	0	0	0	0	0	0	0	0	0	0	0	0	0	0	15	0	0	3
228	0	0	0	0	0	0	0	0	0	0	0	0	0	0	0	0	0	0	0	0	0	0	0	0	0	0	0	0	0	0	1
229	0	0	0	0	0	0	0	0	0	0	0	0	0	0	0	0	0	0	0	0	0	0	0	0	0	0	0	0	0	0	1
230	1	0	0	0	0	0	0	0	0	0	0	0	0	0	0	0	0	0	0	0	0	0	0	0	0	0	0	1	0	0	1
231	0	0	0	0	0	0	0	0	0	0	0	0	3	0	0	0	0	0	0	0	0	0	0	0	0	0	0	14	0	0	2
232	3	0	0	0	0	0	0	0	0	0	1	0	0	0	0	6	0	0	3	0	0	0	0	0	0	0	0	8	0	0	1
233	1	0	0	1	0	0	0	0	0	0	0	0	0	0	0	0	0	0	0	1	0	0	0	0	0	0	0	13	0	0	1
234	1	0	0	1	0	0	0	0	0	0	0	0	0	0	0	0	0	0	0	1	0	0	0	0	0	0	0	30	0	0	1
235	4	0	0	4	0	0	0	0	0	0	0	0	0	0	0	0	0	0	0	14	0	0	2	0	0	0	0	35	0	0	1
236	15	0	0	15	0	0	4	0	0	4	0	0	0	0	0	0	0	0	0	0	0	2	0	0	0	0	0	30	0	8	1
237	2	0	0	0	0	0	0	0	0	0	1	0	0	0	0	0	0	0	4	0	0	0	2	0	0	0	0	5	0	0	1
238	0	0	0	0	0	0	0	0	0	0	1	0	0	0	0	0	0	0	1	0	0	0	1	0	0	0	0	10	4	0	1
239	0	0	0	0	0	0	0	0	0	0	0	0	0	0	0	0	0	0	0	0	0	0	0	0	0	0	0	0	0	0	1
240	2	0	0	2	0	0	0	0	0	1	0	0	0	0	0	0	0	0	0	0	0	0	0	0	0	0	0	3	0	0	2
241	0	1	0	0	0	0	0	1	0	0	0	0	0	1	0	0	0	0	3	0	0	0	0	0	0	0	0	8	0	0	1
242	2	0	0	0	0	0	0	0	0	0	0	0	0	21	0	0	21	0	3	0	0	0	7	0	0	0		25	1	0	2
243	3	0	0	0	0	0	1	0	0	1	0	0	1	0	0	0	0	0	0	0	0	0	0	0	0	0	0	10	0	0	1

244	0	2	0	0	0	0	0	0	0	0	0	1	18	0	0	18	0	0	0	0	0	0	0	0	0	0	0	10	0	2	1
245	1	0	0	0	0	0	0	0	1	0	0	1	9	0	0	0	0	0	5	0	0	0	0	0	0	0	0	6	0	0	1
246	9	0	0	0	0	0	0	0	2	0	0	0	4	0	0	0	0	0	0	12	0	0	0	0	0	0	0	4	0	0	2
247	2	0	0	0	0	0	0	1	0	0	1	0	30	0	0	5	0	0	10	0	0	0	0	0	0	0	0	10	0	0	1
248	0	0	0	0	0	0	0	2	0	0	2	0	0	0	0	0	0	0	0	0	0	0		0	0		0	4	0	0	1
249	1	0	0	0	0	0	0	0	0	0	0	0	36	0	0	3	0	0	0	0	0	0	0	0	0	0	0	12	0	3	2
250	4	0	0	0	0	0	2	0	0	0	0	0	0	0	0	0	0	0	9	0	0	0	0	0	0	0	0	6	0	0	2
251	0	0	0	0	0	0	4	0	0	0	0	0	2	0	0	0	0	0	0	0	0	0	0	0	0	0	0	4	0	0	1
252	1	0	0	0	0	0	0	0	0	0	0	0	0	0	0	0	0	0	0	0	0	0	0	0	0	0	0	20	0	0	2
253	1	0	0	0	0	0	0	0	0	1	0	0	2	0	0	0	0	0	0	0	0	0	0	0	0	0	0	16	0	0	2
254	0	5	0	0	0	0	0	0	0	0	0	1	0	0	0	0	0	0	0	0	0	0	0	0	0	0	0	7	0	0	1
255	30	0	0	0	0	0	0	0	0	0	0	0	0	0	0	0	0	0	20	0	0	0	0	0	0	0	0	35	0	0	1
256	0	0	0	0	0	0	0	0	0	0	0	0	0	14	0	0	14	0	0	0	0	0	0	0	0	0	0	10	0	0	1
257	5	0	0	0	0	0	0	0	0	0	0	0	0	0	0	0	0	0	0	0	0	0	0	0	0	0	0	35	0	0	2
258	5	0	0	0	0	0	2	0	0	0	0	0	2	0	0	0	0	0	0	0	0	0	0	0	0	0	0	20	0	3	2
259	2	0	0	0	0	0	0	0	0	0	0	1	0	0	0	0	0	0	0	0	0	0	0	0	0	0	0	10	0	0	1
260	0	0	0	0	0	0	0	0	0	0	0	0	0	0	0	0	0	0	0	0	0	0	0	0	0	0	0	0	0	0	1
261	0	5	0	0	0	0	0	0	0	0	0	0	0	0	0	0	0	0	0	0	0	0	0	0	0	0	0	35	0	0	1
262	2	0	0	0	0	0	0	0	0	0	0	0	0	0	0	0	0	0	0	0	0	0	0	0	0	0	0	16	0	0	1
263	1	0	0	0	0	0	3	0	0	0	0	0	0	0	0	0	0	0	0	0	0	0	0	0	0	0	0	10	0	1	2
264	0	0	0	0	0	0	1	0	0	0	0	0	8	0	0	4	0	0	0	0	0	0	0	0	0	0	0	15	0	0	1
265	0	0	0	0	0	0	0	0	0	0	0	0	0	0	0	6	0	0	0	0	0	0	0	0	0	0	0	4	0	0	1
266	0	0	0	0	0	0	3	0	0	0	0	0	3	0	0	3	0	0	0	0	0	0	0	0	0	0	0	5	0	0	2
267	0	0	0	0	0	0	0	0	0	0	0	0	0	0	0	24	0	0	0	0	0	0	0	0	0	0	0	8	0	1	0
268	0	0	0	0	0	0	0	0	0	0	0	0	0	0	0	0	0	0	0	0	0	0	0	0	0	0	0	0	0	0	2
269	2	0	0	0	0	0	0	0	0	0	0	0	4	0	0	0	0	0	0	0	0	0	0	0	0	0	0	3	0	2	1
270	3	0	0	0	0	0	0	2	0	0	0	0	0	2	0	0	0	0	0	0	0	0	0	0	0	0	0	15	0	0	1
271	0	0	0	0	0	0	0	0	0	0	0	1	6	0	0	0	0	0	0	0	0	0	0	0	0	0	0	7	0	0	1

272	0	0	0	0	0	0	0	0	0	0	0	0	2	0	0	2	0	0	2	0	0	0	0	0	0	0	0	5	0	0	2
273	0	0	0	0	0	0	0	0	0	0	0	0	0	0	0	0	0	0	0	0	0	0	0	0	0	0	0	0	0	0	1
274	0	0	0	0	0	0	0	0	0	0	0	0	0	0	0	0	0	0	0	0	0	0	0	0	0	0	0	0	0	0	1
275	3	0	0	1	0	0	0	0	0	0	0	0	0	0	0	0	0	0	0	0	0	0	0	0	0	0	0	6	0	3	1
276	10	0	0	0	0	0	1	0	0	0	0	0	2	0	0	2	0	0	0	0	1	0	0	1	0	0	1	7	0	0	2
277	5	0	0	0	0	0	0	0	0	0	0	0	0	0	0	0	0	0	0	0	0	0	0	0	0	0	0	1	0	0	2
278	2	0	0	0	0	0	0	0	0	0	0	0	4	0	0	0	0	0	1	0	0	0	0	4	0	0	0	5	0	0	1
279	4	0	0	0	0	0	0	0	0	0	0	0	2	0	0	0	0	0	0	0	0	0	0	0	0	0	0	1	0	0	2
280	0	0	0	0	0	0	0	0	0	0	0	0	4	0	0	0	0	0	4	0	0	0	0	0	0	0	0	6	0	0	2
281	0	0	0	0	0	0	0	0	0	0	0	0	0	0	0	0	0	0	0	0	0	0	0	0	0	0	0	0	0	0	2
282	3	0	0	0	2	0	4	0	0	0	1	0	7	0	0	3	0	0	0	0	0	0	0	0	0	0	0	8	0	0	3
283	0	0	0	0	0	0	0	0	0	0	0	0	0	0	0	0	0	0	0	0	0	0	0	0	0	0	0	0	0	0	1
284	2	0	0	2	0	0	0	1	0	0	1	0	2	0	0	2	0	0	1	0	0	0	0	0	0	0	0	4	0	2	2
285	2	0	0	0	2	0	2	0	0	4	0	0	0	2	0	0	2	0	5	0	0	0	0	0	0	0	0	5	0	6	2
286	0	0	0	5	0	0	0	0	0	0	0	0	5	0	0	5	0	0	5	0	0	0	0	0	0	0	0	3	0	0	1
287	2	0	0	1	0	0	0	2	0	0	0	1	1	0	0	1	0	0	1	0	0	0	0	0	0	0	0	5	0	0	1
288	6	0	0	0	0	0	0	0	0	0	0	0	0	0	0	0	0	0	0	0	0	0	0	0	0	0	0	5	0	0	1
289	0	0	0	0	0	0	0	0	0	0	0	0	0	0	0	0	0	0	0	0	0	0	0	0	0	0	0	0	0	0	1
290	0	0	0	0	0	0	0	0	0	0	0	0	0	0	0	0	0	0	0	0	0	0	0	0	0	0	0	0	0	0	1
291	0	0	0	0	0	0	0	0	0	0	1	0	0	0	0	0	0	0	0	0	0	0	0	0	3	0	0	1	0	0	1
292	0	0	0	0	0	0	0	0	0	0	0	0	0	0	0	0	0	0	0	0	0	0	0	0	0	0	0	0	0	0	1
293	0	0	0	0	0	0	0	0	0	0	0	0	0	0	0	0	0	0	0	0	0	0	0	0	0	0	0	0	0	0	2
294	0	0	0	0	0	0	0	0	0	0	0	0	0	0	0	0	0	0	0	0	0	0	0	0	0	0	0	0	0	0	0
295	0	0	0	0	0	0	0	0	0	0	0	0	0	0	0	0	0	0	0	0	0	0	0	0	0	0	0	0	0	0	2
296	0	0	0	0	0	0	0	0	0	0	0	0	0	0	0	0	0	0	0	0	0	0	0	0	0	0	0	0	0	0	1
297	0	0	0	0	0	0	0	0	0	0	0	0	0	0	0	0	0	0	0	0	0	0	0	0	0	0	0	0	0	0	1
298	0	0	0	0	0	0	0	0	0	0	0	0	0	0	0	0	0	0	0	0	0	0	0	0	0	0	0	0	0	0	1
299	0	0	0	0	0	0	0	0	0	0	0	0	0	0	0	0	0	0	0	0	0	0	0	0	0	0	0	0	0	0	1
300	0	0	0	0	0	0	0	0	0	0	0	0	0	0	0	0	0	0	0	0	0	0	0	0	0	0	0	0	0	0	1
301	0	0	0	0	0	0	0	0	0	0	0	0	0	0	0	0	0	0	0	0	0	0	0	0	0	0	0	0	0	0	1
302	0	0	0	0	0	0	0	0	0	0	0	0	0	0	0	0	0	0	0	0	0	0	0	0	0	0	0	0	0	0	1
303	0	0	0	0	0	0	0	0	0	0	0	0	0	0	0	0	0	0	0	0	0	0	0	0	0	0	0	0	0	0	1

304	0	0	0	0	0	0	0	0	0	0	0	0	0	0	0	0	0	0	0	0	0	0	0	0	0	0	0	0	0	0	1
305	0	0	0	0	0	0	0	0	0	0	0	0	0	0	0	0	0	0	0	0	0	0	0	0	0	0	0	0	0	0	1
306	0	0	0	0	0	0	0	0	0	0	0	0	0	0	0	0	0	0	0	0	0	0	0	0	0	0	0	0	0	0	2
307	0	0	0	0	0	0	0	0	0	0	0	0	0	0	0	0	0	0	0	0	0	0	0	0	0	0	0	0	0	0	2
308	0	0	1	0	0	0	0	0	0	0	0	0	0	0	0	0	0	0	0	0	0	0	0	0	0	0	0	7	0	0	2
309	0	0	2	0	0	0	0	0	0	0	0	0	0	0	0	0	0	0	0	0	0	0	0	0	0	0	0	10	0	0	2
310	0	0	0	0	0	0	0	0	0	0	0	0	0	0	0	0	0	0	0	0	0	0	0	0	0	0	0	0	0	0	2
311	0	0	0	0	0	0	0	0	0	0	0	0	0	0	0	0	0	0	0	0	0	0	0	0	0	0	0	0	0	0	2
312	0	0	0	0	0	0	0	0	0	0	0	0	0	0	0	0	0	0	0	0	0	0	0	0	0	0	0	0	0	0	1
313	0	0	0	0	0	0	0	0	0	0	0	0	0	0	0	0	0	0	0	0	0	0	0	0	0	0	0	0	0	0	1
314	0	0	0	0	0	0	0	0	0	0	0	0	0	0	0	0	0	0	0	0	0	0	0	0	0	0	0	0	0	0	1
315	0	0	0	0	0	0	0	0	0	0	0	0	0	0	0	0	0	0	0	0	0	0	0	0	0	0	0	0	0	0	1
316	0	0	0	0	0	0	0	0	0	0	0	0	0	0	0	0	0	0	0	0	0	0	0	0	0	0	0	0	0	0	1
317	0	0	0	0	0	0	0	0	0	0	0	0	0	0	0	0	0	0	0	0	0	0	0	0	0	0	0	0	0	0	1
318	0	0	0	0	0	0	0	0	0	0	0	0	0	0	0	0	0	0	0	0	0	0	0	0	0	0	0	0	0	0	1
319	0	1	0	0	0	0	0	0	0	0	0	0	0	0	0	0	0	0	0	0	0	0	0	0	0	0	0	12	0	0	2
320	0	0	0	0	0	0	0	0	0	0	0	0	0	0	0	0	0	0	0	0	0	0	0	0	0	0	0	0	0	0	1
321	0	0	0	0	0	0	0	0	0	0	0	0	0	0	0	0	0	0	0	0	0	0	0	0	0	0	0	0	0	0	2
322	0	0	0	0	0	0	0	0	0	0	0	0	0	0	0	0	0	0	0	0	0	0	0	0	0	0	0	0	0	0	1
323	0	0	2	0	0	0	0	0	0	0	0	1	0	0	0	0	0	0	0	0	0	0	0	0	0	0	0	11	0	0	1
324	0	0	1	0	0	0	0	0	0	0	0	1	0	0	0	0	0	0	0	0	0	0	0	0	0	0	0	8	0	0	1
325	0	0	1	0	0	0	0	0	0	0	0	2	0	0	0	0	0	0	0	0	0	0	0	0	0	0	0	5	1	0	1
326	0	0	6	0	0	0	0	0	0	0	0	0	0	0	10	0	0	0	0	0	12	0	0	3	0	0	0	6	1	0	2
327	0	0	0	0	0	0	0	0	0	0	0	1	0	0	0	0	0	0	0	0	0	0	0	0	0	0	0	0	0	0	1
328	0	0	0	0	0	0	0	0	0	0	0	0	0	0	0	0	0	0	0	0	0	0	0	0	0	0	0	0	0	0	1
329	0	0	0	0	0	0	0	0	0	0	0	1	0	0	4	0	0	0	0	0	0	0	0	0	0	0	0	4	2	0	1
330	6	0	0	0	0	0	0	0	0	1	0	0	1	0	0	0	0	0	3	0	0	1	0	0	0	0	0	6	0	0	1
331	0	0	1	0	0	0	0	0	0	0	0	1	0	0	0	0	0	0	0	0	0	0	0	1	0	0	0	1	0	0	2
332	0	0	0	0	0	0	0	0	0	0	0	2	0	0	0	0	0	0	0	0	2	0	0	3	0	0	0	1	0	0	2
333	0	0	1	0	0	0	0	0	0	0	0	1	0	0	0	0	0	0	0	0	0	0	0	0	0	0	0	5	2	0	2
334	0	0	0	0	0	0	0	0	0	0	0	0	0	0	0	0	0	0	0	0	0	0	0	0	0	0	0	0	0	0	3
335	0	0	0	0	0	0	0	0	0	0	0	1	0	0	0	0	0	0	0	0	0	0	0	0	0	0	0	3	0	0	2

336	0	0	1	0	0	0	0	0	0	0	0	1	0	0	0	0	0	0	0	0	0	0	0	0	0	0	0	6	0	0	2
337	0	0	0	0	0	0	0	0	0	0	0	0	0	0	0	0	0	0	0	0	0	0	0	0	0	0	0	0	0	0	1
338	0	0	0	0	0	0	0	0	0	0	0	0	0	0	0	0	0	0	0	0	0	0	0	0	0	0	0	0	0	0	1
339	0	0	1	0	0	0	0	0	0	0	0	0	0	0	0	0	0	0	0	0	0	0	0	0	0	0	0	0	0	0	1
340	0	0	1	0	0	0	0	0	0	0	0	0	0	0	0	0	0	0	0	0	0	0	0	0	0	0	0	5	0	0	1
341	0	0	0	0	0	0	0	0	0	0	0	0	0	0	0	0	0	0	0	0	0	0	0	0	0	0	0	0	0	0	1
342	0	0	0	0	0	0	0	0	0	0	0	0	0	0	0	0	0	0	0	0	0	0	0	0	0	0	0	0	0	0	2
343	0	0	0	0	0	0	0	0	0	0	0	1	0	0	0	0	0	0	0	0	0	0	0	0	0	0	0	10	0	0	2
344	0	0	0	0	0	0	0	0	0	0	0	0	0	0	0	0	0	0	0	0	0	0	0	3	0	0	0	4	0	2	2
345	0	0	0	0	0	0	0	0	0	0	0	0	0	0	0	0	0	0	0	0	0	0	0	1	0	0	0	8	0	6	1
346	0	2	0	0	0	0	0	0	0	0	0	2	0	0	0	0	0	0	0	0	10	1	0	0	0	0	0	7	0	0	1
347	0	0	0	0	0	0	0	0	0	0	0	0	0	0	0	0	0	0	0	0	0	0	0	0	0	0	0	0	0	0	2
348	0	0	2	0	0	0	0	0	0	0	0	2	0	0	0	0	0	0	0	0	0	0	0	0	0	0	0	5	0	0	2
349	0	0	0	0	0	0	0	0	0	0	0	0	0	0	0	0	0	0	0	0	0	0	0	0	0	0	0	0	0	0	1
350	0	0	0	0	0	0	0	0	0	0	0	0	0	0	0	0	0	0	0	0	0	0	0	0	0	0	0	0	0	0	2
351	0	0	0	0	0	0	0	0	0	0	0	0	0	0	0	0	0	0	0	0	0	0	0	0	0	0	0	0	0	0	1
352	0	0	2	0	0	0	0	0	0	0	0	0	0	0	0	0	0	0	0	0	0	0	0	0	0	0	0	3	0	0	1
353	0	0	4	0	0	0	0	0	0	0	0	0	0	0	0	0	0	0	0	0	0	0	0	0	0	0	0	5	2	0	2
354	0	1	0	0	0	0	0	0	0	0	0	0	0	0	0	0	0	0	0	0	0	0	0	0	0	0	0	8	2	0	1
355	0	2	0	0	0	0	0	0	0	0	0	0	0	0	0	0	0	0	0	0	0	0	0	0	0	0	0	3	0	0	1
356	0	0	2	0	0	0	0	0	0	0	0	0	0	0	0	0	0	0	0	0	0	0	0	0	0	0	0	18	5	0	2
357	0	3	0	0	0	0	0	0	0	0	0	0	0	0	0	0	0	0	0	0	0	0	0	0	0	0	0	10	2	0	1
358	0	0	3	0	0	0	0	0	0	0	0	0	0	0	0	0	0	0	0	0	0	0	0	0	0	0	0	8	0	0	1
359	0	0	0	0	0	0	0	0	0	0	0	0	0	0	0	0	0	0	0	0	0	0	0	0	0	0	0	0	0	0	1
360	0	0	0	0	0	0	0	0	0	0	0	0	0	0	0	0	0	0	0	0	0	0	0	0	0	0	0	0	0	0	1
361	0	0	0	0	0	0	0	0	0	0	0	0	0	0	0	0	0	0	0	0	0	0	0	0	0	0	0	0	0	0	1
362	0	0	0	0	0	0	0	0	0	0	0	0	0	0	0	0	0	0	0	0	0	0	0	0	0	0	0	0	0	0	1
363	0	7	0	0	0	0	0	0	0	0	0	0	0	0	0	0	0	0	0	0	0	0	0	0	0	0	0	25	2	0	2
364	0	0	0	0	0	0	0	0	0	0	0	0	0	0	0	0	0	0	0	0	0	0	0	0	0	0	0	0	0	0	1
365	0	0	0	0	0	0	0	0	0	0	0	0	0	0	0	0	0	0	0	0	0	0	0	0	0	0	0	0	0	0	1
366	0	0	0	0	0	0	0	0	0	0	0	0	0	0	0	0	0	0	0	0	0	0	0	0	0	0	0	0	0	0	2
367	0	0	0	0	0	0	0	0	0	0	0	0	0	0	0	0	0	0	0	0	0	0	0	0	0	0	0	0	0	0	1

368	0	21	0	0	0	0	4	0	0	0	0	0	0	0	0	0	0	0	0	0	0	0	0	0	5	0	0	40	0	0	1
369	0	0	0	0	0	0	0	0	0	0	0	0	0	0	0	0	0	0	0	0	0	0	0	0	0	0	0	0	0	0	2
370	0	0	0	0	0	0	0	0	0	0	0	0	0	0	0	0	0	0	0	0	0	0	0	0	0	0	0	0	0	0	2
371	0	0	0	0	0	0	0	0	0	0	0	0	0	0	0	0	0	0	0	0	0	0	0	0	0	0	0	0	0	0	1
372	0	0	0	0	0	0	0	0	0	0	0	0	0	0	0	0	0	0	0	0	0	0	0	0	0	0	0	0	0	0	1
373	2	0	0	0	0	0	0	0	0	0	0	0	0	0	0	0	0	0	0	0	0	0	0	0	0	0	0	1	0	0	2
374	0	0	0	0	0	0	0	0	0	0	0	0	0	0	0	0	0	0	0	0	0	0	0	0	0	0	0	0	0	0	1
375	0	0	0	0	0	0	0	0	0	0	0	0	0	0	0	0	0	0	0	0	0	0	0	0	0	0	0	0	0	0	1
376	0	0	0	0	0	0	0	0	0	0	0	0	0	0	0	0	0	0	0	0	0	0	0	0	0	0	0	0	0	0	1
377	0	0	0	0	0	0	0	0	0	0	0	0	0	0	0	0	0	0	0	0	0	0	0	0	0	0	0	0	0	0	1
378	0	0	0	0	0	0	0	0	0	0	0	0	0	0	0	0	0	0	0	0	0	0	0	0	0	0	0	0	0	0	1
379	0	0	0	0	0	0	0	0	0	0	0	0	0	0	0	0	0	0	0	0	0	0	0	0	0	0	0	0	0	0	2
380	1	0	0	2	0	0	0	0	0	0	0	0	0	0	0	0	0	0	0	0	0	0	0	0	0	0	0	8	0	1	2
381	0	0	0	0	0	0	0	0	0	0	0	0	0	0	0	0	0	0	0	0	0	0	0	0	0	0	0	0	0	0	2
382	0	0	0	0	0	0	0	0	1	0	0	0	0	0	0	4	0	0	0	0	0	0	0	0	3	0	0	6	0	0	1
383	4	0	0	0	0	0	0	0	0	0	0	0	0	0	0	0	0	0	0	0	0	0	0	0	0	0	0	10	0	0	3
384	0	0	0	0	0	0	0	0	0	0	0	0	0	0	0	0	0	0	0	0	0	0	0	0	0	0	0	0	0	0	2
385	3	0	0	0	0	0	0	0	0	0	0	0	0	0	0	0	0	0	0	0	0	0	0	0	0	0	0	1	0	0	2
386	3	0	0	0	0	0	0	0	0	0	0	0	0	0	0	0	0	0	2	0	0	0	2	0	2	0	0	40	1	0	1
387	4	0	0	0	0	0	0	0	0	0	0	0	1	0	0	0	0	0	0	0	0	0	0	0	0	0	0	20	0	0	1
388	3	0	0	0	0	0	0	0	0	0	0	0	0	0	0	0	0	0	15	0	0	0	0	0	0	0	0	38	0	1	1
389	4	0	0	0	0	0	2	0	0	0	3	0	1	0	0	1	0	0	0	0	0	0	0	0	0	0	0	10	0	0	1
390	12	0	0	0	0	0	0	0	0	0	0	0	0	0	0	0	0	0	0	0	0	0	0	0	0	0	0	30	0	0	2
391	15	0	0	0	0	0	0	0	0	0	0	1	0	0	0	0	0	0	3	0	0	0	0	0	5	0	0	18	0	0	2
392	3	0	0	0	0	0	0	0	2	0	0	1	0	0	0	0	0	0	6	0	0	0	0	0	4	0	0	25	0	0	2
393	2	0	0	0	0	0	0	1	0	0	0	0	0	0	0	0	0	0	0	0	0	0	0	2	6	0	0	24	3	0	1
394	40	0	0	0	0	0	0	0	0	0	0	0	0	0	0	0	0	0	0	0	0	0	1	0	25	0	0	27	0	0	1
395	3	0	0	0	0	0	0	0	0	0	0	0	0	0	0	0	0	0	0	0	0	0	0	0	0	0	0	25	5	0	2
396	4	0	0	3	0	0	0	1	0	0	1	0	0	3	0	0	3	0	12	0	0	1	0	0	1	0	0	23	6	6	1

397	5	0	0	2	0	0	0	0	0	0	0	0	0	0	0	0	0	0	0	0	0	0	2	0	0	2	0	20	0	0	1
398	5	0	0	0	0	0	0	0	0	0	0	0	0	0	0	0	0	0	0	0	0	1	0	0	12	0	0	25	0	0	1
399	0	0	0	0	0	0	0	0	0	0	0	0	0	0	0	15	0	0	0	0	0	0	0	0	15	0	0	12	0	0	2
400	10	0	0	0	0	0	0	0	0	0	0	0	0	0	0	0	0	0	8	0	0	0	0	0	5	0	0	40	0	0	1
