# Supplementary material for: Bacteria in the oral cavity of individuals consuming intoxicating substances
Source: PLoS One. 2023 May 26;18(5):e0285753. doi: 10.1371/journal.pone.0285753 (PMC10218728; doi:10.1371/journal.pone.0285753)
Supplement: S4 Table — (PDF) [file pone.0285753.s004.pdf]

S4-Table: Record of health issues reported by the participants.

PARTICIPANT ID	HEALTH ISSUES (1 = YES, 0 = NO)															
	MOUTH_ULCER	GINGIVITIS	PERIODONTITIS	ORAL CANCER	ORAL THRUSH	ACUTE_CELLULITIS	GRANULOMA	OSTEOMYLITIS	APPETITE_CONDITION	DROWSINESS	INSOMNIA	NAUSEA	MYALGIA	ARTHRALGIA	VISION	ANOREXIA	HEADACHE
101	0	0	0	0	0	0	0	0	0	1	0	0	1	1	1	0	1
102	0	0	0	0	1	0	0	0	0	1	0	0	0	0	1	1	1
103	1	1	0	0	0	0	0	0	0	1	1	0	0	0	1	0	1
104	1	1	1	0	0	1	1	0	0	1	0	0	0	1	0	0	1
105	1	0	0	0	1	1	1	0	0	1	0	0	0	1	0	1	1
106	0	0	0	0	0	0	0	0	0	0	0	0	0	0	1	0	1
107	0	0	0	0	0	0	0	0	0	0	0	0	0	0	0	0	1
108	0	0	0	0	1	0	0	0	0	0	0	0	0	0	0	0	1
109	0	0	0	0	0	0	0	0	0	1	0	0	0	0	0	1	1
110	1	0	1	0	1	1	0	0	0	1	1	0	1	0	0	1	1
111	1	1	0	0	1	0	0	0	0	1	0	1	0	0	0	0	1
112	0	0	0	0	0	0	0	0	0	1	0	0	0	0	0	0	0
113	0	0	0	0	1	0	0	0	0	0	0	0	0	0	0	1	1
114	0	0	0	0	1	0	0	0	0	0	1	0	0	0	0	0	1
115	1	0	1	0	0	1	0	0	0	0	0	0	1	1	0	1	1
116	0	0	0	0	0	0	0	0	0	1	1	0	1	0	0	0	1
117	0	0	0	0	0	0	0	0	0	0	0	0	1	0	1	0	1
118	1	0	0	0	1	0	0	1	0	1	1	0	0	1	0	0	1
119	0	0	0	0	1	0	0	0	0	0	0	0	0	0	0	0	1
120	0	0	0	0	0	1	0	0	0	0	0	0	0	0	1	0	1
121	0	0	0	0	1	1	0	0	0	0	0	0	0	0	0	0	0
122	0	0	0	0	0	0	0	0	0	0	0	0	1	0	0	0	1
123	1	0	0	0	1	0	1	0	0	1	0	0	0	0	0	1	0
124	0	0	1	0	0	0	0	0	0	0	1	1	1	0	1	0	1
125	1	0	0	0	1	0	0	0	0	0	0	0	0	0	0	1	1
126	1	0	0	0	1	1	1	1	0	0	1	0	0	0	1	0	1
127	1	0	0	0	1	0	0	0	0	1	0	0	1	0	0	1	1

128	0	0	0	0	0	0	0	0	0	0	0	1	0	0	0	0	1
129	0	0	0	0	0	0	0	1	0	1	1	0	0	0	0	1	1
130	0	0	0	0	1	0	0	0	0	0	0	1	0	1	0	1	1
131	0	0	0	0	0	1	1	0	0	1	0	0	0	0	0	0	1
132	1	1	1	0	1	1	1	0	1	1	0	1	0	1	0	1	1
133	1	1	1	0	1	0	0	0	0	1	0	0	0	0	0	0	0
134	1	0	0	0	1	0	0	0	0	1	0	0	0	0	0	1	1
135	0	1	0	0	0	1	0	0	0	0	0	0	1	0	1	0	1
136	1	0	0	0	1	0	0	0	0	1	0	0	0	0	0	0	1
137	1	0	0	0	1	0	0	0	0	1	0	0	0	0	0	1	1
138	1	0	0	0	1	1	1	0	1	1	1	1	1	1	0	0	1
139	1	1	1	0	1	0	1	0	0	1	0	0	0	0	0	1	0
140	0	0	1	0	1	0	0	0	0	0	0	0	1	1	0	0	1
141	0	0	0	0	0	0	0	0	0	0	0	0	0	0	0	1	0
142	0	0	0	0	0	0	0	0	0	0	0	1	0	0	0	1	1
143	1	0	0	0	0	0	0	0	0	1	0	0	1	1	1	1	1
144	0	0	0	0	1	0	0	0	0	1	1	0	0	0	1	0	1
145	0	0	0	0	1	0	0	0	0	1	0	0	0	0	0	1	1
146	0	0	0	0	0	0	0	0	0	0	0	0	0	0	0	0	0
147	0	1	0	0	0	0	0	0	0	1	0	0	1	0	1	0	1
148	1	0	0	0	0	0	0	0	0	0	0	0	0	0	1	0	1
149	0	0	0	0	0	0	0	1	0	1	0	1	1	0	0	0	1
150	0	0	0	0	0	0	0	0	0	0	0	0	1	0	0	0	1
151	0	0	0	0	0	0	0	0	0	1	0	0	0	0	0	0	1
152	1	1	0	0	0	0	0	1	0	1	0	0	0	1	1	0	1
153	1	1	0	0	0	0	1	1	0	1	0	0	0	1	1	1	0
154	0	1	1	0	0	1	1	0	0	0	1	1	1	1	0	1	1
155	1	0	0	0	0	0	0	1	0	1	1	0	0	0	1	0	1
156	0	0	0	0	0	0	0	0	1	1	0	0	0	0	1	0	1
157	0	0	0	0	0	0	0	0	0	1	1	0	1	1	1	0	1
158	0	1	0	0	0	0	0	0	0	1	0	1	0	0	0	0	1
159	0	0	0	0	0	0	0	0	1	1	1	0	0	0	0	1	1

160	0	0	0	0	0	0	0	0	0	0	0	0	1	0	0	0	0
161	0	1	1	0	1	1	0	0	1	0	0	0	1	0	1	0	1
162	0	0	0	0	1	0	0	0	1	1	1	1	1	1	0	0	1
163	0	1	0	0	1	1	1	0	1	1	1	0	1	1	1	1	1
164	0	0	0	0	1	1	0	0	1	0	0	0	0	0	1	0	0
165	0	1	0	0	1	1	1	0	1	1	1	0	0	0	0	0	1
166	0	0	0	0	1	0	0	0	0	0	0	0	0	0	0	1	0
167	1	1	0	0	1	1	0	0	0	0	0	0	0	0	1	1	1
168	0	0	0	0	1	0	0	0	0	1	0	1	1	1	1	1	1
169	0	0	1	0	1	0	0	0	0	0	0	0	0	0	0	0	0
170	0	1	1	0	1	1	1	1	0	1	0	0	0	1	1	0	1
171	0	0	1	0	1	0	0	1	1	1	1	1	1	1	1	0	1
172	0	0	0	0	1	0	0	0	0	1	0	1	0	0	1	0	1
173	1	0	0	0	1	1	0	0	1	1	1	1	0	1	1	0	1
174	0	0	0	0	1	0	0	0	0	1	0	0	0	0	1	0	0
175	0	0	0	0	1	0	0	0	1	1	0	0	0	1	1	1	1
176	0	0	0	0	1	0	0	0	0	1	0	0	0	0	0	0	1
177	0	0	0	0	1	0	0	0	0	1	0	1	0	0	1	0	1
178	0	0	1	0	1	0	0	0	0	1	1	1	1	0	1	1	1
179	0	0	0	0	1	0	0	0	0	0	0	0	0	0	1	1	0
180	0	1	1	0	1	1	0	0	0	0	0	0	0	0	1	0	0
181	0	0	0	0	1	1	1	0	0	0	0	0	0	0	0	0	0
182	0	0	0	0	1	1	0	0	0	0	0	0	0	0	0	0	0
183	1	1	0	0	1	0	1	1	0	0	0	0	0	0	1	0	0
184	0	0	0	0	1	1	1	0	0	1	0	0	1	0	1	0	0
185	0	1	0	0	0	0	1	1	0	1	0	0	0	1	0	1	1
186	0	1	0	0	1	1	0	0	0	1	0	0	0	0	0	0	0
187	0	1	0	0	0	0	0	0	0	0	0	0	0	0	0	1	1
188	0	0	0	0	1	0	0	0	0	0	0	0	0	0	0	0	0
189	0	0	0	0	0	0	1	0	0	1	0	0	0	1	0	1	1
190	1	0	0	0	1	1	0	1	1	1	0	0	1	1	0	1	1
191	1	1	0	0	1	0	1	0	0	1	1	1	0	0	1	0	1

192	0	1	1	0	1	1	1	1	0	1	1	0	0	0	1	0	1
193	0	0	0	0	1	0	0	0	0	0	0	0	1	1	0	0	0
194	0	0	0	0	0	0	0	0	0	0	0	0	0	0	1	0	1
195	0	0	0	0	1	0	0	0	0	0	0	0	0	1	0	0	1
196	0	1	0	0	1	1	1	0	0	1	0	1	1	1	1	1	0
197	0	1	0	0	1	0	1	0	0	1	0	1	1	1	1	0	1
198	0	0	0	0	0	0	1	0	0	0	0	0	0	0	0	0	0
199	0	0	0	0	0	1	1	0	0	0	0	0	0	0	1	0	1
200	0	0	0	0	1	0	0	0	0	0	0	0	0	0	1	0	0
201	0	0	0	0	1	0	0	0	1	1	0	1	1	1	0	0	0
202	0	0	0	0	1	0	1	0	0	0	1	0	0	0	0	1	0
203	0	1	0	0	1	1	0	1	0	1	1	1	1	1	1	0	1
204	0	0	0	0	1	0	0	0	0	0	0	1	1	1	0	0	1
205	0	0	0	0	0	0	1	0	0	0	0	0	0	0	0	1	0
206	0	0	0	0	1	0	0	0	0	1	0	0	0	0	0	0	0
207	0	0	0	0	1	1	0	0	0	0	1	1	0	0	1	0	1
208	0	1	0	0	1	1	1	1	0	1	1	0	1	1	0	0	0
209	0	0	0	0	1	0	0	0	0	1	0	0	1	1	1	0	1
210	0	0	0	0	1	0	0	0	0	1	1	0	1	1	0	0	1
211	0	1	1	0	1	0	0	0	0	1	0	0	0	0	1	0	1
212	0	0	0	0	1	0	0	1	1	1	0	0	0	0	0	0	1
213	0	1	0	0	0	0	1	0	1	1	0	1	0	0	0	0	1
214	0	0	0	0	1	1	0	0	0	1	1	1	1	0	0	1	1
215	0	0	0	0	1	1	0	0	0	1	0	0	1	1	1	0	0
216	1	1	0	0	1	1	1	1	1	1	1	1	1	1	1	0	1
217	0	1	1	0	1	1	0	1	0	1	0	1	1	1	1	0	1
218	0	1	1	0	1	1	0	0	1	0	0	0	1	1	0	0	0
219	0	0	0	0	1	1	0	1	0	1	1	1	1	1	1	0	1
220	0	0	0	0	1	0	0	1	0	1	0	1	1	0	1	0	1
221	0	0	0	0	1	0	0	0	1	1	1	0	1	0	0	0	1
222	0	1	0	0	1	1	0	0	1	1	0	1	0	1	0	0	1
223	0	1	1	0	1	1	0	0	0	1	0	1	1	1	0	0	1

224	0	1	0	0	1	0	0	1	0	1	0	0	1	1	0	0	1
225	0	0	0	0	1	0	0	0	0	1	0	0	1	0	0	0	1
226	0	0	0	0	1	0	0	0	0	1	0	0	0	0	1	0	1
227	0	0	0	0	1	0	0	0	0	1	1	0	0	1	0	0	1
228	0	0	0	0	0	0	1	0	0	1	0	0	1	0	0	0	1
229	0	0	0	0	0	0	0	0	0	1	0	0	1	0	1	0	1
230	0	0	0	0	1	1	0	0	0	1	0	0	0	0	0	0	0
231	0	0	0	0	0	0	0	0	0	0	0	0	0	0	0	0	1
232	0	0	0	0	0	0	0	0	0	1	0	1	1	1	0	0	1
233	0	0	0	0	0	0	0	0	0	0	0	0	0	0	1	0	1
234	0	1	0	0	1	1	1	1	0	0	0	1	1	0	1	0	1
235	0	0	0	0	1	0	0	0	0	1	0	1	1	0	1	0	1
236	0	0	0	0	0	0	0	0	0	1	0	1	0	1	1	0	0
237	0	0	0	0	0	0	0	0	0	1	0	0	0	0	1	0	1
238	0	0	0	0	0	0	0	0	0	1	0	0	0	0	0	0	0
239	0	0	0	0	0	0	0	0	0	0	0	0	0	0	1	1	1
240	0	1	0	0	1	0	0	0	0	1	0	1	0	0	1	1	1
241	0	0	0	0	0	0	0	0	1	0	0	0	0	0	0	0	0
242	1	1	0	0	0	0	0	0	0	0	0	0	0	0	0	0	0
243	0	0	0	0	0	0	0	0	1	1	0	0	1	0	0	0	1
244	0	1	0	0	1	1	0	1	0	0	0	1	1	0	0	0	1
245	1	0	0	0	1	0	1	0	0	0	0	0	0	0	0	0	0
246	0	1	0	0	1	0	0	0	0	0	0	0	0	0	0	0	0
247	1	0	0	0	1	0	0	0	1	0	0	1	0	0	0	0	1
248	0	0	0	0	0	1	0	0	0	1	0	1	0	1	0	0	0
249	1	1	0	0	1	0	0	0	1	1	0	1	1	0	0	0	1
250	0	0	0	0	1	0	0	0	0	1	1	0	0	0	0	0	1
251	1	0	0	0	1	0	0	0	0	0	0	0	0	0	0	0	0
252	0	0	0	0	1	0	0	0	0	1	0	1	0	0	1	0	1
253	0	1	0	0	1	0	1	0	1	1	1	1	1	1	1	1	1
254	0	0	0	0	0	0	0	0	0	0	0	0	0	0	0	1	1
255	0	0	0	0	1	0	0	0	0	0	0	0	0	0	1	0	0

256	1	1	0	0	1	0	0	0	0	1	1	0	0	1	0	0	1
257	0	0	0	0	1	1	0	0	0	1	1	0	0	1	1	0	0
258	0	0	0	0	1	0	0	0	0	1	0	0	0	0	0	0	1
259	0	0	0	0	1	0	1	0	1	1	0	1	1	1	1	1	1
260	0	0	0	0	1	0	0	0	0	0	0	0	0	0	1	0	1
261	0	0	0	0	1	0	0	0	1	1	0	0	0	0	0	0	1
262	0	0	0	0	1	0	0	0	0	1	0	0	0	1	1	0	0
263	0	1	0	0	1	0	0	1	0	1	0	0	0	1	0	0	1
264	0	0	0	0	1	0	0	0	0	0	0	0	0	1	0	0	0
265	0	0	0	0	0	0	0	0	0	0	0	1	0	0	0	0	0
266	1	0	0	0	1	1	0	0	0	1	0	0	0	0	0	0	1
267	0	0	0	0	1	0	1	0	0	0	1	0	0	0	0	1	1
268	0	0	0	0	0	0	0	0	0	0	0	0	0	0	0	0	1
269	0	0	0	0	0	0	0	0	0	0	1	0	0	0	0	0	1
270	0	0	0	0	1	0	0	0	1	0	1	0	0	0	0	0	0
271	0	0	0	0	0	0	0	0	0	1	0	1	1	1	0	1	1
272	1	1	0	0	0	1	0	0	0	1	0	0	1	0	0	1	0
273	0	0	0	0	0	0	0	0	0	1	0	0	0	0	0	0	0
274	0	0	0	0	0	0	0	0	0	0	0	0	0	0	0	1	1
275	0	1	0	0	1	1	0	1	0	1	0	0	0	0	0	1	1
276	0	0	0	0	1	0	1	0	0	0	0	0	0	0	0	0	1
277	0	0	1	0	1	0	0	0	0	0	0	0	0	0	0	1	1
278	0	0	0	0	1	0	0	0	0	1	0	0	1	0	0	0	0
279	0	0	0	0	0	0	0	0	0	0	0	0	0	0	0	0	0
280	0	0	1	0	1	0	1	0	0	0	0	0	0	0	0	0	0
281	0	0	0	0	0	0	0	0	0	0	0	0	0	0	0	0	1
282	0	0	0	0	0	0	1	0	0	0	0	0	0	0	0	0	1
283	0	0	0	0	0	0	0	0	0	0	0	0	0	0	0	0	0
284	0	1	1	0	1	0	0	0	0	1	1	1	0	0	1	1	1
285	1	0	0	0	0	0	1	0	0	0	0	0	0	0	0	1	1
286	0	0	0	0	1	0	1	0	0	0	0	0	0	0	0	0	0
287	0	0	0	0	1	1	0	0	0	1	1	0	0	0	0	1	1

288	0	1	0	0	1	1	0	0	0	0	0	0	0	0	0	0	0
289	0	0	0	0	0	0	0	0	0	0	0	0	0	0	0	0	1
290	0	0	0	0	0	0	0	0	0	0	0	0	0	0	0	0	1
291	0	0	0	0	0	0	0	0	0	0	0	0	0	0	0	0	1
292	0	0	0	0	0	0	0	0	0	0	0	0	0	0	0	0	1
293	0	0	0	0	0	0	0	0	0	0	0	0	0	0	0	0	1
294	0	0	0	0	1	0	0	0	0	0	0	0	0	0	0	0	1
295	0	0	0	0	1	0	0	0	0	0	0	0	0	0	0	0	1
296	0	0	0	0	0	0	0	0	0	0	0	0	0	0	0	0	1
297	0	0	0	0	1	0	0	0	0	0	0	0	0	0	0	0	1
298	0	0	0	0	0	0	0	0	0	0	0	0	0	0	0	0	1
299	0	0	0	0	0	0	0	0	0	0	0	0	0	0	0	0	0
300	0	0	0	0	0	0	0	0	0	0	0	0	0	0	0	0	0
301	0	0	0	0	1	0	0	0	0	0	0	0	0	0	0	0	1
302	0	0	0	0	1	0	0	0	0	0	0	0	0	0	0	0	1
303	0	0	0	0	0	0	0	0	0	0	0	0	0	0	0	0	1
304	0	0	0	0	0	0	0	0	0	0	0	0	0	0	0	0	0
305	0	0	0	0	0	0	0	0	0	0	0	0	0	0	0	0	1
306	0	0	0	0	1	0	0	0	0	0	0	0	0	0	0	0	1
307	0	0	0	0	1	0	0	0	0	0	0	0	0	0	0	0	1
308	0	1	1	0	0	0	0	0	0	0	0	0	0	0	1	1	1
309	0	0	0	0	1	0	1	0	0	0	0	1	1	0	1	0	1
310	0	0	0	0	0	0	0	0	0	0	0	0	0	0	0	0	1
311	0	0	0	0	0	0	0	0	0	0	0	0	0	0	1	0	1
312	0	0	0	0	0	0	0	0	0	0	0	0	0	0	1	0	1
313	0	0	0	0	1	0	0	0	0	0	0	0	0	0	1	0	1
314	0	0	0	0	0	0	0	0	0	0	0	0	0	0	1	0	1
315	0	0	0	0	0	0	0	0	0	0	0	0	0	0	1	1	0
316	0	0	0	0	1	0	0	0	0	0	0	0	0	0	0	0	1
317	0	0	0	0	0	0	0	0	0	1	0	1	1	0	0	0	1
318	0	1	0	0	1	0	0	0	0	0	0	0	0	0	0	1	0
319	0	0	0	0	0	0	0	0	0	0	0	0	0	0	1	0	1

320	0	0	0	0	1	0	0	0	0	0	0	0	0	0	1	0	0
321	0	0	0	0	1	0	0	0	0	0	0	0	0	0	0	0	0
322	0	0	0	0	0	0	0	0	0	0	0	0	0	0	0	0	1
323	0	0	0	0	0	0	0	0	0	0	0	0	0	0	0	0	1
324	0	0	0	0	1	0	0	0	0	0	0	0	0	0	0	0	1
325	0	1	1	0	1	0	0	0	0	0	0	1	0	0	0	1	1
326	1	0	0	0	0	0	0	1	0	1	0	0	0	0	0	0	1
327	0	1	0	0	1	1	1	0	0	1	0	0	0	0	1	0	1
328	1	1	1	0	0	0	0	0	0	0	0	0	0	0	1	0	0
329	0	0	0	0	1	0	0	1	0	0	0	0	0	0	0	0	1
330	0	0	0	0	1	0	0	0	0	0	0	0	0	0	0	0	1
331	0	0	0	0	1	0	0	0	0	1	0	0	0	0	1	0	1
332	1	1	1	0	1	1	0	0	0	1	1	0	0	0	0	1	1
333	1	0	0	0	0	0	0	0	0	1	0	0	0	0	1	0	1
334	0	0	0	0	0	0	1	0	0	1	1	1	0	0	0	0	1
335	0	0	0	0	0	0	0	0	0	1	1	0	0	0	0	0	1
336	1	0	0	0	0	0	0	0	0	1	0	0	0	0	1	0	1
337	0	0	0	0	0	0	0	0	0	0	0	0	0	0	0	0	0
338	0	0	0	0	1	0	0	0	0	0	0	0	0	0	0	0	0
339	0	0	0	0	1	0	0	0	0	1	0	0	0	0	0	0	1
340	0	0	0	0	1	0	0	0	0	1	0	0	0	0	0	0	0
341	0	0	0	0	0	0	0	0	0	0	0	0	0	0	0	0	0
342	0	0	0	0	0	0	0	0	0	0	1	1	0	0	1	0	1
343	0	0	0	0	0	0	0	0	0	0	0	1	0	0	0	1	1
344	0	0	0	0	0	0	0	0	0	0	1	0	0	0	0	1	1
345	0	1	0	0	1	0	0	1	0	0	0	0	0	0	0	0	0
346	0	0	0	0	1	0	0	0	0	0	0	0	0	0	0	0	0
347	0	0	0	0	1	0	0	0	0	0	0	0	0	0	1	0	0
348	1	1	0	0	1	1	1	1	0	1	0	1	0	1	0	0	1
349	0	0	0	0	1	0	0	0	0	0	0	0	0	0	0	1	1
350	0	0	0	0	1	0	0	0	0	0	0	0	0	0	0	1	0
351	0	0	0	0	1	0	0	0	0	0	0	1	1	1	0	0	1

352	0	0	0	0	1	1	1	0	0	0	0	0	0	0	0	0	0
353	1	0	1	0	0	0	0	0	0	0	0	1	0	0	0	0	1
354	0	0	0	0	1	0	1	0	0	1	0	0	1	0	0	1	1
355	0	0	0	0	0	1	0	0	0	0	0	1	1	0	0	1	1
356	0	1	0	0	1	0	0	0	0	0	0	0	0	0	0	0	1
357	0	0	0	0	0	0	0	0	0	0	0	0	0	0	0	0	0
358	0	1	1	0	1	0	0	0	0	0	0	0	0	0	0	0	1
359	0	0	0	0	0	0	0	0	0	0	0	0	0	0	0	0	0
360	0	0	0	0	1	0	0	0	0	0	0	0	0	0	0	0	0
361	0	0	0	0	0	0	0	0	0	0	1	0	0	0	0	0	1
362	0	0	0	0	0	0	0	0	0	1	1	0	0	0	0	0	1
363	0	0	0	0	0	0	0	0	1	1	1	0	0	1	1	1	1
364	0	0	0	0	1	0	0	0	0	0	0	0	0	0	0	0	0
365	0	0	0	0	0	0	0	0	0	0	0	0	1	0	0	0	1
366	0	0	0	0	1	0	0	0	1	0	1	0	0	1	0	1	1
367	0	0	0	0	0	0	0	0	0	0	0	0	0	0	0	0	0
368	0	0	1	0	1	0	0	0	1	1	0	0	1	1	1	0	0
369	0	0	0	0	0	0	1	0	0	0	0	0	0	0	0	0	0
370	0	0	0	0	1	0	0	0	1	0	1	0	0	0	0	0	0
371	0	0	1	0	1	0	0	0	0	1	0	0	0	0	0	0	0
372	0	0	0	0	0	0	0	0	0	0	0	0	0	1	0	0	1
373	0	0	0	0	1	0	0	0	0	1	1	0	0	1	0	0	1
374	0	0	0	0	1	0	0	0	1	1	1	1	0	0	0	0	1
375	0	1	0	0	0	0	1	0	0	0	0	0	0	0	0	0	1
376	0	0	0	0	0	0	0	0	0	0	0	0	0	0	0	1	1
377	0	0	0	0	1	0	0	0	0	0	0	0	0	0	0	1	0
378	1	0	0	0	0	0	0	0	0	0	0	0	0	1	0	1	1
379	0	0	0	0	1	0	0	0	0	0	0	0	0	0	1	0	1
380	1	0	0	0	1	1	0	0	1	0	1	0	0	1	1	0	0
381	0	0	0	0	1	0	0	0	0	0	0	0	0	0	0	0	1
382	0	0	1	0	1	0	1	0	1	1	1	0	0	0	0	0	1
383	1	0	0	0	1	1	1	0	0	0	0	0	0	0	1	1	0

384	0	0	0	0	1	0	0	0	1	1	1	0	0	0	0	0	1
385	0	0	1	0	1	0	0	0	1	1	0	0	0	0	0	0	1
386	1	1	1	1	0	1	1	0	1	1	0	1	0	0	0	0	1
387	1	0	0	1	1	0	0	1	0	0	0	0	1	1	1	0	0
388	0	1	0	1	1	0	0	0	0	1	0	0	0	0	1	0	0
389	0	0	0	1	1	1	0	0	0	0	0	0	0	0	1	0	0
390	0	0	0	1	1	1	0	0	0	0	0	0	0	0	0	0	0
391	1	1	1	1	1	1	1	0	0	0	1	0	1	0	0	0	1
392	0	0	0	1	1	0	0	0	0	1	1	1	0	0	0	0	0
393	1	0	0	1	0	0	1	1	0	0	0	1	1	0	1	0	1
394	0	0	0	1	1	1	0	1	0	1	1	0	1	0	1	0	1
395	0	0	0	1	1	0	0	0	0	0	0	0	0	0	1	0	0
396	0	0	0	1	1	0	0	1	0	1	1	1	0	0	1	1	1
397	1	1	1	1	0	1	0	0	0	0	0	0	0	0	1	0	1
398	0	0	0	1	0	0	0	0	0	0	0	0	1	1	0	0	0
399	0	0	0	1	1	1	1	0	0	1	0	0	0	0	0	1	0
400	1	0	0	1	1	1	0	0	0	0	0	0	0	0	0	0	0
