# Supplementary material for: Bacteria in the oral cavity of individuals consuming intoxicating substances
Source: PLoS One. 2023 May 26;18(5):e0285753. doi: 10.1371/journal.pone.0285753 (PMC10218728; doi:10.1371/journal.pone.0285753)
Supplement: S5 Table — (PDF) [file pone.0285753.s005.pdf]

S5-Table: Record on microbes isolated from swab.									
**PARTICIPANT ID**	**MICROBES ISOLATED (1 = YES, 0 = NO)**								
	**Staphylococcus (B1)**	**Bacillus (B2)**	**Serratia (B3)**	**Rhodococcus (B4)**	**Pseudomonus (B5)**	**Acinetobacter (B6)**	**Enterobacter (B7)**	**Candida (F1)**	**Klebsiella (B8)**
101	1	1	0	0	0	0	0	0	0
102	1	0	0	0	0	0	0	1	0
103	1	0	0	0	0	0	0	0	0
104	0	0	0	0	1	0	0	0	0
105	0	0	0	0	1	0	0	0	0
106	1	1	0	0	0	0	0	0	0
107	1	1	0	0	0	0	0	0	0
108	1	0	0	0	0	0	0	0	0
109	1	0	0	0	0	0	0	0	0
110	1	0	0	0	0	0	0	0	0
111	0	1	0	0	0	0	0	0	0
112	1	0	0	0	0	0	0	0	0
113	0	1	0	0	0	0	0	0	0
114	1	1	0	0	0	0	0	0	0
115	0	1	0	0	0	0	0	0	0
116	0	1	0	0	0	0	0	0	0
117	1	0	0	0	0	0	0	0	0
118	1	1	0	0	0	0	0	0	0
119	1	0	0	0	0	0	0	0	0
120	0	1	0	0	0	0	0	0	0
121	0	1	0	0	1	0	0	0	0
122	0	1	0	0	0	0	0	0	0
123	0	1	0	0	1	0	0	0	0
124	1	1	0	0	0	0	0	0	0
125	1	1	0	0	0	0	0	0	0
126	0	1	0	0	0	0	0	0	0
127	1	1	0	0	0	0	0	0	0
128	1	1	0	0	0	0	0	0	0

129	1	0	0	0	0	0	0	0	0
130	1	1	0	0	0	0	0	0	0
131	0	1	0	0	0	0	0	0	0
132	1	0	0	0	0	0	0	0	0
133	1	1	1	0	1	0	0	0	0
134	1	0	0	0	0	0	0	0	0
135	0	1	0	0	0	0	0	0	0
136	1	0	0	0	0	0	0	0	0
137	1	0	0	0	0	0	0	0	0
138	0	0	0	0	1	0	0	0	0
139	1	0	0	0	0	0	0	0	0
140	0	1	0	0	0	0	0	0	0
141	0	1	0	0	1	0	0	0	0
142	1	1	0	0	0	0	0	0	0
143	0	1	0	0	0	0	0	0	0
144	0	1	0	0	1	0	0	0	0
145	0	1	0	0	0	0	0	0	0
146	0	1	0	0	0	0	0	0	0
147	1	1	0	0	1	0	0	0	0
148	1	0	1	0	0	0	1	0	0
149	1	1	0	0	0	0	0	0	0
150	1	1	0	0	0	0	0	0	0
151	0	0	0	0	1	0	0	0	0
152	1	1	0	0	0	0	0	0	0
153	1	1	0	0	0	0	0	0	0
154	0	0	1	0	0	0	0	0	0
155	1	1	0	0	0	0	0	0	0
156	1	0	0	0	0	0	0	0	0
157	1	0	0	1	1	0	0	0	0
158	0	1	0	0	0	0	0	0	0
159	1	0	0	0	0	0	0	0	0
160	1	1	0	0	0	0	0	0	0

161	0	0	0	0	0	0	0	0	1
162	1	0	0	0	0	0	0	0	1
163	0	1	0	0	0	0	0	0	0
164	0	0	0	0	1	0	0	0	0
165	0	1	0	0	0	0	0	0	0
166	1	0	1	0	0	0	0	0	0
167	1	0	0	0	1	0	0	0	0
168	0	1	1	0	0	0	0	0	0
169	0	0	1	0	0	0	0	0	0
170	1	1	0	0	0	0	0	0	0
171	0	1	0	0	0	0	0	0	0
172	1	1	0	0	0	0	0	0	0
173	0	1	0	0	0	0	0	0	0
174	1	0	0	0	0	0	0	0	0
175	1	0	0	0	0	0	0	0	0
176	1	1	0	0	0	0	0	0	0
177	1	1	0	0	0	0	0	0	0
178	1	1	0	0	0	0	0	0	0
179	1	0	0	0	0	0	0	0	0
180	0	1	0	0	0	0	0	0	0
181	1	1	0	0	1	0	0	0	0
182	1	0	0	0	0	0	0	0	0
183	1	1	0	0	0	0	0	0	0
184	1	0	0	0	0	0	0	0	0
185	0	1	0	0	0	0	0	0	0
186	0	1	0	0	0	1	0	0	0
187	1	0	0	0	0	0	0	0	0
188	1	0	0	0	0	0	0	0	0
189	0	0	0	0	1	0	0	0	0
190	0	1	0	0	1	0	0	0	0
191	1	1	0	0	0	0	0	0	0
192	0	1	0	0	0	0	0	0	0

193	1	0	0	0	0	0	0	0	0
194	1	1	0	0	0	0	0	0	0
195	0	1	0	0	0	0	0	0	0
196	1	1	0	0	0	0	0	0	0
197	0	1	0	0	0	0	0	0	0
198	1	0	0	0	0	0	0	0	0
199	0	1	0	0	0	0	0	0	0
200	0	0	0	0	1	0	0	0	0
201	1	0	0	0	0	0	0	0	0
202	0	1	0	0	0	0	0	0	0
203	1	0	0	0	0	0	0	0	0
204	1	0	0	0	0	1	0	0	0
205	0	0	0	0	0	0	1	0	0
206	1	0	0	0	0	0	0	0	0
207	1	1	0	0	0	0	0	0	0
208	1	1	0	0	1	0	0	0	0
209	1	1	0	0	0	0	0	0	0
210	1	0	0	0	0	0	0	0	0
211	1	1	0	0	0	0	0	0	0
212	0	0	0	0	1	0	0	0	0
213	0	0	0	0	1	0	0	0	0
214	0	0	0	0	1	0	0	0	0
215	1	1	0	0	0	0	0	0	0
216	0	0	0	0	1	0	0	0	0
217	1	0	0	0	0	0	0	0	0
218	0	1	1	0	1	0	0	0	0
219	1	1	1	0	0	0	0	0	0
220	0	1	0	0	0	0	0	0	0
221	0	0	0	0	1	0	0	0	0
222	1	1	0	0	0	0	0	0	0
223	1	1	0	0	0	0	0	0	0
224	0	1	0	0	0	0	0	0	0

225	0	1	0	0	0	0	0	0	0
226	0	0	0	0	1	0	0	0	0
227	1	0	0	0	1	0	0	0	0
228	0	1	0	0	0	0	0	0	0
229	1	1	0	0	0	0	1	0	0
230	0	1	0	0	0	0	0	0	0
231	0	1	0	0	0	0	0	0	0
232	0	1	0	0	1	0	0	0	0
233	1	1	0	0	0	0	0	0	0
234	0	0	0	0	1	0	1	0	0
235	1	1	0	0	0	0	0	0	0
236	0	1	0	0	1	0	0	0	0
237	0	1	0	0	0	0	0	0	0
238	0	0	0	0	0	0	1	0	0
239	0	1	0	0	1	0	0	0	0
240	0	1	0	0	1	0	0	0	0
241	1	0	0	0	0	0	0	0	0
242	1	0	0	0	0	0	0	0	0
243	1	0	0	0	0	0	0	0	0
244	1	1	0	0	0	0	0	0	0
245	1	0	0	0	0	0	0	0	0
246	1	1	0	0	0	0	0	0	0
247	1	1	0	0	0	0	0	0	0
248	1	1	0	0	0	0	0	0	0
249	1	1	0	0	0	0	0	0	0
250	0	0	1	0	0	1	0	0	0
251	0	1	0	0	0	0	0	0	0
252	1	0	0	0	0	0	0	0	0
253	0	1	0	0	0	0	1	0	0
254	1	1	1	0	0	0	0	0	0
255	0	1	0	0	0	0	0	0	0
256	0	1	1	0	0	0	0	0	0

257	1	1	0	0	0	0	0	0	0
258	1	1	0	0	0	0	0	0	0
259	1	1	0	0	0	0	0	0	0
260	1	0	0	0	0	0	0	0	0
261	1	0	0	0	1	0	0	0	0
262	1	1	0	0	0	0	0	0	0
263	0	1	0	0	0	0	0	0	0
264	1	0	0	0	0	0	0	0	0
265	1	1	0	0	0	0	0	0	0
266	1	1	0	0	0	0	0	0	0
267	1	1	0	0	0	0	0	0	0
268	1	0	0	0	0	0	0	0	0
269	1	0	0	0	0	0	0	0	0
270	1	1	0	0	0	0	0	0	0
271	1	0	0	0	0	0	0	0	0
272	1	0	0	0	0	0	0	0	0
273	0	1	0	0	0	0	0	0	0
274	0	0	0	0	0	0	1	0	0
275	1	0	0	0	0	0	0	0	0
276	0	1	0	0	0	0	0	0	0
277	0	1	0	0	0	0	0	0	0
278	1	0	0	0	0	0	0	0	0
279	0	1	0	0	0	0	1	0	0
280	0	1	0	0	1	0	0	0	0
281	0	1	0	0	0	0	0	0	0
282	1	1	1	0	0	0	0	0	0
283	1	1	0	0	0	0	0	0	0
284	1	1	0	0	0	0	0	0	0
285	1	1	0	0	0	0	0	0	0
286	0	1	0	0	0	0	0	0	0
287	0	1	0	0	1	0	0	0	0
288	0	0	0	0	1	0	0	0	0

289	1	1	0	0	0	0	0	0	0
290	1	1	0	0	0	0	0	0	0
291	0	1	0	0	1	0	0	0	0
292	1	1	0	0	0	0	0	0	0
293	1	1	0	0	0	1	0	0	0
294	1	1	0	0	0	0	1	1	0
295	1	1	0	0	0	0	0	0	0
296	1	1	1	0	0	0	0	1	0
297	1	0	0	0	0	0	0	0	0
298	1	0	0	0	0	0	0	0	0
299	1	1	0	0	0	0	0	0	0
300	1	1	0	0	0	0	0	0	0
301	0	1	0	0	0	0	0	0	0
302	1	1	0	0	0	0	0	0	0
303	1	1	0	0	0	0	0	0	0
304	1	1	0	0	0	0	0	0	0
305	1	1	0	0	0	0	0	0	0
306	1	1	0	0	0	0	0	0	0
307	1	1	0	0	1	0	0	0	0
308	1	1	0	0	1	0	0	0	0
309	0	1	0	0	1	0	0	0	0
310	1	1	0	0	0	0	0	0	0
311	1	1	0	0	0	0	0	0	0
312	1	0	0	0	0	0	0	0	0
313	1	0	0	0	1	0	0	0	0
314	1	1	0	0	1	0	0	0	0
315	1	0	0	0	0	0	0	0	0
316	0	0	0	0	1	0	0	0	0
317	1	1	0	0	0	0	0	0	0
318	1	0	0	0	0	0	0	0	0
319	0	0	0	0	1	0	0	0	0
320	1	1	0	0	1	0	0	0	0

321	1	0	0	0	0	0	0	0	0
322	1	1	0	0	0	0	0	0	0
323	1	0	0	0	0	0	0	0	0
324	0	1	0	0	0	0	0	0	0
325	1	1	0	0	0	0	0	0	0
326	1	0	0	0	0	0	0	0	0
327	1	0	0	0	0	0	0	0	0
328	1	1	0	0	0	0	0	0	0
329	1	1	0	0	0	0	0	0	0
330	1	1	0	0	1	0	0	0	0
331	1	1	0	0	0	0	0	0	0
332	1	0	0	0	0	0	0	0	0
333	1	0	0	0	0	0	0	0	0
334	1	1	0	0	0	0	0	0	0
335	0	1	0	0	1	0	0	0	0
336	1	0	0	0	0	0	0	1	0
337	1	1	0	0	1	0	0	0	0
338	1	1	0	0	0	0	0	0	0
339	1	0	0	0	0	0	1	0	0
340	1	0	0	0	1	0	1	0	0
341	1	1	0	0	0	0	1	0	0
342	1	1	0	0	0	0	0	0	0
343	0	1	0	0	0	0	0	0	0
344	1	1	0	0	0	0	0	0	0
345	1	1	1	0	0	0	0	0	0
346	1	1	0	0	0	0	0	0	0
347	1	1	0	0	0	0	0	0	0
348	1	1	0	0	0	0	0	0	0
349	1	1	1	0	0	0	0	0	0
350	1	0	1	0	0	0	0	0	0
351	1	0	0	0	0	0	0	0	0
352	1	0	0	0	0	0	0	0	0

353	0	1	0	0	0	0	0	0	0
354	1	0	1	0	0	0	0	0	0
355	1	0	0	0	0	0	0	0	0
356	1	1	0	0	0	0	0	0	0
357	0	1	0	0	0	0	0	0	0
358	1	0	0	0	0	0	0	0	0
359	1	0	0	0	0	0	0	0	0
360	0	1	0	0	0	0	0	0	0
361	1	0	0	0	0	0	0	0	0
362	1	0	0	0	0	0	0	0	0
363	1	0	0	0	0	0	0	0	0
364	1	1	0	0	0	0	0	0	0
365	1	1	0	0	0	0	0	0	0
366	0	1	1	0	0	0	0	0	0
367	0	1	0	0	0	0	0	0	0
368	0	1	0	0	1	0	0	0	0
369	0	1	1	0	0	0	0	0	0
370	1	1	0	0	0	0	0	0	0
371	0	1	0	0	1	0	0	0	0
372	0	1	0	0	0	0	0	0	0
373	1	1	0	0	0	0	0	0	0
374	1	1	0	0	0	0	0	0	0
375	1	1	0	0	0	0	0	0	0
376	1	0	0	0	1	0	0	0	0
377	1	0	0	0	1	0	0	0	0
378	0	0	1	0	0	0	0	0	0
379	1	1	0	0	0	0	0	0	0
380	1	1	0	0	0	0	0	0	0
381	1	0	0	0	0	0	0	1	0
382	1	0	0	0	0	0	0	0	0
383	0	0	1	0	0	0	0	0	0
384	1	1	0	0	0	0	0	0	0

385	1	0	0	0	0	0	0	0	0
386	1	1	0	0	0	0	0	0	0
387	1	1	0	0	0	0	0	0	0
388	0	1	0	0	0	0	0	0	0
389	1	0	1	0	0	0	0	0	0
390	1	1	0	0	0	0	0	0	0
391	1	1	0	0	0	0	0	0	0
392	0	1	0	0	0	0	0	0	0
393	0	0	0	0	0	0	0	1	0
394	1	0	0	0	0	0	0	1	0
395	1	0	0	0	0	0	1	0	0
396	0	1	0	0	1	0	0	0	0
397	0	1	0	0	0	0	1	0	0
398	1	0	0	0	0	0	0	0	0
399	1	0	0	0	1	0	0	0	0
400	0	0	0	0	1	0	0	0	0

**Organisms**	**Isolated from No. of swab samples**
Staphylococcus (B1)	196
Bacillus (B2)	186
Serrat ia (B3)	22
Rhodococcus (B4)	1
Pseudomonus (B5)	55
Acinetobacter (B6)	4
Enterobacter (B7)	14
Candida (F1)	7
Klebsiella (B8)	2
