# Supplementary material for: Bacteria in the oral cavity of individuals consuming intoxicating substances
Source: PLoS One. 2023 May 26;18(5):e0285753. doi: 10.1371/journal.pone.0285753 (PMC10218728; doi:10.1371/journal.pone.0285753)
Supplement: S6 Table — (PDF) [file pone.0285753.s006.pdf]

S6-Table: Descriptive statistics of the samples/participants of different age groups, consumers and non-consumers of intoxicating substances.

**Intoxication Type**	**Sole Betel Nut Consumers**			**Sole Gutkha Consumers**			**Sole Smokers**			**Sole Alcohol Consumers**			**Combined Intoxication**			**Non Consumers**			**Grand Total**
Age group (years)	Male	Female	Total	Male	Female	Total	Male	Female	Total	Male	Female	Total	Male	Female	Total	Male	Female	Total	
19-24	9 (3.0%)	20 (6.7%)	**29 (9.7%)**	3 (1.0%)	1 (0.3%)	**4 (1.3%)**	1 (0.3%)	-	**1 (0.3%)**	1 (0.3%)	1 (0.3%)	**2 (0.7%)**	31 (10.3%)	6 (2.0%)	**37 (12.3%)**	22 (7.3%)	11 (3.7%)	**33 (11%)**	**106 (35.3%)**
25-30	5 (1.7%)	14 (4.7%)	**19 (6.3%)**	2 (0.7%)	-	**2 (0.7%)**	1 (0.3%)	1 (0.3%)	**2 (0.7%)**	2 (0.7%)	-	**2 (0.7%)**	25 (8.3%)	2 (0.7%)	**27 (9.0%)**	20 (6.7%)	19 (6.3%)	**39 (13%)**	**91 (30.3%)**
31-36	3 (1.0%)	7 (2.3%)	**10 (3.3%)**	-	-	-	-	-	-	-	-	-	9 (3.0%)	2 (0.7%)	**11 (3.7%)**	10 (3.3%)	3 (1%)	**13 (4.3%)**	**34 (11.3%)**
37-42	2 (0.7%)	13 (4.3%)	**15 (5.0%)**	-	-	-	-	-	-	-	-	-	6 (2.0%)	1 (0.3%)	**7 (2.3%)**	1 (0.3%)	1 (0.3%)	**2 (0.7%)**	**24 (8.0%)**
43-48	2 (0.7%)	6 (2.0%)	**8 (2.7%)**	-	-	-	-	-	-	-	-	-	7 (2.3%)	1 (0.3%)	**8 (2.7%)**	1 (0.3%)	-	**1 (0.3%)**	**17 (5.7%)**
49-54	-	1 (0.3%)	**1 (0.3%)**	-	-	-	-	-	-	-	-	-	8 (2.7%)	1 (0.3%)	**9 (3.0%)**	1 (0.3%)	-	**1 (0.3%)**	**11 (3.7%)**
55-60	4 (1.3%)	6 (2.0%)	**10 (3.3%)**	-	-	-	-	-	-	-	-	-	7 (2.3%)	-	**7 (2.3%)**	-	-	-	**17 (5.7%)**
Total	25 (8.3%)	67 (22.3%)	**92 (30.7%)**	5 (1.7%)	1 (0.3%)	**6 (2.0%)**	2 (0.7%)	1 (0.3%)	**3 (1.0%)**	3 (1.0%)	1 (0.3%)	**4 (1.3%)**	93 (31%)	13 (4.3%)	**106 (35.3%)**	55 (18.3%)	34 (11.3%)	**89 (29.7%)**	**300 (100%)**
