# Supplementary material for: Bacteria in the oral cavity of individuals consuming intoxicating substances
Source: PLoS One. 2023 May 26;18(5):e0285753. doi: 10.1371/journal.pone.0285753 (PMC10218728; doi:10.1371/journal.pone.0285753)
Supplement: S7 Table — (PDF) [file pone.0285753.s007.pdf]

S7-Table: **Oral cavity cleanliness record of the participants (male and female) grouped by age.**

Brushing times/Day	0			1			2			3			Grand Total
**Age group (Years)**	Male	Female	Total	Male	Female	Total	Male	Female	Total	Male	Female	Total	
**19-24**	2 (0.7%)	0	**2 (0.7%)**	44 (14.7%)	21 (7%)	**65 (21.7%)**	20 (6.7%	18 (6%)	**38 (12.7%)**	1 (0.3%)	0	**1 (0.3%)**	**106 35.3%)**
**25-30**	0	0	**0**	37 (12.3%)	20 (6.7%)	**57 (19%)**	17 (5.7%)	15 (5%)	**32 (10.7%)**	1 (0.3%)	1 (0.3%)	**2 (0.7%)**	**91 (30.3%)**
**31-36**	0	0	**0**	13 (4.3%)	3 (1%)	**16 (5.3%)**	9 (3%)	9 (3%)	**18** (6%)	0	0	**0**	**34 (11.3%)**
**37-42**	0	0	**0**	6 (2%)	5 (1.7%)	**11 (3.7%)**	3 (1%)	7 (2.3%)	**10 (3.3%)**	0	3 (1%)	**3** (1%)	**24 (8%)**
**43-48**	0	0	**0**	7 (2.3%)	1 (0.3%)	**8 (2.7%)**	3 (1%)	4 (1.3%)	**7 (2.3%)**	0	2 (0.7%)	**2 (0.7%)**	**17 (5.7%)**
**49-54**	0	0	**0**	7 (2.3%)	1 (0.3%)	**8 (2.7%)**	2 (0.7%)	1 (0.3%)	**3** (1%)	0	0	**0**	**11 (3.7%)**
**55-60**	0	0	**0**	7 (2.3%)	3 (1%)	**10 (3.3%)**	4 (1.3%)	2 (0.7%)	**6** (2%)	0	1 (0.3%)	**1 (0.3%)**	**17 (5.7%)**
**Total**	2 (0.7%)	0	**2 (0.7%)**	121 (40.3%)	54 (18%)	**175 (58.3%)**	58 (19.3%)	56 (18.7%)	**114 (38%)**	2 (0.7%)	7 (2.3%)	**9 (3%)**	**300 (100%)**
