# Supplementary material for: Bacteria in the oral cavity of individuals consuming intoxicating substances
Source: PLoS One. 2023 May 26;18(5):e0285753. doi: 10.1371/journal.pone.0285753 (PMC10218728; doi:10.1371/journal.pone.0285753)
Supplement: S8 Table — (PDF) [file pone.0285753.s008.pdf]

S8-Table: Risk of incidence of Microbes when exposed to intoxicating substances.								
**Organism**	**Intoxicating Substances**	**B**	**S.E.**	**Wald**	**Sig.**	**95% C.I. for EXP(B)**		
						**Upper**	**Lower**	**Exp(B)**
B1(*Staphylococcus*)	Betel nut	0.564	0.246	5.256	0.022	2.849	1.085	1.759
	Smoking	0.402	0.179	5.041	0.025	2.121	1.052	1.494
	Non consumers	-.405	.183	4.890	.027	.955	.466	.667
B2(*Bacillus*)	Betel nut	0.557	0.175	10.071	0.002	2.461	1.237	1.745
	Betel nut with lime	0.941	0.326	8.303	0.004	4.857	1.351	2.562
	Non consumers	-.368	.193	3.651	.056	1.010	.474	.692
B3 (*Serratia*)	Betel nut	-0.994	0.421	5.578	0.018	0.844	0.162	0.37
	Zarda pan	1.759	0.616	8.148	0.004	0.576	0.051	0.172
	Smoking	1.74	0.285	37.175	0	0.307	0.1	0.175
	Alcohol	2.024	0.743	7.42	0.006	0.567	0.031	0.132
B4 (*Rhodococcus*)	Betel nut	1.892	1.074	3.099	0.078	1.239	0.018	0.151
	Mitha pan	3.113	1.022	9.274	0.002	0.33	0.006	0.044
	Smoking	4.622	1.02	20.526	0	0.073	0.001	0.01
B5 (*Pseudomonas*)	Betel nut	-0.521	0.303	2.961	0.085	1.075	0.328	0.594
	Zarda pan	1.27	0.498	6.499	0.011	0.746	0.106	0.281
	Gutkha	-0.893	0.486	3.381	0.066	1.061	0.158	0.409
	Sikhar	1.086	0.531	4.19	0.041	8.383	1.047	2.963
	Smoking	1.191	0.225	28.001	0	0.473	0.196	0.304
	Non consumers	-.865	.328	6.948	.008	.801	.221	.421
B6 (*Acinetobacter*)	Mitha pan	2.717	1.032	6.926	0.008	0.5	0.009	0.066
	Gutkha	1.612	0.771	4.367	0.037	0.905	0.044	0.199
	Smoking	4.339	0.712	37.124	0	0.053	0.003	0.013
B7 *(Enterobacter)*	Mitha pan	1.426	0.636	5.034	0.025	0.835	0.069	0.24
	Gutkha	-0.836	0.669	1.56	0.212	1.609	0.117	0.434
	Smoking	2.585	0.302	73.277	0	0.136	0.042	0.075
B8 (*Klebsiella*)	Mitha pan	2.694	1.034	6.791	0.009	0.513	0.009	0.068
	Gutkha	2.522	1.039	5.889	0.015	0.616	0.01	0.08
	Smoking	4.449	0.711	39.106	0	0.047	0.003	0.012
F1 (*Candida*)	Betel nut	1.221	0.689	3.141	0.076	1.138	0.076	0.295
	Zarda pan	1.105	1.103	1.003	0.317	2.878	0.038	0.331
	Mitha pan	1.421	0.777	3.345	0.067	1.107	0.053	0.242
	Smoking	2.586	0.448	33.287	0	0.181	0.031	0.075

The table include odd ratio which significantly contributes to the regression.

CI Confidence interval, S.E. Standard error, B Coefficient, EXP (*B*) Odds ratio, Sig. Significance level
