# Supplementary material for: Bacteria in the oral cavity of individuals consuming intoxicating substances
Source: PLoS One. 2023 May 26;18(5):e0285753. doi: 10.1371/journal.pone.0285753 (PMC10218728; doi:10.1371/journal.pone.0285753)
Supplement: S9 Table — (PDF) [file pone.0285753.s009.pdf]

S9-Table: Risk of health issues when exposed to intoxicating substances.

Health Conditions	Intoxicating Substances	B	S.E.	Wald	Sig.	95% C.I. for EXP(B)		
						Upper	Lower	Exp(B)
Acute cellulites (AC)	Betel nut	-0.567	0.196	8.361	0.004	0.833	0.386	0.567
	Gutkha	-1.105	0.423	6.839	0.009	0.758	0.145	0.331
	Sikhar	0.942	0.473	3.973	0.046	6.482	1.016	2.566
	Smoking	-0.757	0.389	3.79	0.052	1.005	0.219	0.469
	Non consumers	-2.456	.740	11.019	.001	.366	.020	.086
Anorexia (AN)	Betel nut	-1.408	0.233	36.478	0	0.386	0.155	0.245
	Betel nut with lime	0.87	0.377	5.326	0.021	4.997	1.14	2.387
Appetite condition (APC)	Betel nut	-1.117	0.235	22.565	0	0.519	0.206	0.327
	Mitha pan	-1.411	0.427	10.924	0.001	0.563	0.106	0.244
	Smoking	-0.94	0.513	3.361	0.067	1.067	0.143	0.391
Arthralgia (AR)	Betel nut	-0.679	0.196	12	0.001	0.745	0.345	0.507
	Sikhar	-1.017	0.469	4.71	0.03	0.906	0.144	0.362
	Smoking	-1.163	0.435	7.152	0.007	0.733	0.133	0.312
Drowsiness (D)	Betel nut	0.317	0.161	3.898	0.048	1.881	1.002	1.373
	Non consumers	-1.334	.323	17.064	.000	.496	.140	.263
Gingivitis (G)	Betel nut	-0.729	0.189	14.8	0	0.699	0.333	0.482
	Gutkha	-0.672	0.312	4.625	0.032	0.942	0.277	0.511
Granuloma (GL)	Betel nut	-1.302	0.193	45.309	0	0.397	0.186	0.272
Headache (H)	Betel nut	0.515	0.226	5.207	0.022	2.604	1.075	1.673
	Betel nut with lime	0.705	0.415	2.887	0.089	4.569	0.897	2.025
	Mitha pan	1.169	0.33	12.553	0	6.149	1.686	3.22
	Smoking	-0.817	0.356	5.276	0.022	0.887	0.22	0.442
	Non consumers	.546	.235	5.421	.020	2.735	1.090	1.727
Insomnia (INS)	Betel nut	-1.146	0.244	22.096	0	0.513	0.197	0.318
	Betel nut with lime	0.684	0.386	3.15	0.076	4.221	0.931	1.982
	Mitha pan	-0.719	0.307	5.49	0.019	0.889	0.267	0.487
Mouth ulceration (MU)	Betel nut	-1.124	0.184	37.182	0	0.466	0.226	0.325
	Non consumers	-1.140	.516	4.874	.027	.880	.116	.320
Myalgia (M)	Betel nut	-0.537	0.198	7.337	0.007	0.862	0.397	0.585
	Mitha pan	-0.751	0.301	6.243	0.012	0.851	0.262	0.472
	Sikhar	-0.67	0.402	2.775	0.096	1.126	0.233	0.512
Nausea (N)	Betel nut	-0.7	0.19	13.617	0	0.72	0.342	0.496
	Gutkha	-0.96	0.332	8.368	0.004	0.734	0.2	0.383
	Non consumers	-.873	.449	3.780	.052	1.007	.173	.418
Oral cancer (OC)	Betel nut	-2.134	0.351	37.043	0	0.235	0.06	0.118
	Mitha pan	-1.912	0.561	11.597	0.001	0.444	0.049	0.148
	Gutkha	-2.427	0.68	12.747	0	0.335	0.023	0.088
	Tobacco	2.317	0.595	15.159	0	32.583	3.161	10.148
Oral thrush (OT)	Betel nut	1.171	0.219	28.53	0	4.959	2.099	3.226
	Mitha pan	-0.886	0.289	9.394	0.002	0.727	0.234	0.412
	Sikhar	0.676	0.383	3.117	0.077	4.164	0.928	1.966
Osteomyelitis (OS)	Betel nut	-1.333	0.24	30.726	0	0.422	0.165	0.264
	Mitha pan	-1.271	0.399	10.126	0.001	0.614	0.128	0.281
	Sikhar	-1.257	0.556	5.121	0.024	0.845	0.096	0.284
Periodontitis (P)	Betel nut	-1.12	0.235	22.794	0	0.517	0.206	0.326
	Mitha pan	-0.648	0.341	3.6	0.058	1.022	0.268	0.523
	Gutkha	-1.487	0.45	10.944	0.001	0.545	0.094	0.226
Vision (V)	Betel nut with lime	1.888	0.421	20.126	0	15.077	2.896	6.608
	Mitha pan	-0.552	0.29	3.616	0.057	1.017	0.326	0.576
	Sikhar	-1.769	0.495	12.748	0	0.45	0.065	0.171

	Smoking	-1.051	0.394	7.109	0.008	0.757	0.161	0.35
	Non consumers	-.639	.341	3.501	.061	1.031	.270	.528

The table include odd ratio which significantly contributes to the regression.

CI Confidence interval, S.E. Standard error, B Coefficient, EXP (*B*) Odds ratio, Sig. Significance level
