# Supplementary material for: Bacteria in the oral cavity of individuals consuming intoxicating substances
Source: PLoS One. 2023 May 26;18(5):e0285753. doi: 10.1371/journal.pone.0285753 (PMC10218728; doi:10.1371/journal.pone.0285753)
Supplement: S10 Table — (PDF) [file pone.0285753.s010.pdf]

S10-Table: Risk of health issues when exposed to Microorganisms.								
**Health condition**	**Organisms**	**B**	**S.E.**	**Wald**	**Sig.**	**95% C.I. for EXP(B)**		
						**Upper**	**Lower**	**Exp(B)**
Acute cellulites (AC)	B1(*Staphylococcus*)	-1.143	0.211	29.395	0.000	0.482	0.211	0.319
	B2(*Bacillus*)	-0.848	0.21	16.288	0.000	0.646	0.284	0.428
Anorexia (AN)	B1(*Staphylococcus*)	-0.792	0.2	15.74	0.000	0.67	0.306	0.453
	B2(*Bacillus*)	-1.107	0.215	26.546	0.000	0.504	0.217	0.33
	B3(*Serrat ia*)	0.836	0.469	3.179	0.075	5.778	0.92	2.306
Appetite condition (APC)	B1(*Staphylococcus*)	-1.481	0.248	35.754	0.000	0.369	0.14	0.227
	B2(*Bacillus*)	-1.511	0.259	33.91	0.000	0.367	0.133	0.221
Arthralgia (AR)	B1(Staphylococcus)	-1.132	0.212	28.477	0.000	0.489	0.213	0.322
	B2(*Bacillus*)	-0.505	0.212	5.704	0.017	0.913	0.399	0.603
	B5(*Pseudomonas*)	-0.67	0.348	3.711	0.054	1.012	0.259	0.512
	B7(*Enterobacter*)	-1.851	1.053	3.093	0.079	1.236	0.02	0.157
Drowsiness (D)	B2(*Bacillus*)	-0.254	0.151	2.835	0.092	1.043	0.577	0.776
	B3(*Serrat ia*)	-0.846	0.486	3.032	0.082	1.112	0.166	0.429
Gingivitis (G)	B1(*Staphylococcus*)	-1.051	0.205	26.204	0.000	0.523	0.234	0.35
	B2(*Bacillus*)	-0.544	0.207	6.878	0.009	0.872	0.386	0.58
	B5(*Pseudomonas*)	-0.727	0.343	4.483	0.034	0.947	0.246	0.483
Granuloma (GL)	B1(*Staphylococcus*)	-1.473	0.23	40.893	0.000	0.36	0.146	0.229
	B2(*Bacillus*)	-0.833	0.216	14.841	0.000	0.664	0.285	0.435
Headache (H)	B1(*Staphylococcus*)	0.414	0.185	4.998	0.025	2.176	1.052	1.513
	B2(*Bacillus*)	0.618	0.191	10.46	0.001	2.696	1.275	1.854
Insomnia (INS)	B1(*Staphylococcus*)	-1.005	0.21	22.887	0.000	0.552	0.242	0.366
	B2(*Bacillus*)	-0.676	0.216	9.816	0.002	0.776	0.333	0.509
	B5(*Pseudomonas*)	-0.86	0.368	5.45	0.020	0.871	0.206	0.423
	B7(*Enterobacter*)	-1.81	1.053	2.955	0.086	1.289	0.021	0.164
Mouth ulceration (MU)	B1(*Staphylococcus*)	-0.961	0.211	20.721	0.000	0.578	0.253	0.382
	B2(*Bacillus*)	-1.03	0.227	20.505	0.000	0.558	0.229	0.357
	B5(*Pseudomonas*)	-0.931	0.382	5.94	0.015	0.833	0.186	0.394
Myalgia (M)	B1(*Staphylococcus*)	-0.921	0.201	20.959	0.000	0.591	0.268	0.398
	B2(*Bacillus*)	-0.609	0.207	8.64	0.003	0.816	0.363	0.544
	B5(*Pseudomonas*)	-0.71	0.344	4.272	0.039	0.964	0.251	0.491
Nausea (N)	B1(*Staphylococcus*)	-1.063	0.208	26.007	0.000	0.52	0.23	0.345
	B2(*Bacillus*)	-0.388	0.209	3.461	0.063	1.021	0.451	0.678
	B3(*Serrat ia*)	-1.116	0.641	3.026	0.082	1.152	0.093	0.328
	B5(*Pseudomonas*)	-1.009	0.365	7.625	0.006	0.746	0.178	0.365
Oral cancer (OC)	B1(*Staphylococcus*)	-2.224	0.353	39.587	0.000	0.216	0.054	0.108
	B2(*Bacillus*)	-2.1	0.377	31.036	0.000	0.256	0.058	0.122
	B5(*Pseudomonas*)	-1.834	0.617	8.842	0.003	0.535	0.048	0.16
Oral thrush (OT)	B1(*Staphylococcus*)	0.331	0.148	4.994	0.025	1.861	1.042	1.392
	B5(*Pseudomonas*)	0.695	0.296	5.502	0.019	3.581	1.121	2.003
Osteomyelitis (OS)	B1(*Staphylococcus*)	-1.604	0.255	39.41	0.000	0.332	0.122	0.201
	B2(*Bacillus*)	-1.069	0.249	18.346	0.000	0.56	0.211	0.343
	B5(*Pseudomonas*)	-1.31	0.452	8.396	0.004	0.655	0.111	0.27
Periodontitis (P)	B1(*Staphylococcus*)	-1.627	0.255	40.603	0.000	0.324	0.119	0.197
	B2(*Bacillus*)	-1.024	0.246	17.341	0.000	0.581	0.222	0.359
	B5(*Pseudomonas*)	-1.135	0.426	7.105	0.008	0.741	0.14	0.322
Vision (V)	B1(*Staphylococcus*)	-0.555	0.182	9.252	0.002	0.821	0.402	0.574

	B2(*Bacillus*)	-0.353	0.186	3.605	0.058	1.011	0.488	0.703

The table include odd ratio which significantly contributes to the regression.

CI Confidence interval, S.E. Standard error, B Coefficient, EXP (*B*) Odds ratio, Sig. Significance level
