# Supplementary figures and images for: Bacteria in the oral cavity of individuals consuming intoxicating substances
Source: PLoS One. 2023 May 26;18(5):e0285753. doi: 10.1371/journal.pone.0285753 (PMC10218728; doi:10.1371/journal.pone.0285753)

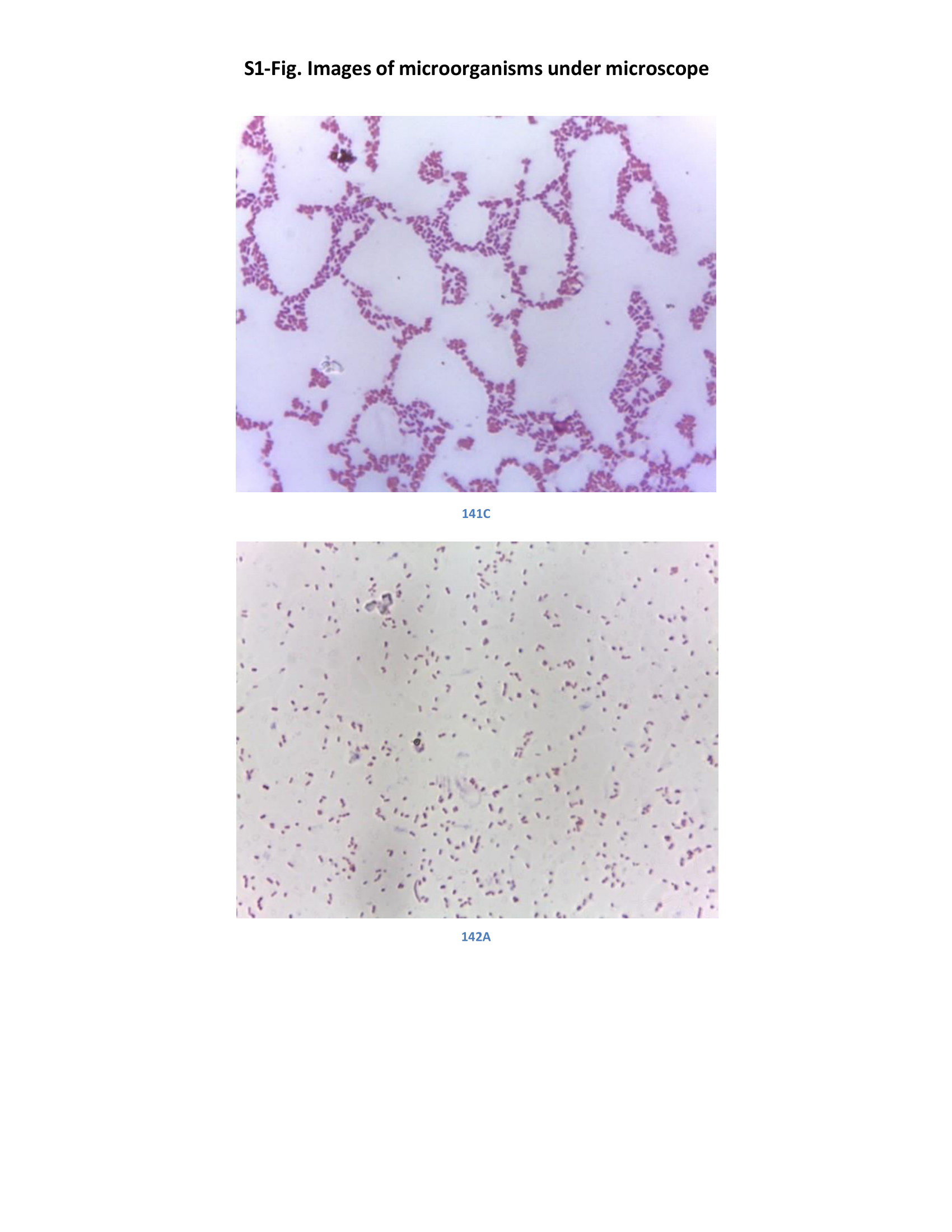

Supplement: S1 Fig — (TIFF) [file pone.0285753.s011.tiff]

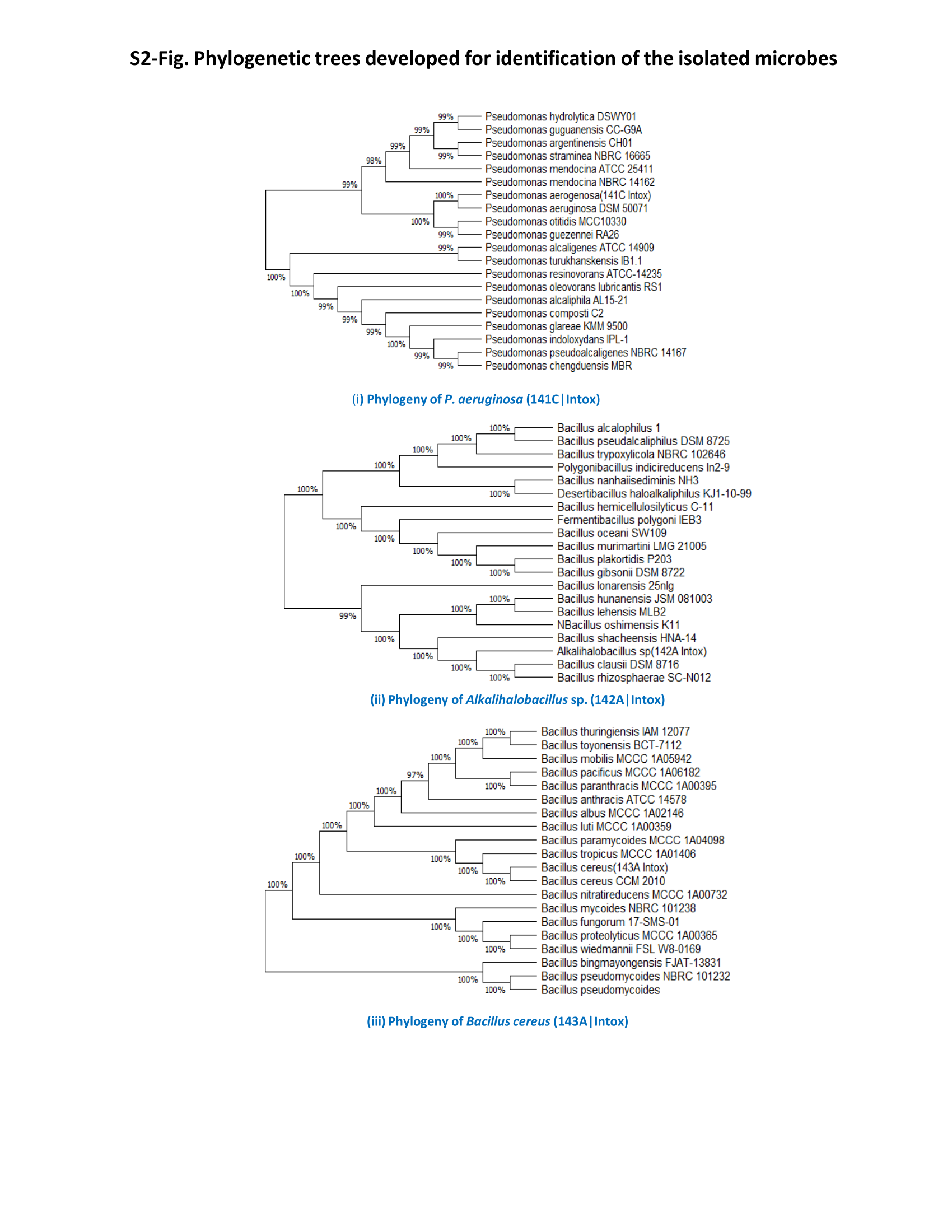

Supplement: S2 Fig — (TIFF) [file pone.0285753.s012.tiff]
